# Deciphering the physiological response of *Escherichia coli* under high ATP demand

**DOI:** 10.15252/msb.202110504

**Published:** 2021-12-20

**Authors:** Simon Boecker, Giulia Slaviero, Thorben Schramm, Witold Szymanski, Ralf Steuer, Hannes Link, Steffen Klamt

**Affiliations:** ^1^ Analysis and Redesign of Biological Networks Max Planck Institute for Dynamics of Complex Technical Systems Magdeburg Germany; ^2^ Dynamic Control of Metabolic Networks Max Planck Institute for Terrestrial Microbiology Marburg Germany; ^3^ Interfaculty Institute for Microbiology and Infection Medicine Tübingen University of Tübingen Tübingen Germany; ^4^ Core Facility for Mass Spectrometry and Proteomics Max Planck Institute for Terrestrial Microbiology Marburg Germany; ^5^ Institute for Biology Humboldt‐University of Berlin Berlin Germany

**Keywords:** ATP homeostasis, central metabolism, glycolysis, kinetic model, metabolic engineering, Metabolism, Microbiology, Virology & Host Pathogen Interaction

## Abstract

One long‐standing question in microbiology is how microbes buffer perturbations in energy metabolism. In this study, we systematically analyzed the impact of different levels of ATP demand in *Escherichia coli* under various conditions (aerobic and anaerobic, with and without cell growth). One key finding is that, under all conditions tested, the glucose uptake increases with rising ATP demand, but only to a critical level beyond which it drops markedly, even below wild‐type levels. Focusing on anaerobic growth and using metabolomics and proteomics data in combination with a kinetic model, we show that this biphasic behavior is induced by the dual dependency of the phosphofructokinase on ATP (substrate) and ADP (allosteric activator). This mechanism buffers increased ATP demands by a higher glycolytic flux but, as shown herein, it collapses under very low ATP concentrations. Model analysis also revealed two major rate‐controlling steps in the glycolysis under high ATP demand, which could be confirmed experimentally. Our results provide new insights on fundamental mechanisms of bacterial energy metabolism and guide the rational engineering of highly productive cell factories.

## Graphical abstract

A systematic analysis of the impact of different levels of ATP demand on *E. coli* under various conditions reveals a biphasic response curve of the glucose uptake rate with respect to increasing ATP demand.

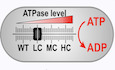

## Introduction

The sugar and energy metabolism of microorganisms has been subject of research for many decades (Jensen & Michelsen, 1992; Kochanowski *et al*, 2013; Chubukov *et al*, 2014; Basan *et al*, 2020). One central goal of these studies is to decipher key principles of cellular metabolism and to uncover regulatory mechanisms that enable microorganisms to adapt to perturbations and varying environments (Chubukov *et al*, 2014; Bruggeman *et al*, 2020). The integration of various experimental data, often in combination with mathematical modeling, helps to shed light on global phenomena of microbial metabolism, such as overflow metabolism or diauxie (Basan *et al*, 2015, 2020; Chen & Nielsen, 2019; Bruggeman *et al*, 2020). However, despite the progress made, a comprehensive understanding of how microbes respond and adapt to perturbations is still lacking in many cases. This also limits our ability to rationally engineer the metabolism of microorganisms for biotechnological applications. One example of directed metabolic interventions to optimize microbial production hosts is the manipulation of the supply of ATP, the energy currency of the cell. Increasing ATP availability can, for example, lead to improved succinate (Zhang *et al*, 2009; Singh *et al*, 2011) or recombinant protein (Kim *et al*, 2012) production. In the opposite direction, artificially enforcing a high turnover (“wasting”) of ATP can substantially increase the specific glucose uptake rate and the production rate of certain target compounds (if production of the latter is coupled with ATP formation) (Chao & Liao, 1994; Koebmann *et al*, 2002; Hädicke *et al*, 2015; Liu *et al*, 2016; Boecker *et al*, 2019, 2021; Zahoor *et al*, 2020).

Studying the response of the cells to perturbed ATP levels is thus essential not only for understanding fundamental physiological processes but also for guiding metabolic engineering efforts. As one approach, several previous studies investigated the influence of a continuous drain of ATP on the metabolism of *Escherichia coli* (Chao & Liao, 1994; Koebmann *et al*, 2002; Holm *et al*, 2010). However, more systematic studies, especially with varying levels of ATP demand under different growth conditions, are still needed to address fundamental questions, for example, to which extent the cells are able to compensate a rising ATP drain by increasing the glucose uptake rate. In particular, it is unknown what the maximal glucose uptake rate is and what happens when the ATP drain is further increased beyond this point.

In this study, we systematically analyzed the consequences of varying levels of ATP turnover in *E. coli* by overexpressing the genes of the ATP‐hydrolyzing F_1_‐subunit of the F_O_F_1_‐ATPase under different conditions (aerobic and anaerobic conditions, cell growth and growth arrest). As one key result, we found that the glucose uptake rate shows under all conditions a biphasic response curve with respect to increasing ATPase activity, reaching a maximum at a medium ATPase level but dropping markedly when this level is exceeded. Focusing on anaerobic growth, we combined metabolome and proteome data with a kinetic model of *E. coli*’s central metabolism to reveal the underlying mechanism of this behavior. Analysis of the model showed that the dual dependency of the phosphofructokinase on ATP as substrate and ADP as activator can explain the biphasic steady‐state response curve of the glycolytic flux. The model also helped to explain unexpected phenomena such as the accumulation of glycolytic metabolites, and it suggested two major rate‐controlling steps under high ATP drain, which were confirmed experimentally by overexpressing the genes of the associated metabolic enzymes.

## Results

### Construction of the ATPase strains with different ATPase expression strengths

As in previous studies (Koebmann *et al*, 2002; Holm *et al*, 2010; Boecker *et al*, 2019), as ATP‐consuming mechanism, we chose the *atpAGD‐*encoded F_1_‐subunit of the F_O_F_1_‐ATP synthase (ATPase) from *E. coli*, which hydrolyzes ATP to ADP and phosphate. We decided to regulate the expression strength via different origins of replication and thus varying copy numbers of the plasmids harboring the ATPase genes. Three different plasmids were constructed: a low copy plasmid (RK2 replicon, LC), a medium copy plasmid (p15A replicon, MC), and a high copy plasmid (pMB1 replicon, HC) (Appendix Table S1). *E. coli* wild‐type strain MG1655 was transformed with the three plasmids as well as with the corresponding empty control plasmids leading to the six strains “LC control”, “LC ATPase”, “MC control”, “MC ATPase”, “HC control”, and “HC ATPase”. Expression of *atpAGD* was put under control of the isopropyl β‐d‐thiogalactopyranoside (IPTG) inducible *P_trc_*‐promoter, and the same amount of IPTG was used for all strains. Additional controls were the *E. coli* wild‐type strain MG1655 without plasmid and without IPTG addition (“WT”) and without plasmid but with IPTG addition (“WT + IPTG”).

### Anaerobic growth

**Figure 1:**
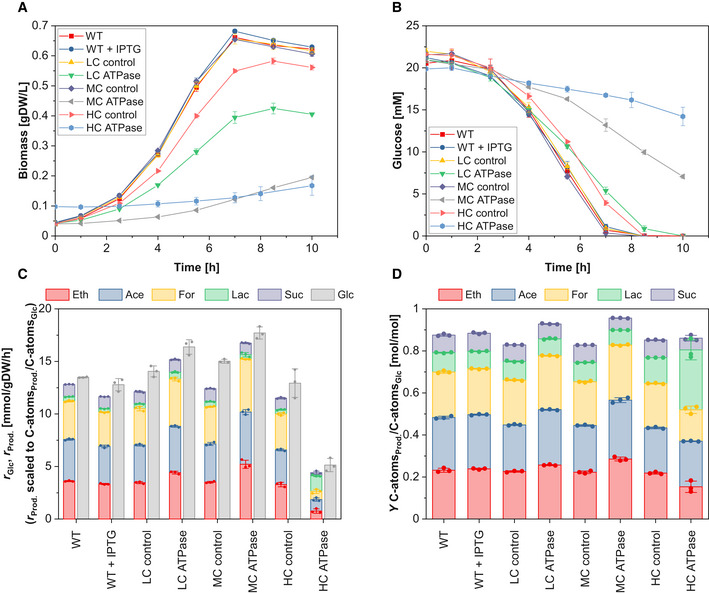
Anaerobic growth of the different strains Time course of biomass concentration.Time course of glucose concentration.Specific glucose (Glc) uptake rate and specific productivity for ethanol (Eth), acetate (Ace), formate (For), lactate (Lac), and succinate (Suc).Yield of Eth, Ace, For, Lac, and Suc. Data information: The reaction rates in (C) were calculated for the exponential phase under assumption of quasi‐steady state. As changes in glucose and fermentation products are rather small during cultivation of the HC ATPase strain, a higher initial biomass concentration of 0.1 gDW/l was used for this strain to get more robust data for the calculation of the metabolite exchange rates. The means (A and B) and the means and individual data (C and D) for *n* = 3 biologically independent samples are shown. The error bars represent ± SD. Source data are available online for this figure.

First, we cultivated all strains anaerobically and monitored the effect of expressing ATPase on growth rate, glucose uptake, and production of the five main fermentation products (ethanol, acetate, formate, lactate, and succinate) in the exponential phase. As expected, the growth rates and biomass yields of the three ATPase strains decreased with increasing expression levels of the ATPase (Fig 1A–D; Table 1). Furthermore, compared to their control strains, the specific glucose uptake rate of the LC and MC ATPase strain increased by 16.6% to 16.4 mmol_Glc_/gDW/h and by 17.8% to 17.7 mmol_Glc_/gDW/h, respectively. Similarly, the specific production rates of fermentation products were elevated, and their cumulated yield increased by 11.4% to 0.93 mol_product C‐atoms_/mol_Glc C‐atoms_ in the LC ATPase and by 16.7% to 0.97 mol_product C‐atoms_/mol_Glc C‐atoms_ in the MC ATPase strain (Table 1). This indicates that a larger portion of the substrate was redirected from biomass to energy production (and thus to the formation of fermentation products) in these two ATPase strains to keep up with the higher cellular ATP demand. However, the data from the HC ATPase strain with highest ATPase level suggest that there is a maximal ATPase activity, beyond which *E*. *coli* cannot compensate the ATP drain with even higher glycolytic rates. The specific glucose uptake rate in the HC ATPase strain dropped by 60.3% to 5.1 mmol_Glc_/gDW/h, which is far below the WT strain level. Apparently, there are limitations in *E. coli*’s metabolism that prevent a further increase in the glycolytic flux needed to counterbalance the high ATPase activity. Using a stoichiometric model of the central metabolism of *E. coli*, we performed metabolic flux analysis, based on the measured exchange rates, to estimate the ATPase flux in the different strains (see Materials and Methods). Importantly, while the ATPase flux increased with higher ATPase abundance from LC to MC ATPase strain, we determined the lowest ATPase flux among all ATPase strains for the HC ATPase strain (Table 1). In fact, the markedly reduced substrate uptake rate already implies that the ATP hydrolysis rate must be lower in this strain. However, if less ATP is consumed than in the other two strains, this raises the question why the glucose uptake rate drops so strongly. A hypothesis is that the HC ATPase strain has very low ATP levels, which constrains both the glycolytic and the ATPase flux. This will be further addressed in a later section.

**Table 1:** Specific growth, glucose uptake, and product synthesis rates and product yields of the tested strains under anaerobic growth with growth (top) and with growth arrest (bottom). Regarding the calculation of the ATPM and the ATPase rates see Materials and Methods. The means ± SD of *n* = 3 biologically independent samples are shown.

	WT	WT + IPTG	LC control	LC ATPase	MC control	MC ATPase	HC control	HC ATPase
**With growth**								
*µ* [h^−1^]	0.476 ± 0.004	0.449 ± 0.007	0.458 ± 0.004	0.376 ± 0.007	0.461 ± 0.002	0.197 ± 0.003	0.431 ± 0.004	0.063 ± 0.009
*r*_Glc_ [mmol/gDW/h]	13.48 ± 0.04	12.78 ± 0.48	14.05 ± 0.42	16.38 ± 0.56	15.02 ± 0.15	17.70 ± 0.47	12.93 ± 1.11	5.13 ± 0.51
*r*_Eth_ [mmol/gDW/h]	10.81 ± 0.14	9.99 ± 0.15	10.38 ± 0.24	13.23 ± 0.33	10.51 ± 0.19	15.63 ± 0.94	9.84 ± 0.52	2.29 ± 0.51
*r*_Ace_ [mmol/gDW/h]	11.90 ± 0.19	10.73 ± 0.30	10.74 ± 0.23	13.23 ± 0.22	10.93 ± 0.37	14.90 ± 0.59	9.89 ± 0.24	3.28 ± 0.39
*r*_For_ [mmol/gDW/h]	21.88 ± 0.37	19.54 ± 0.26	20.78 ± 1.07	26.99 ± 0.86	21.21 ± 0.30	30.24 ± 0.25	20.46 ± 0.66	4.69 ± 0.96
*r*_Lac_ [mmol/gDW/h]	0.83 ± 0.05	0.67 ± 0.05	0.89 ± 0.07	1.30 ± 0.11	0.98 ± 0.07	0.84 ± 0.34	0.83 ± 0.09	2.95 ± 0.13
*r*_Suc_ [mmol/gDW/h]	1.77 ± 0.02	1.72 ± 0.02	1.73 ± 0.10	1.80 ± 0.07	1.85 ± 0.05	1.66 ± 0.08	1.65 ± 0.09	0.45 ± 0.17
*r*_ATPM_ (calculated) [mmol/gDW/h]	4.41 ± 0.54	3.27 ± 0.31	3.30 ± 0.43	14.63 ± 1.04	3.61 ± 0.82	29.06 ± 0.67	2.67 ± 1.05	6.99 ± 0.73
*r*_ATPase_ (calculated) [mmol/gDW/h]				11.33		25.45		4.32
*Y*_BM_[gDW/g]	0.155 ± 0.005	0.160 ± 0.001	0.149 ± 0.002	0.104 ± 0.003	0.146 ± 0.001	0.050 ± 0.000	0.136 ± 0.002	0.065 ± 0.015
*Y*_Eth_ [mol/mol]	0.696 ± 0.024	0.716 ± 0.008	0.678 ± 0.009	0.770 ± 0.007	0.668 ± 0.015	0.857 ± 0.022	0.655 ± 0.013	0.460 ± 0.066
*Y*_Ace_ [mol/mol]	0.753 ± 0.016	0.774 ± 0.007	0.665 ± 0.008	0.790 ± 0.006	0.666 ± 0.010	0.840 ± 0.029	0.645 ± 0.010	0.650 ± 0.004
*Y*_For_ [mol/mol]	1.299 ± 0.031	1.309 ± 0.010	1.280 ± 0.014	1.542 ± 0.010	1.251 ± 0.019	1.572 ± 0.013	1.268 ± 0.013	0.899 ± 0.087
*Y*_Lac_ [mol/mol]	0.184 ± 0.004	0.166 ± 0.003	0.177 ± 0.005	0.161 ± 0.005	0.184 ± 0.003	0.143 ± 0.001	0.246 ± 0.000	0.570 ± 0.078
*Y*_Suc_[mol/mol]	0.125 ± 0.007	0.130 ± 0.005	0.118 ± 0.001	0.106 ± 0.003	0.124 ± 0.001	0.085 ± 0.001	0.125 ± 0.002	0.083 ± 0.017
**Growth arrest**								
*µ* [h^−1^]	~0	~0	~0	~0	~0	~0	~0	~0
*r*_Glc_ [mmol/gDW/h]	1.90 ± 0.11	2.02 ± 0.06	2.34 ± 0.34	8.96 ± 0.36	2.18 ± 0.06	10.46 ± 0.43	2.95 ± 0.09	8.10 ± 0.60
*r*_Eth_ [mmol/gDW/h]	1.43 ± 0.21	1.43 ± 0.04	1.47 ± 0.04	4.78 ± 0.35	1.43 ± 0.09	3.16 ± 0.40	1.34 ± 0.06	0.66 ± 0.14
*r*_Ace_ [mmol/gDW/h]	1.55 ± 0.07	1.50 ± 0.02	1.58 ± 0.04	4.69 ± 0.28	1.54 ± 0.03	2.56 ± 0.54	1.43 ± 0.06	0.60 ± 0.03
*r*_For_ [mmol/gDW/h]	2.23 ± 0.18	2.09 ± 0.03	2.10 ± 0.10	7.65 ± 0.47	2.04 ± 0.07	4.28 ± 0.82	2.17 ± 0.12	0.42 ± 0.03
*r*_Lac_ [mmol/gDW/h]	0.12 ± 0.02	0.22 ± 0.04	0.32 ± 0.12	5.71 ± 0.12	0.49 ± 0.19	11.31 ± 0.54	1.51 ± 0.10	13.78 ± 0.36
*r*_Suc_ [mmol/gDW/h]	0.75 ± 0.03	0.71 ± 0.02	0.95 ± 0.02	2.20 ± 0.08	0.89 ± 0.06	1.95 ± 0.01	0.94 ± 0.04	0.35 ± 0.04
*r*_ATPM_ (calculated) [mmol/gDW/h]	4.16 ± 0.27	4.29 ± 0.08	4.59 ± 0.23	18.62 ± 0.82	4.63 ± 0.12	19.31 ± 0.78	5.42 ± 0.14	15.63 ± 0.60
*r*_ATPase_ (calculated) [mmol/gDW/h]				14.33		14.68		10.21
*Y*_BM_ [gDW/g]	~0	~0	~0	~0	~0	~0	~0	~0
*Y*_Eth_ [mol/mol]	0.738 ± 0.009	0.703 ± 0.035	0.658 ± 0.011	0.497 ± 0.011	0.683 ± 0.010	0.284 ± 0.015	0.512 ± 0.032	0.076 ± 0.003
*Y*_Ace_ [mol/mol]	0.840 ± 0.021	0.758 ± 0.004	0.704 ± 0.015	0.467 ± 0.014	0.709 ± 0.028	0.259 ± 0.011	0.406 ± 0.024	0.092 ± 0.003
*Y*_For_ [mol/mol]	1.114 ± 0.039	0.993 ± 0.056	0.915 ± 0.055	0.645 ± 0.018	0.901 ± 0.058	0.305 ± 0.018	0.520 ± 0.036	0.059 ± 0.001
*Y*_Lac_ [mol/mol]	0.063 ± 0.006	0.084 ± 0.003	0.128 ± 0.023	0.728 ± 0.013	0.221 ± 0.036	1.177 ± 0.032	0.734 ± 0.023	1.740 ± 0.036
*Y*_Suc_ [mol/mol]	0.383 ± 0.026	0.341 ± 0.007	0.413 ± 0.003	0.227 ± 0.002	0.407 ± 0.006	0.155 ± 0.005	0.289 ± 0.008	0.043 ± 0.002

### Anaerobic cultivation under growth arrest

**Figure 2:**
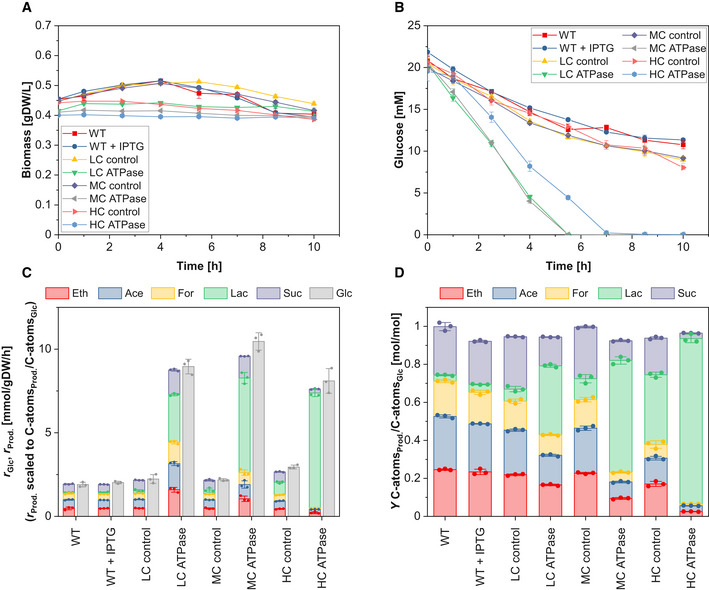
Anaerobic cultivation of the different strains under growth arrest Time course of biomass concentration.Time course of glucose concentration.Specific glucose (Glc) uptake rate and specific productivity for ethanol (Eth), acetate (Ace), formate (For), lactate (Lac), and succinate (Suc).Yield of Eth, Ace, For, Lac, and Suc. Data information: The reaction rates in (C) were calculated from the beginning of cultivation until the last sampling time point where glucose was still present in the medium. The means (A and B) and the means and individual data (C and D) for *n* = 3 biologically independent samples are shown. The error bars represent ± SD. Note: although no nitrogen source was present in the medium, some minor growth (especially of the control and wild type strains) remained (A), which is a known phenomenon within the first hours of cultivation after nitrogen depletion (Switzer *et al*, 2020). Source data are available online for this figure.

As the next step, we cultivated all strains anaerobically and arrested growth by transferring the cells to a medium without a nitrogen source. Such conditions are of particular interest for biotechnological applications (e.g., in two‐stage production processes (Burg *et al*, 2016; Klamt *et al*, 2018)). All ATPase strains showed high glycolytic and product exchange rates, while all control strains came to a metabolic halt and barely took up any glucose after 10 h of cultivation (Fig 2A–D; Table 1). Compared to their corresponding control strains, the LC ATPase strain had a 300% increased glucose uptake rate (8.96 mmol_Glc_/gDW/h), the MC ATPase strain a 380% increase (10.46 mmol_Glc_/gDW/h), and the HC ATPase still a 175% increase (8.10 mmol_Glc_/gDW/h). To our knowledge, the specific glucose uptake rate of the MC ATPase strain is the highest rate ever reported for growth‐arrested *E. coli* cells. The HC ATPase strain showed again the lowest glucose uptake rate among the three ATPase strains. While the WT and control strains metabolized most of the glucose‐carbon to ethanol, acetate, formate, and succinate, the amount of formed lactate increased with the expression strength of ATPase. The HC ATPase strain converted all glucose almost entirely to lactate (1.74 mol_Lac_/mol_Glc_) (Fig 2C and D; Table 1). As for the case of anaerobic growth, the overall ATPase flux in the HC ATPase strain was lower than in the other ATPase strains.

### Aerobic cultivations

**Figure EV1:**
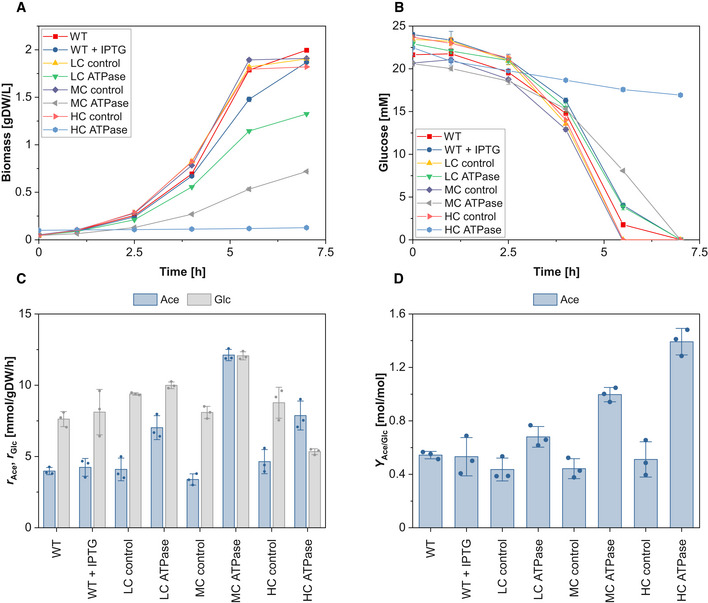
Aerobic growth of the different ATPase strains Time course of biomass concentration.Time course of glucose concentration.Specific glucose (Glc) uptake rate and specific productivity for acetate (Ace).Yield of Ace. Data information: The reaction rates in (C) were calculated for the exponential phase under assumption of quasi‐steady state. Because changes in glucose and acetate are rather small during cultivation of the HC ATPase strain, a higher initial biomass concentration of 0.1 gDW/l was used for this strain to get data that are more robust for calculating the metabolite exchange rates. The means (A and B) and the means and individual data (C and D) for *n* = 3 biologically independent samples are shown. The error bars represent ± SD. Source data are available online for this figure.

**Figure EV2:**
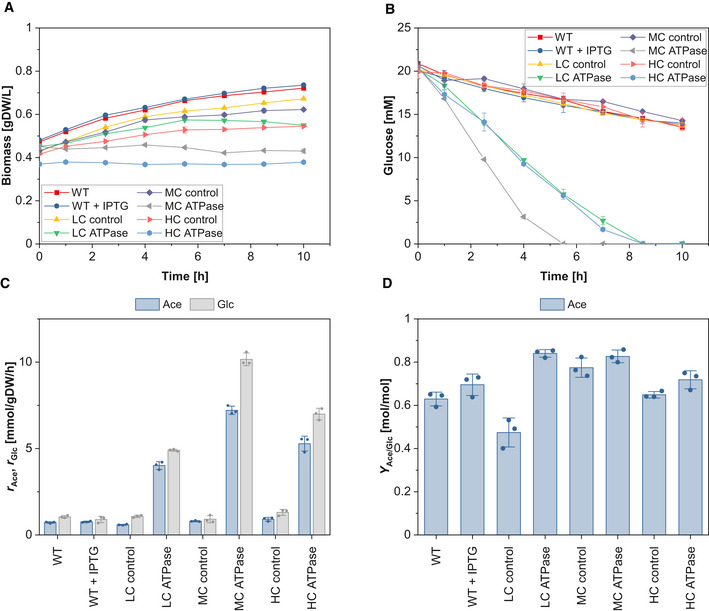
Aerobic cultivation of the different strains under growth arrest Time course of biomass concentration.Time course of glucose concentration.Specific glucose (Glc) uptake rate and specific productivity for acetate (Ace).Yield of Ace. Data information: The reaction rates in (C) were calculated from the beginning of cultivation until the last sampling time point where glucose was still present in the medium. The means (A and B) and the means and individual data (C and D) for *n* = 3 biologically independent samples are shown. The error bars represent ± SD. Note: although no nitrogen source was present in the medium, some minor growth (especially of the control and wild‐type strains) remained (A), which is a known phenomenon within the first hours of cultivation after nitrogen depletion (Switzer *et al*, 2020). Source data are available online for this figure.

We repeated the same experiments under aerobic conditions (Figs EV1A–D and EV2A–D, Appendix Tables S2 and S3). Essentially, the same trends as in the anaerobic cultivations could be observed. While all ATPase strains showed reduced growth rates and biomass yields compared to the control strains, the specific glucose uptake rates increased in the LC and MC ATPase strain by 6.5 and 49.2%, respectively, but dropped in the HC ATPase strain by 39.1% (Fig EV1C, Appendix Table S2). The yield of acetate, which is the major overflow byproduct of *E. coli* under aerobic conditions (Wolfe, 2005), increased with the expression strength of the ATPase genes. We also tested the behavior of the ATPase strains under aerobic conditions with growth arrest (Fig EV2A–D, Appendix Table S3). While the metabolism of the WT and control strains slowed down after some time (as under anaerobic conditions), all ATPase strains exhibited a very high metabolic activity. Again, a biphasic steady‐state response curve of the glucose uptake rate for increasing ATPase levels could be observed. The MC ATPase strain reached the highest glucose uptake rate (10.2 mmol_Glc_/gDW/h), which was more than 10‐fold higher than in the control strain (and even higher than in the WT with growth). Compared to the anaerobic case, we estimated considerably higher ATPase fluxes for the ATPase strains, which are possible due to the high ATP yield under aerobic conditions.

### Increasing ATP demand induces biphasic response of glucose uptake under all cultivation conditions

**Figure 3:**
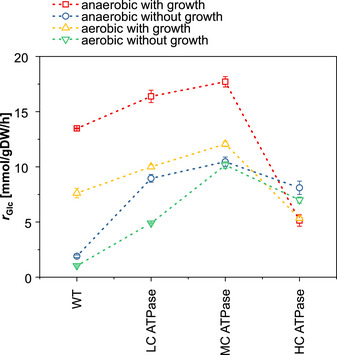
For all growth regimes tested, the glucose uptake rate shows a biphasic response curve to increasing ATPase levels The means for *n* = 3 biologically independent samples are shown. The error bars represent ± SD.

The data of the anaerobic and aerobic cultivations with and without growth consistently show a biphasic curve of the steady‐state glucose uptake rate as response to the increasing overexpression of the ATPase genes. As summarized in Fig 3, for all cultivations, the uptake rate increases from WT over LC to MC ATPase strain and then drops for HC ATPase strain, especially sharply for growing cells. This observation raises the key questions: what causes this biphasic steady‐state response, and why does *E. coli* not further increase (or at least maintain a high) glycolytic flux under maximal ATPase expression? For an in‐depth analysis of this phenomenon, we focused on the response of the different ATPase strains under anaerobic growth, where the highest specific glucose uptake rates for the LC and MC ATPase strain and the steepest drop of the uptake rate of the HC ATPase strain could be observed.

### Changes at the metabolome and proteome level for increasing ATPase abundance

**Figure EV3:**
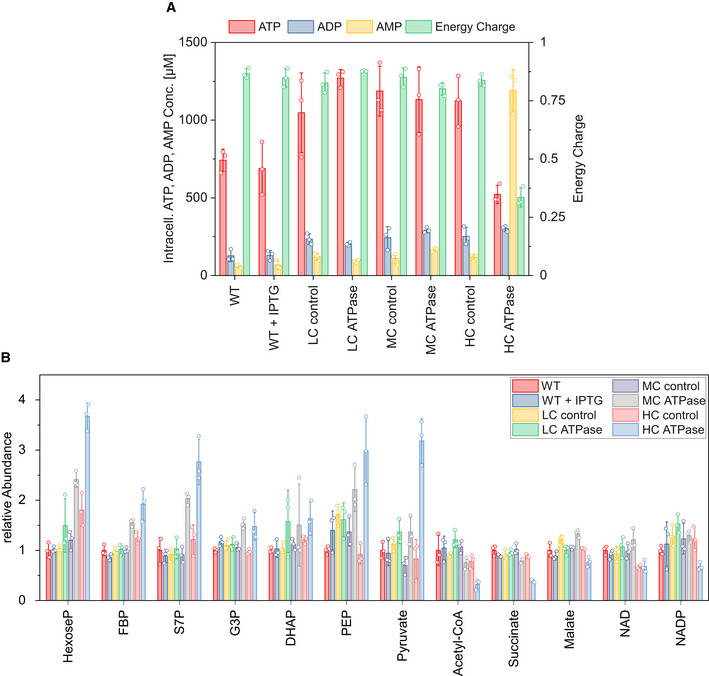
Intracellular metabolite concentrations of the different strains during anaerobic growth Absolute intracellular ATP, ADP, and AMP concentrations and energy charge.Relative intracellular metabolite concentrations from core metabolism. Data information: The means and individual data of *n* = 3 biologically independent samples are shown and the error bars represent ± SD.

**Figure EV4:**
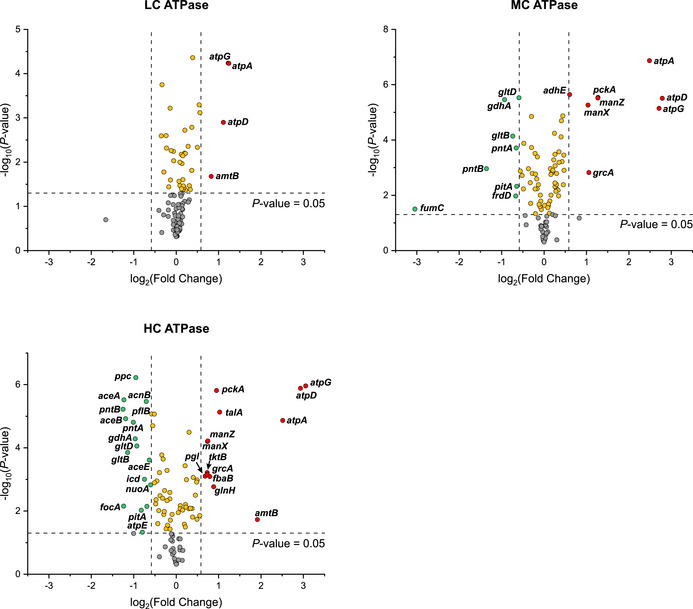
Changes on proteome level (only proteins from core metabolism are shown) of the three ATPase strains in regard to the corresponding control strains under anaerobic cultivation conditions with growth Significantly (*P* value < 0.05) upregulated (upregulation > 1.5‐fold) proteins are depicted in red, significantly (*P* value < 0.05) downregulated (downregulation > 0.33‐fold) proteins are depicted in green. Proteins with a significant down‐ or upregulation but below the thresholds of 1.5‐fold up‐ or 0.33‐fold downregulation are depicted in yellow, proteins with no significant change (*P* value > 0.05) are depicted in grey. The gene names of the corresponding proteins are given for significantly down‐ or upregulated proteins. *P* values were calculated for a two‐sample *t*‐test from *n* = 3 biologically independent samples.

For a comprehensive metabolic characterization of the different ATPase strains under anaerobic growth, we quantified the intracellular concentration of metabolites (Fig EV3A and B; Dataset EV1) and determined changes on proteome level (Fig EV4; Dataset EV1). ATP, ADP, and AMP concentrations remained relatively constant in the LC and MC ATPase strains compared to the control strains. Only the HC ATPase showed larger changes: the ATP level decreased by 56.0%, while the ADP level increased by 24.6% and the AMP level by almost 1,000% (Fig EV3A). These observations are also reflected by the adenylate energy charges, which are high in the WT and LC ATPase strain (0.87 and 0.88, respectively), only slightly lower in the MC ATPase strain (0.80), but significantly reduced in the HC ATPase strain (0.34) (Fig EV3A). This indicates that the LC and the MC, but not the HC ATPase strain, can compensate the higher ATP demand by the increased glycolytic flux.

The concentrations of several other intracellular metabolites changed also significantly. Generally, we observed that the concentration of metabolites involved in glycolysis (glucose‐6‐phosphate, fructose‐6‐phosphate (F6P), fructose‐1,6‐bisphosphate (FBP), dihydroxyacetone phosphate (DHAP), glyceraldehyde‐3‐phosphate (G3P), PEP, and pyruvate) consistently increased with expression strength of the ATPase genes, reaching highest values in the HC ATPase strain (Fig EV3B). Contrarily, the acetyl‐CoA concentration dropped in the HC ATPase strain (Fig EV3B).

In contrast to metabolite concentrations, at the proteome level, enzymes from the anaerobic core metabolism of *E. coli* were not as clearly up‐ or downregulated (Fig EV4; Dataset EV1). In particular, larger changes of glycolytic enzyme levels could not be seen; the abundance levels of these enzymes in the ATPase strains are all within the range of 60% and 150% of the respective levels in the control strains. This indicates that changes in the glycolytic fluxes are mainly induced by allosteric or substrate level regulation rather than by alteration of enzyme levels. As expected, the three subunits of the F_1_‐ATPase were more abundant with increasing copy number of the expression plasmids. The average abundances of the ATPase α‐, β‐, and γ‐subunits were +142% (LC ATPase), +549% (MC ATPase), and +708% (HC ATPase) in comparison to the WT strain (Fig EV4; Dataset EV1). Among the remaining enzymes from the core carbon metabolism, only the malate dehydrogenase (upregulated), PEP carboxykinase (PCK, upregulated), and the PEP carboxylase (PPC, downregulated) showed stronger changes in the MC and HC ATPase strains compared to their control strains. PCK and PPC are adjacent in the sense that PPC catalyzes the carboxylating reaction from PEP to oxaloacetate (with release of a phosphate molecule) and PCK the reaction from oxaloacetate to PEP thereby consuming ATP (see also Fig 5C). Under certain conditions, for example, low ATP or high PEP concentrations, the PCK reaction may also act in the reverse direction, and it seems that the PCK is used to replace the PPC to provide additional ATP. However, despite the fact that also the malate dehydrogenase is upregulated, the overall flux to succinate as final product of this pathway is still relatively low in the HC ATPase strain (Fig 1C and D; Table 1). Not as prominent but still noticeable was the downregulation of formate acteyltransferase (formerly pyruvate formate lyase; PFL) and of the formate transporter FocA in the HC ATPase strain. While the PFL and the FocA levels were not significantly affected in the LC and MC ATPase strains, in the HC ATPase strain, compared to its control, the levels dropped by 33% and 58%, respectively.

### What limits the glycolytic flux under high ATPase activity—a kinetic modeling approach

**Figure 4:**
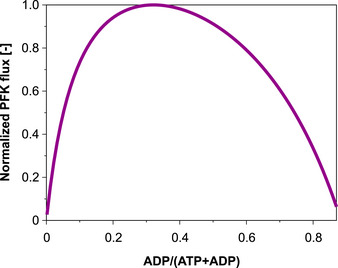
Simulation of the PFK rate with varying ADP/(ATP+ADP) ratios The PFK flux was simulated as single (isolated) reaction with the following fixed metabolite concentrations relevant for the kinetic rate law of the PFK (for the latter see Appendix Supplementary Text, section 1.3): PEP: 0.27 µmol/gDW; F6P: 0.91 µmol/gDW; FBP: 9.74 µmol/gDW; total concentration ATP+ADP: 2.67 µmol/gDW.

With the experimental findings and data at hand, we sought to find a mechanistic explanation for the inability of the HC ATPase strain to sustain a high glycolytic flux, as in the LC and MC ATPase strain, to compensate the high ATP demand. Given the low energy charge in the HC ATPase strain and the increased ratio between hexose phosphates and FBP, we hypothesized that the kinetics of the phosphofructokinase (PFK), reaction converting F6P to FBP under consumption of (Mg)ATP, might cause the biphasic behavior. This reaction is considered as the committing step of glycolysis and is a major point of regulation in *E.coli* (Fenton & Reinhart, 2009). In *E*. *coli*, there are two PFKs (PFK1 (encoded in *pfkA*) and PFK2 (*pfkB*)); however, more than 90% of the PFK activity in *E. coli* can be attributed to PFK1 (Kotlarz *et al*, 1975). This enzyme is allosterically inhibited by phosphoenolpyruvate (PEP) and has a dual dependency on (Mg)ADP and (Mg)ATP (Blangy *et al*, 1968). On the one hand, high concentrations of the substrate (Mg)ATP are required to obtain a high PFK flux. On the other hand, ADP (as well as GDP) is known to allosterically activate the reaction (Peskov *et al*, 2008). Indeed, using a kinetic rate law based on convenience kinetics (Liebermeister & Klipp, 2006) and including the effect of the allosteric regulators leads to a biphasic curve when increasing the ADP/(ATP + ADP) ratio (Fig 4; cf. also Ref. Peskov *et al*, 2008). For high ATP levels (low ADP/(ADP + ATP) ratio), ADP as activator is limiting. With increasing ADP levels induced by higher ATPase activity, the PFK rate increases as well, but only to an optimal point beyond which the concentration of the co‐substrate ATP becomes limiting. As second step of the glycolysis, the PFK reaction kinetics could thus be the cause for the observed biphasic response of the glucose uptake rate and the low glucose consumption rate in the HC ATPase strain could be a consequence of the low ATP concentration (high ADP/(ATP + ADP) ratio) in this strain.

**Figure 5:**
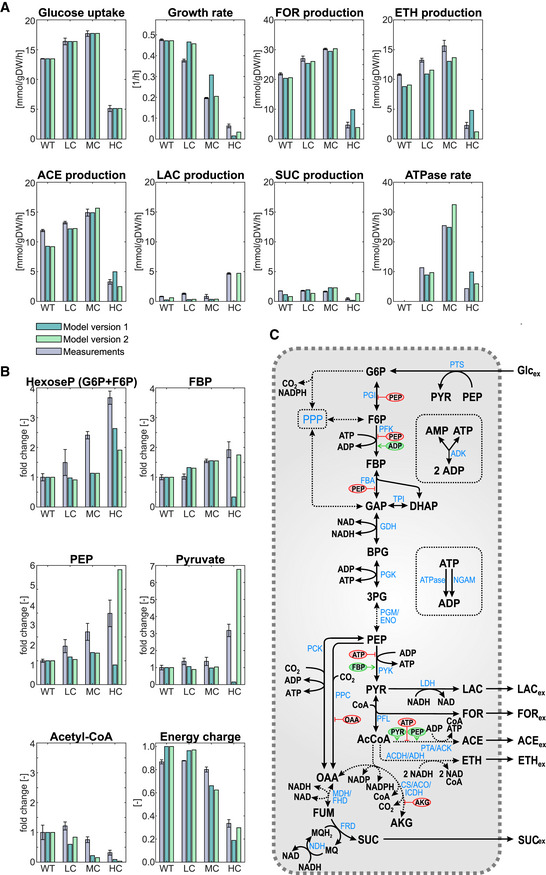
Comparison of the simulations of the kinetic model version 1 and version 2 with experimental data of the different strains under anaerobic growth conditions Comparison of the simulations of the kinetic model version 1 and version 2 with the measured exchange fluxes.Comparison of the simulations of the kinetic model version 1 and version 2 with the measured metabolite concentrations.Metabolic map containing most relevant reactions (blue) and metabolites (black) of *E. coli* under anaerobic conditions. Dashed arrows indicate (lumped) reactions with several enzymes involved. Allosteric regulations of the respective reactions are marked in red (inhibition) or green (activation). The kinetic model (versions 1 and 2) covers almost all of the shown reactions and regulations; a map directly related to the model is shown in Appendix Fig S1. Abbreviations of metabolites and reaction names: Glc_ex_: external glucose (substrate); G6P: d‐glucose‐6‐phosphate; F6P: d‐fructose‐6‐phosphate; FBP: fructose‐1,6‐bisphosphate; GAP: d‐glyceraldehyde‐3‐phosphate; DHAP: dihydroxyacetone phosphate; BPG: 1,3‐bisphospho‐d‐glycerate; 3PG: 3‐phosphoglycerate; PEP: phosphoenol‐pyruvate; PYR: pyruvate; AcCoA: acetyl coenzyme A; CoA: coenzyme A; AKG: α‐ketoglutarate; OAA: oxaloacetate; FUM: fumarate; SUC: succinate; FOR: formate; LAC: lactate; ACE: acetate; ETH: ethanol; ATP: adenosine triphosphate; ADP: adenosine diphosphate; NAD: oxidized nicotinamide adenine dinucleotide; NADP: oxidized nicotinamide adenine dinucleotide phosphate; NADH: reduced nicotinamide adenine dinucleotide; NADPH: reduced nicotinamide adenine dinucleotide phosphate; CO_2_: carbon dioxide; MQH_2_: menaquinol; MQ: menaquinone. PTS: phoshotransferase system; PGI: glucose‐6‐phosphate isomerase; PFK: phosphofructokinase; FBA: fructose‐bisphosphate aldolase; TPI: triose‐phosphate isomerase; GHD: glyceraldehyde‐3‐phosphate dehydrogenase; PGK: phosphoglycerate kinase; PGM: phosphoglycerate mutase; ENO: enolase; PYK: pyruvate kinase; PFL: pyruvate formate‐lyase (also known as formate acteyltransferase); LDH: lactate dehydrogenase; PTA: phosphate acetyltransferase; ACK: acetate kinase; ACDH: acetaldehyde‐CoA dehydrogenase; ADH: alcohol dehydrogenase; PCK: phosphoenolpyruvate carboxykinase; PPC: phosphoenolpyruvate carboxylase; CS: citrate synthase; ACO: aconitate hydratase A/B; ICDH: isocitrate dehydrogenase; MDH: malate dehydrogenase; FHD: fumarase; FRD: fumarate reductase; NDH: NADH dehydrogenase; ADK: adenylate kinase; NGAM: ATP consumption for non‐growth‐associated maintenance; ATPase: ATP hydrolysis by F_1_‐ATPase in the ATPase strains. Data information: For the measurements, the means for *n* = 3 biologically independent samples are shown (A and B) and the error bars represent ± SD.

However, it is not clear whether the biphasic response of the isolated PFK reaction in Fig 4 (with fixed concentrations of PEP, FBP, F6P) translates into a biphasic response of the glycolytic flux when the entire central metabolism with all its interactions and feedbacks is taken into account. We therefore constructed a kinetic model of the central fermentative metabolism of *E. coli* for anaerobic growth on glucose (see detailed description in Appendix Supplementary Text; section 1). The model comprises 33 metabolites and 28 reactions and, as shown in Fig 5C, accounts for the glycolysis, anaplerotic reactions, relevant parts of the TCA cycle, the major fermentative pathways, a growth reaction, a reaction for non‐growth‐associated ATP maintenance (NGAM) demand, and, finally, a reaction for simulating ATP hydrolysis by the ATPase. The latter reaction depends on the ATPase overexpression level. The model also accounts for allosteric regulation of the involved enzymes. As described in detail in the Appendix Supplementary Text, for the first version of the model, we fitted the unknown parameters of the model to the measured growth rate, the substrate uptake rate, and the product exchange fluxes of the wild type and the different ATPase strains (given in Table 1) as well as to measured metabolite levels in these strains (see above and Dataset EV1). Since the proteomic data indicated only minor or moderate changes in the levels of relevant metabolic enzymes, we used, as an approximation, constant $v_{\max}$ values (i.e., constant enzymes concentrations) in all four strains (WT as well as LC, MC, and HC ATPase strains) in the kinetic model simulations. We were able to find a parametrization that gave a reasonable fit with the experimental data (Fig 5A and B). In particular, the fitted model could reproduce the observed biphasic response of the glucose uptake along the four strains (first plot in Fig 5A) demonstrating that the known metabolic and regulatory interactions contained in the kinetic model are sufficient to generate this behavior.

**Figure 6:**
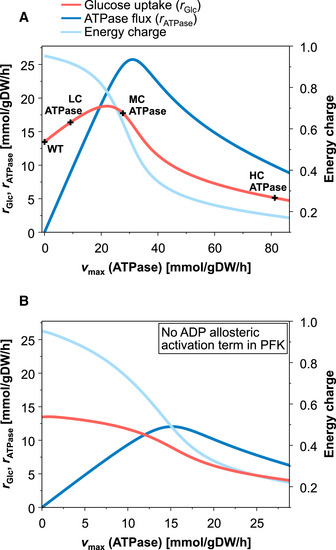
Simulation of the steady‐state response curves of glucose uptake rate, energy charge, and ATPase flux under anaerobic growth for increasing ATPase activities Simulations with the standard kinetic model (version 1). The measured glucose uptake rates of the different strains are indicated (cf. with Fig 3).Simulations with the kinetic model (version 1) as in (A), but without ADP activation term in the PFK kinetics.

Next, we used the model to simulate the steady‐state response curves of glucose uptake rate, energy charge, and ATPase flux when increasing the ATPase level (represented by $v_{\max}$ of the ATPase reaction) continuously from 0 (WT) to a maximal value of 85 mmol/gDW/h (Fig 6A). Starting with a low ATPase level, the ATPase flux increases causing a higher ADP concentration (lower energy charge) which, as discussed in Fig 4, enhances the PFK and thereby the glycolytic flux. However, further increasing the abundance of ATPase beyond a critical point reduces the ATP concentration, now limiting the PFK flux and thus the glucose uptake rate (Fig 6A). These model simulations also confirmed that the rate of ATP hydrolysis by the ATPase is lowest in the HC ATPase strain, despite the fact that it has the highest ATPase abundance (i.e., the highest $v_{max,\;ATPase}$). Available ATP is rapidly consumed by the large amounts of ATPase in the HC ATPase strain, which keeps the concentration of ATP at a very low level limiting in turn both the PFK as well as the ATPase flux. To further test our hypothesis regarding the PFK mechanism and its effect on the glucose uptake under increasing ATP demand, we utilized the model to evaluate how the steady‐state response curves in Fig 6A change if we remove the allosteric regulation of PFK by ADP (the respective ADP‐dependent term in the kinetic rate law was fixed (clamped) to its wild‐type value in all simulations). Indeed, the biphasic response of the glucose uptake disappears and a monotonic decrease in glucose uptake, upon enhanced ATP demand, can be seen (Fig 6B).

Taken together, our modeling results strongly support our hypothesis that the dual dependency of the PFK on ADP and ATP causes the observed biphasic response in the glycolytic flux. As a consequence, the PFK mechanism enables the cell to buffer moderate ATPase activities (and to compensate moderate increases in ATP demand) by an instantaneous adjustment of the glycolytic flux, but it collapses under very high ATP demand as in the HC ATPase strain.

While the kinetic model (version 1) reproduced most experimental data in the different strains reasonably well, especially the exchange rates (Fig 5A), some deviations can be noted in the metabolite concentrations. The measured accumulation of hexose phosphates (glucose‐6‐phosphate, F6P) is correctly reflected by the model, again indicating that the PFK activity is the limiting factor in the HC ATPase strain. However, the measured high concentrations of the glycolytic metabolites FBP, PEP, and pyruvate in the HC ATPase strain are not captured in the simulation results. For example, the measured concentrations of FBP and pyruvate increased markedly in the HC ATPase strain, but the opposite behavior (strong decrease) was displayed by the model. Even after several rounds of parameter fitting, we could not find a parametrization that leads to a better reproduction of the qualitative trends of the metabolomics data. These discrepancies between data and model simulations suggested that there are missing regulatory elements in our kinetic model, and we, therefore, introduced two major changes (model version 2; Appendix Supplementary Text). As a first change, because of (a) the low concentration of acetyl‐CoA, (b) the accumulation of pyruvate, (c) the increased lactate yield, and (d) the observed reduced PFL levels in the HC ATPase strain, we introduced a term in the kinetics of the PFL reaction, which ensures a lower abundance of PFL (and thus a decreased $v_{\max}$) under low ATP concentrations. As a possible mechanistic explanation, we hypothesized that, at low energy charges, the large and costly PFL (consisting of 759 amino acids) is replaced by cheaper pathways (e.g., via lactate fermentation), although this may partially reduce the ATP yield (see also Discussion). However, even with introduction of a downregulation of the PFL under low ADP concentrations, the model was still not able to reflect the high concentration levels of PEP and FBP in the HC ATPase strain. In fact, with (i) the observed high concentrations of PEP and ADP (the substrates for the pyruvate kinase (PYK) reaction), (ii) the high FBP level (allosteric activator of pyruvate kinase), and (iii) the highly negative standard Gibbs free energy change of this reaction (Δ_r_ *G*°’ = −21.78 kJ/mol) (Park *et al*, 2016), one would expect a higher PYK flux and thus a decrease of the PEP level, which contradicts the measured high PEP concentration in the HC ATPase strain (Fig 5B). As a second change in the model, we therefore introduced a term inhibiting PYK (flux) under high pyruvate concentrations. Although such an allosteric inhibition of PYK by pyruvate is not known, it has been reported that alanine, which is directly produced from pyruvate, may act as inhibitor for PYK (Taber *et al*, 1998), and we observed higher concentrations of alanine in all ATPase strains (Dataset EV1).

With these two changes, the resulting model version 2 was now able to reproduce the qualitative trends in the fluxes and metabolite concentrations in all three ATPase strains (Fig 5A and B). With this, we can summarize our understanding for the observed phenomena as follows: increasing the ATPase activity reduces the growth rate in all strains and elevates the glycolytic flux in the LC and MC ATPase strains due to (moderately) increased ADP levels, which enhances the activity of PFK. However, in the HC ATPase strain, there is a sharp decrease of ATP, a substrate of the PFK, which now limits the PFK and thereby the glycolytic flux. As consequence of low ATP levels, the PFL abundance is reduced, which lowers the flux from pyruvate to acetyl‐CoA. Thus, pyruvate accumulates, which leads to higher lactate production rates and inhibits (probably indirectly) PYK activity. Consequently, the levels of PEP increase further, which further slows down the PFK flux due to negative inhibition, and thus, the concentrations of hexose phosphates increase. Finally, high PEP concentrations propagate also upward to other glycolytic intermediates (DHAP, G3P, FBP). Since PEP also inhibits fructose‐bisphosphate aldolase, this further contributes to FBP accumulation. We note that the steady‐state response curves shown for model version 1 in Fig 6 are not affected by the model changes and look very similar for model version 2.

### Using Monte Carlo sampling of kinetic parameters to assess the robustness of model predictions

The kinetic model (version 2) of *E coli*'s central metabolism constructed in the previous section is relatively large and comprises more than 100 unknown parameters. To assess the robustness of model predictions with respect to kinetic parameters, we employed Monte Carlo analysis as previously described in (Murabito *et al*, 2014). Briefly, we used this method to sample Michaelis–Menten parameters over two orders of magnitude while preserving the metabolic steady state to which the original parameters were fitted. For each sampled set of kinetic parameters, systems properties can be computed. Here, we focused on the flux control coefficients (FCCs), which are known from metabolic control analysis and quantify the relative change of a steady‐state metabolic flux when changing the enzyme level, that is, when changing the $v_{\max}$, of other reactions (Sauro, 2019). The resulting distributions of FCCs obtained from the Monte Carlo analysis allow us to assess the control properties with different (but consistent) parametrizations and consequently to analyze the uncertainty of these global characteristics in our deterministic model. For details of the method, we refer to (Murabito *et al*, 2014) and the Appendix Supplementary Text (section 4).

For the wild‐type metabolic steady state, we found predominantly narrow distributions of FCCs, indicating a low sensitivity of these FCCs against parameter variations (Appendix Fig S3). FCCs with broader distributions were typically sign‐dominant. That is, while the numerical value of the FCCs varies as a function of kinetic parameters, the sign of the FCCs remains either positive or negative, respectively, indicating robust qualitative control properties and hence robust predictions based on the FCCs. Importantly, the FCC distribution of the NGAM reaction on PTS was narrow with predominantly positive sign (>95% of sampled instances), confirming that the increase of glucose uptake in the wild type as response to higher ATP demand is a robust feature of the model.

Using instead the HC ATPase strain (with its high ATP demand) as reference metabolic (steady) state, most FCCs still show narrow distributions, but the fraction of enzymes with a broader distribution of FCCs increased (Appendix Fig S4). The moderately increased sensitivity to parameter variations can be interpreted as reduced robustness of the HC ATPase metabolic state. In particular, the enzymes PYK, PFL, and PFK exhibited broad but sign‐dominant (positive) distributions of their respective FCCs on PTS and glycolytic flux, indicating that the sign (positive control) of these enzymes on the glycolytic flux in this metabolic state is largely independent of the choice of kinetic parameters. Furthermore, the FCCs of the NGAM and ATPase reactions on PTS and glycolytic flux are here predominantly negative. Together with the predominantly positive FCCs of NGAM on PTS and glycolytic flux in the wild type, this confirms the bi‐phasic response of the glycolytic flux to increased ATP demand as a robust feature of the model. The full results of the Monte Carlo analysis are shown and discussed in Appendix Supplementary Text.

### Using model predictions to obtain higher glycolytic fluxes in the HC ATPase strain

Motivated by the Monte Carlo analysis from the previous section, in a final step, we aimed to use the kinetic model (version 2) to make experimentally testable predictions and, in this way, to further verify our reasoning of the low glycolytic flux in the HC ATPase strain. We hypothesized that we could enhance the glycolytic flux by overexpressing genes of enzymes catalyzing reactions with the highest metabolic control. We computed the FCCs of all glycolytic reactions in the kinetic model. Since FCCs are valid only for small changes in the enzyme level, we also computed the resulting steady‐state glycolytic flux in the model when doubling the enzyme level, corresponding to doubled $v_{\max}$ values of the respective reactions. The results can be found in the Appendix Supplementary Text (section 2.3 and Fig S2). Consistent with our previous reasoning on the experimental findings and consistent with the results from the Monte Carlo analysis, we found that the PFL and the PFK have the highest control on the glycolytic flux in the HC ATPase strain. We therefore overexpressed the genes of these two enzymes to test whether they indeed represent bottlenecks. We constructed four variants of the HC ATPase strain: one overexpressing the PFK‐encoding gene *pfkA*, one overexpressing the PFL‐encoding gene *pflB,* one overexpressing both genes, and one overexpressing the phosphoglycerate kinase (PGK) encoding gene *pgk* (in all cases additionally to the *atpAGD* operon on the high copy plasmid). Overexpression of *pgk* was chosen as a control since the model predicted an FCC close to zero for the PGK reaction. Thus, in contrast to the other three strains, PGK overexpression should not have a major influence on the glycolytic flux of the HC ATPase strain. The four variants were grown under anaerobic conditions and compared to the HC ATPase strain.

**Figure 7:**
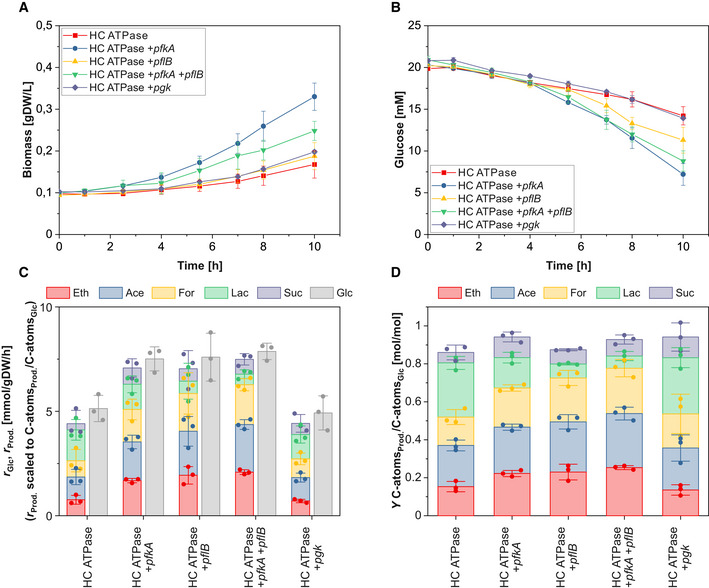
Anaerobic growth of the five HC ATPase strain variants Time course of biomass concentration.Time course of glucose concentration.Specific glucose (Glc) uptake rate and specific productivity for ethanol (Eth), acetate (Ace), formate (For), lactate (Lac), and succinate (Suc).Yield of Eth, Ace, For, Lac, and Suc. Data information: The reaction rates in (C) were calculated for the exponential phase under assumption of quasi‐steady state. The means (A and B) and the means and individual data (C and D) for *n* = 3 biologically independent samples are shown. The error bars represent ± SD. Source data are available online for this figure.

As shown in Fig 7A–D and Table 2, the overexpression of *pfkA* and *pflB* indeed had significant effects on the specific glucose uptake rate as well as on the composition of the fermentation products, while we observed no significant changes for overexpression of *pgk* compared to the HC ATPase strain. In particular, the glucose uptake rate increased by 46.4% (*pfkA*), 48.0% (*pflB*), and 53.4% (*pfkA*+*pflB*) compared to the HC ATPase strain and remained almost constant for the *pgk* overexpressing strain. As expected and qualitatively predicted by the model, due to the enhancement of the PFL flux, the lactate yield is largely reduced in the *pflB* and *pfkA*+*pflB* overexpressing strains. The lactate yield dropped also in the *pfkA* overexpressing strain, although to a lesser extent. Here, it is likely that the higher overall glycolytic flux, enabled by higher PFK activity, increases ATP supply and thus leads to less downregulation of PFL. Therefore, more carbon is redirected to acetate, ethanol, and formate and less to lactate also in the *pfkA* overexpressing strain. We also observed that the growth rate was increased, at most in the two *pfkA* overexpressing strains and less in the strain overproducing (only) PFL. This might again be related to the relative high protein costs of PFL.

**Table 2:** Growth rates, specific glucose uptake and product synthesis rates, and product yields of the five HC ATPase variants during anaerobic growth. For the calculations of the ATPM and the ATPase rates, see Methods. The means ± SD of *n* = 3 biologically independent samples are shown. *P* values for a two‐sample *t*‐test are given with respect to the HC ATPase strain. *P* > 0.05 was considered as not significant (n.s.).

	HC ATPase	HC ATPase + *pfkA*	HC ATPase + *pflB*	HC ATPase + *pfkA* + *pflB*	HC ATPase + *pgk*
*µ* [h^−1^]	0.063 ± 0.009	0.134 ± 0.013 (*P* = 0.003)	0.079 ± 0.009 (*P* = 0.161, n.s.)	0.098 ± 0.006 (*P* = 0.011)	0.074 ± 0.003 (*P* = 0.203, n.s.)
*r*_Glc_ [mmol/gDW/h]	5.13 ± 0.51	7.51 ± 0.47 (*P* = 0.009)	7.59 ± 0.94 (*P* = 0.031)	7.87 ± 0.32 (*P* = 0.003)	4.92 ± 0.67 (*P* = 0.743, n.s.)
*r*_Eth_ [mmol/gDW/h]	2.29 ± 0.51	5.05 ± 0.26 (*P* = 0.002)	5.81 ± 1.02 (*P* = 0.012)	6.26 ± 0.28 (*P* < 0.001)	2.12 ± 0.21 (*P* = 0.686, n.s.)
*r*_Ace_ [mmol/gDW/h]	3.28 ± 0.39	5.56 ± 0.54 (*P* = 0.009)	6.32 ± 0.79 (*P* = 0.008)	6.84 ± 0.29 (*P* < 0.001)	3.36 ± 0.35 (*P* = 0.835, n.s.)
*r*_For_ [mmol/gDW/h]	4.69 ± 0.96	9.32 ± 0.84 (*P* = 0.007)	10.88 ± 1.38 (*P* = 0.006)	11.44 ± 0.33 (*P* < 0.001)	5.38 ± 0.32 (*P* = 0.385, n.s.)
*r*_Lac_ [mmol/gDW/h]	2.95 ± 0.13	2.41 ± 0.15 (*P* = 0.019)	1.18 ± 0.22 (*P* < 0.001)	1.01 ± 0.55 (*P* = 0.008)	2.33 ± 0.22 (*P* = 0.027)
*r*_Suc_ [mmol/gDW/h]	0.45 ± 0.17	1.17 ± 0.05 (*P* = 0.004)	0.89 ± 0.03 (*P* = 0.024)	1.07 ± 0.08 (*P* = 0.010)	0.80 ± 0.04 (*P* = 0.047)
*r*_ATPM_ (calculated) [mmol/gDW/h]	6.99 ± 0.73	9.26 ± 0.23 (*P* = 0.014)	12.67 ± 2.02 (*P* = 0.020)	12.97 ± 0.39 (*P* < 0.001)	5.47 ± 0.34 (*P* = 0.055, n.s.)
*r*_ATPase_ (calculated) [mmol/gDW/h]	4.32	6.59	10.00	10.30	2.80
*Y*_BM_ [gDW/g]	0.065 ± 0.015	0.099 ± 0.005 (*P* = 0.038)	0.053 ± 0.009 (*P* = 0.382, n.s.)	0.068 ± 0.004 (*P* = 0.794, n.s.)	0.075 ± 0.005 (*P* = 0.404, n.s.)
*Y*_Eth_ [mol/mol]	0.460 ± 0.066	0.667 ± 0.040 (*P* = 0.019)	0.690 ± 0.101 (*P* = 0.055, n.s.)	0.761 ± 0.026 (*P* = 0.004)	0.407 ± 0.067 (*P* = 0.471, n.s.)
*Y*_Ace_ [mol/mol]	0.650 ± 0.004	0.732 ± 0.030 (*P* = 0.018)	0.794 ± 0.025 (*P* = 0.001)	0.854 ± 0.057 (*P* = 0.008)	0.663 ± 0.105 (*P* = 0.873, n.s.)
*Y*_For_ [mol/mol]	0.899 ± 0.087	1.239 ± 0.039 (*P* = 0.007)	1.387 ± 0.016 (*P* = 0.001)	1.434 ± 0.057 (*P* = 0.002)	1.078 ± 0.170 (*P* = 0.254, n.s.)
*Y*_Lac_ [mol/mol]	0.570 ± 0.078	0.320 ± 0.039 (*P* = 0.015)	0.147 ± 0.062 (*P* = 0.004)	0.128 ± 0.070 (*P* = 0.004)	0.592 ± 0.084 (*P* = 0.796, n.s.)
*Y*_Suc_ [mol/mol]	0.083 ± 0.017	0.163 ± 0.003 (*P* = 0.003)	0.113 ± 0.011 (*P* = 0.103, n.s.)	0.131 ± 0.002 (*P* = 0.016)	0.163 ± 0.033 (*P* = 0.037)

Although the glucose uptake rates of the *pfkA* and *pflB* overexpression strains were still below WT level, the data in Table 2 confirm that PFK and PFL are indeed limiting (bottleneck) reactions for the glycolytic rate in the HC ATPase strain, which can be partly overcome by overexpression of the corresponding genes.

## Discussion

In this study, we systematically analyzed the consequences of increasing ATP demand on the physiology of the *E. coli* wild‐type strain MG1655 under various conditions (aerobic/anaerobic, with/without cell growth). On the one hand, this study was curiosity‐driven to explore maximal physiological capabilities of *E. coli* and to investigate how this bacterium responds to situations of high ATP demand (which may be relevant under challenging environmental conditions, e.g., under osmotic, acidic, or toxin‐induced stress). On the other hand, this study aimed to deliver new insights toward the use and potential of enforced ATP wasting as a metabolic engineering strategy. We collected a comprehensive dataset on metabolic fluxes, metabolite concentrations, and protein abundances. In order to integrate these data with our current knowledge of the complex metabolism and its regulation in *E. coli*, we constructed a kinetic model that enabled us to modulate the ATP maintenance reaction and thus simulating different levels of ATP wasting in the cells.

The key findings of this study can be summarized as follows. First, in all conditions tested, there is a biphasic steady‐state response curve of the glucose uptake rate with respect to increasing ATPase activity. There is a maximum uptake rate at a medium ATPase level, and the glucose uptake rate drops markedly beyond this level. Second, the model indicates that the PFK reaction with its dual dependency on ADP/ATP causes this biphasic behavior. The PFK mechanism is known to buffer increased ATP demands by a higher glycolytic flux (due to elevated ADP levels) but, as shown herein, it collapses under high ATP demands with low ATP concentrations. Third, the metabolomics data under anaerobic conditions show an increasing accumulation of glycolytic metabolites reaching highest values at maximal ATPase level. This behavior cannot be explained with current knowledge, and we postulate that there are unknown regulatory mechanisms for PYK (presumably allosteric regulation by pyruvate or alanine) and PFL (enzyme‐level regulation). Finally, we validated the model predictions that PFK and PFL are rate‐limiting in the HC ATPase strain and found that overexpressing the genes of these enzymes indeed restores some of the glycolytic capacity.

The kinetic model played an important role in this study to identify and analyze potential mechanisms in the metabolism of *E. coli* that led to the observed phenotypes under high ATP demand. It has to be noted that the model is relatively large and comprises more than 100 unknown parameters, many of which will not be uniquely identifiable, despite fitting the model against a considerable set of data. However, the model is based on established biological knowledge of *E. coli*’s central metabolism, it is able to reproduce measurements of the different strains reasonably well and it gave predictions that could be successfully verified. Moreover, the results of the Monte Carlo sampling of kinetic parameters showed that key properties of the kinetic model and its predictions are robust over a wide range of parameter variations. Hence, despite potential parameter identifiability issues, the model could demonstrate its predictive power and thus represents a solid and plausible basis that supports our hypotheses and explains major findings of this study. However, as is true for every model, we can neither prove its correctness nor that other models with alternative mechanisms may exist that reproduce the observed phenomena equally well.

Our results are in several aspects consistent with current knowledge on the physiology of energy metabolism in *E. coli* but, at the same time, indicate gaps in our understanding. For example, the increasing levels of FBP in the LC and MC ATPase strains are consistent with the linear correlation between glycolytic flux and FBP concentration that has been shown for *E*. *coli* under various conditions and carbon sources (Kotte *et al*, 2010; Kochanowski *et al*, 2013). Based on these earlier findings, it was proposed that FBP—presumably indirectly via the transcription factor Cra (Bley Folly *et al*, 2018)—acts as a general flux‐sensing metabolite for *E. coli*. However, among all strains, the HC ATPase strain had the highest level of intracellular FBP but by far the lowest glycolytic flux. Thus, the generalization of FBP being a flux‐sensor might not to be true for extreme metabolic perturbations as in the HC ATPase strain. Our data also confirm previous work on overflow metabolism and proteome allocation phenomena in *E. coli*. In particular, the observed increase in acetate formation under aerobic conditions in the LC and MC ATPase strains is likely a consequence of proteome reallocation from respiratory pathways (with high ATP yield but also high protein costs) toward overflow metabolism. This consequently results in lower ATP yields but enables higher glycolytic fluxes (and thereby higher total ATP synthesis rates), due to reduced protein costs (Chen & Nielsen, 2019).

The observed downregulation of PFL in the HC ATPase strain under anaerobic conditions could be a strategy to optimize proteome allocation, here as response to low ATP concentrations. Again, lactate excretion (yielding 2 mol ATP per mol of glucose) seems disadvantageous compared to the PFL reaction in combination with the formation of acetate, ethanol, and formate resulting in an ATP yield of 2.5 mol ATP per mol of glucose. However, since PFL is a rather large enzyme (759 amino acids vs. 343 amino acids in the average essential protein in *E. coli* (Gong *et al*, 2008)), under the low ATP levels in the HC ATPase strain, it could be more cost‐efficient for *E. coli* (in terms of ATP demand) to use the lactate pathway. The lactate dehydrogenase appears to be constitutively available under anaerobic conditions, and the pathway becomes active with rising pyruvate concentrations. Under growth‐arrested conditions with a limited nitrogen source, which further limits the proteome pool, the effect is even more drastic. Here, almost the entire carbon is converted into lactate in the HC ATPase strain (yield of 1.74 mol_Lac_/mol_Glc_, Fig 2; Table 1), and increased lactate yields are also observed in the LC and MC ATPase strains. Another interesting insight from the proteomic data in the HC ATPase strain is the replacement of PPC with PCK. Due to the low ATP level, PCK may run in the direction of oxaloacetate and ATP synthesis, thus increasing the ATP yield compared to the sole use of PPC. While overexpression of the PCK genes has been used to increase the ATP yield in *E. coli* strains (Chao & Liao, 1993; Kwon *et al*, 2008; Zhang *et al*, 2009; Aslan *et al*, 2017; Kyselova *et al*, 2018), we are not aware of a previous report showing that *E. coli* naturally switches to PCK to enhance ATP supply. Identifying the regulatory mechanisms that enable this switch is an interesting aspect of future work.

When analyzing proteome (re)allocation, we also need to consider the effect of the ATPase overproduction on the proteome pool. Especially in the HC ATPase strain, the expression of the ATPase genes consumes cellular resources such as amino acids and may thereby reduce the overall capacity to synthesize other proteins, including metabolic enzymes. Hence, in addition to the discussed low ATP concentrations, changes in the proteome composition (such as the reduced PFL abundance) may also be induced by the synthesis costs of the ATPase subunits. However, there are several evidences that the *activity* of the ATPase is the dominant factor, rather than the *costs* of its synthesis. First of all, compared to the WT (where the three components of the F_1_‐subunit are used to build the F_O_F_1_‐ATP synthase), the proteomic data indicated a moderate averaged 8.1‐fold increase of the three F_1_‐ATPase components in the HC ATPase strain, which is still a comparably small fraction of the overall proteome pool. Moreover, we see a 6.5‐fold increase of ATPase abundance already in the MC ATPase strain; hence, there is effectively only a 25% increase of the ATPase level in the HC strain compared to the MC strain. With this relatively small change, it appears unlikely that the drastic change in the glycolytic flux and ATP levels between these two strains is mainly caused by a reduction of available resources for protein synthesis. Furthermore, except for the mentioned major changes, other enzymes of central metabolic steps, especially in the glycolysis, show a relatively constant level in the proteomic data and appear thus to be affected to a minor extent only. Another evidence in this direction is the fact that the additional overexpression of the *pfkA* or/and of the *pflB* gene (but not of the *pgk* gene) in the HC ATPase strain increased the glycolytic flux, as predicted, although this will even further reduce the available proteome pool for other enzymes.

**Figure EV5:**
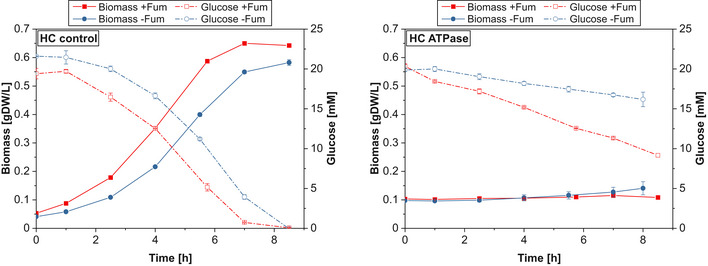
Anaerobic growth of the HC control (left) and HC ATPase (right) strains with (red) and without (blue) fumarate addition The average time courses of biomass and glucose concentrations of *n* = 3 (−Fum) and *n* = 2 (+Fum) biologically independent samples are shown. The error bars represent ± SD. See also Appendix Table S4 for the determined specific rates. Source data are available online for this figure.

To further demonstrate that the lack of ATP and not proteome burden causes the low glycolytic flux in the HC ATPase strain, we cultivated the latter and its control strain anaerobically as before (with glucose as main substrate), but this time with addition of fumarate enabling the strains to gain more ATP (via fumarate respiration). As also suggested by our model, the glycolytic flux in the HC ATPase strain should then increase due to the higher ATP levels fueling the PFK reaction while we do not expect significant changes in the HC control as it is not ATP‐limited. In fact, with addition of fumarate, the glucose uptake rate even decreased slightly in the HC control strain, but increased markedly in the HC ATPase strain by more than 100% almost reaching the level of the control strain (Fig EV5 and Appendix Table S4). The extra amount of glucose was almost completely converted to lactate while the gained ATP was directly consumed by the ATPase as reflected by a high ATPase flux. This result is another strong indicator that it is the low ATP level in the HC ATPase strain rather than ATPase synthesis costs that prevents higher glycolytic fluxes.

The finding that overexpression of the PFK‐ and PFL‐encoding genes may largely increase the glycolytic flux and, especially in the case of PFK, also the growth rate in the HC ATPase strain under anaerobic conditions corroborates, on the one hand, our hypothesis on physiological constraints in this strain but, on the other hand, demonstrates that *E. coli*, as could be expected for the extreme perturbation in the HC ATPase strain, is not for all conditions primed to adjust an optimal expression pattern maximizing its growth rate (cf. Bruggeman *et al*, 2020). Clearly, adaptive laboratory evolution may yield strains that adapt to these physiological changes resulting, for example, in an upregulation of PFK.

Our results are also of high relevance for metabolic engineering strategies that harness the concept of enforced ATP wasting for strain optimization. Several previous works have already demonstrated the potential of increased ATP turnover as a strategy to maximize substrate uptake and product synthesis rates (Chao & Liao, 1994; Koebmann *et al*, 2002; Hädicke *et al*, 2015; Liu *et al*, 2016; Boecker *et al*, 2019, 2021; Zahoor *et al*, 2020). However, our study is the first showing that there is an optimal level of ATPase expression, at which the specific glucose uptake rate and metabolic activity reaches a maximum. To use the full potential of ATP wasting, it will be important to find the precise maximum (which will be specific for the production organism, the respective substrate–product combination, and the chosen conditions) and to properly adjust the optimal level of the ATPase. For each condition tested, the MC ATPase strain showed the highest glucose uptake rate (which are, to the best of our knowledge, for some cultivation conditions, the highest ever reported so far). Our data also reveal that the highest relative increase can be seen for the growth‐arrested cultivations (+1,016% for aerobic and +380% for anaerobic conditions; Fig 3). Moreover, the relative drop in the metabolic activity of the HC ATPase strain is less severe than in the cases with growth indicating a higher robustness against maximal ATPase levels under these conditions. With these findings, we anticipate that the highest potential of enforced ATP wasting lies in the optimization of two‐stage (or even three‐stage (Boecker *et al*, 2021)) processes, in which ATP wasting may greatly boost the activity of the cells in the (growth‐arrested) production phase (Burg *et al*, 2016; Klamt *et al*, 2018).

## Materials and Methods

### Strains and plasmid construction

All strains, plasmids, and primers used in this study are summarized in Appendix Table S1. *E. coli* NEB 5‐alpha competent cells (New England Biolabs, #C2987U) were used for all cloning techniques and plasmid propagation. Standard molecular cloning techniques followed protocols described earlier (Sambrook & Russell, 2001). The ATPase encoding genes *atpAGD* were amplified from plasmid pCP41::*atpAGD* (Koebmann *et al*, 2002) by polymerase chain reaction (PCR) using the Q5 Hot Start High‐Fidelity DNA Polymerase (New England Biolabs, #M0493L) and the primer pair atpAGD_mono_fw/atpAGD_mono_rv as described in (Boecker *et al*, 2019). To construct plasmids pSB58.6 and pSB62.6, *gfpmut3* was cut out from pSB‐T1g and pSB‐T2g (Balzer *et al*, 2013) using restriction enzymes NdeI (New England Biolabs, #R0111S) and BamHI‐HF (New England Biolabs, #R3136S). The *atpAGD* PCR‐amplicon was digested with the same enzymes and ligated into the plasmid backbones of pSB‐T1g and pSB‐T2g using T4 DNA Ligase (New England Biolabs, #M0202S), yielding plasmids pSB58.6 and pSB62.6, respectively. To construct plasmid pSB66.1, the pMB1 replicon was cut out from pSB62.6 using restriction enzymes AscI (New England Biolabs, #R0558S) and SpeI‐HF (New England Biolabs, #R3133S). The p15A replicon was amplified by PCR from plasmid pZA31‐luc (Lutz & Bujard, 1997) using primer pair p15A_SpeI_fw/p15A_AscI_rv. The amplicon was digested with AscI and SpeI‐HF and ligated into the AscI/SpeI‐HF digested plasmid pSB62.6. To construct the control plasmids pSB60.1, pSB64.1, and pSB68.1, *atpAGD* was cut out from pSB58.6, pSB62.6, and pSB66.1 using restriction enzymes NdeI and BamHI‐HF. The 5’‐overhangs were filled‐in using the Klenow Fragment (Thermo Scientific, #EP0054) and the blunt‐ended DNA fragments were self‐ligated. The *E. coli* wild‐type strain MG1655 (Blattner *et al*, 1997) was transformed with the ATPase expression and control plasmids, generating three ATPase expression strains (low, medium, and high) and three control strains (low, medium, and high) with varying plasmid copy numbers.

For additional expression of *pfkA*, *pflB*, and *pgk* together with *atpAGD* in the high copy plasmid pSB62.6, the plasmid was linearized by PCR with the primer pair pSB73.4_Gibson_fw/atpD_rv. The genes encoding *pfkA*, *pflB*, and *pgk* were amplified from the genomic DNA of *E. coli* MG1655 by PCR using the primer pairs pfkA_rbs_fw/pfkA_rbs_rv, pflB_rbs_fw/pflB_rbs_rv, and pgk_rbs_fw/pgk_rbs_fw, respectively. A ribosomal binding site (rbs) from the natural ATPase operon of *E. coli* MG1655 (between *atpD* and *atpC*) was inserted into each forward primer to allow polycistronic expression of *atpAGD* and the respective gene. The linearized plasmid pSB62.6 and the DNA fragments harboring the amplified genes were ligated by Gibson assembly, yielding plasmids pSB84.3, pSB85.3, and pSB88.11 (Appendix Table S1). For co‐expression of *pfkA* and *pflB* together with *atpAGD* in the high copy plasmid, pSB84.3 was linearized by PCR with the primer pair pSB73.4_Gibson_fw/pfkA_rv. *pflB* was amplified from the genomic DNA of *E. coli* MG1655 by PCR using the primer pair pflB_rbs2_fw/pflB_rbs_rv. The same rbs as used above was inserted into the forward primer to allow polycistronic expression of *atpAGD*, *pfkA*, and *pflB* from a single operon. The *pflB*‐harboring DNA fragment and the linearized plasmid pSB84.3 were ligated by Gibson assembly, yielding plasmid pSB86.4 (Appendix Table S1).

### Media and cultivation conditions

All liquid and solid media used for cultivation of the ATPase and control strains contained kanamycin (except for “WT” and “WT + IPTG”) with a final concentration of 50 µg/ml. For growth assays, cells were freshly transformed with the corresponding plasmid and plated on LB_0_ agar plates (10 g/l tryptone, 5 g/l yeast extract, 5 g/l NaCl, 15 g/l agar). A single colony was picked and used to inoculate 5 ml of LB_0_ medium. The medium was incubated at 37°C and 150 rpm for 5 h.

For aerobic cultivation, cells were diluted 1:500 into chemically defined medium (MM: 4 g/l glucose, 34 mM NaH_2_PO_4_, 64 mM K_2_HPO_4_, 20 mM (NH_4_)_2_SO_4_, 1 μM Fe(SO_4_)_4_, 300 μM MgSO_4_, 1 μM ZnCl_2_, 10 μM CaCl_2_, adapted from (Tanaka *et al*, 1967)), containing 0.01 mM of IPTG (except for “WT”) and cultivated at 37°C and 250 rpm overnight. The cells were centrifuged at 5,000 *g*, washed, and used to inoculate 25 ml of fresh MM (containing 0.01 mM IPTG, except for “WT”) to an optical density at 420 nm (OD_420_) of 0.2 (0.4 for the HC ATPase strain). The cells were cultivated in 250‐ml shake flasks with three baffles at 37°C and 250 rpm.

For anaerobic cultivation, cells from the LB_0_‐culture were diluted 1:100 into MM (containing 0.01 mM IPTG, except for “WT”) and cultivated at 37°C without shaking overnight. The cells were centrifuged at 5,000 *g*, washed, and used to inoculate fresh MM (containing 0.01 mM IPTG, except for “WT”) to an OD_420_ of 0.2 (0.4 for the HC ATPase and *pfkA*, *pflB*, or *pgk* co‐expressing strains). The medium was filled into 5‐ml screw‐cap glass vials (completely filled to the top), and the vials were incubated at 37°C without shaking. For every time point, new vials were opened to guarantee anaerobic conditions.

For cultivation of growth‐arrested cells, the same procedures for aerobic and anaerobic cultivation were followed as described above, but MM without added (NH_4_)_2_SO_4_ and an initial OD_420_ of 2.0 were used for cultivation.

For anaerobic cultivations in medium containing additionally fumarate, the cells were cultivated as described above, but MM supplemented with 20 mM of fumarate was used for the overnight and main cultures.

Cell growth was monitored measuring the OD_420_ and using a factor of 0.22 to convert one OD_420_ unit to gram dry weight per liter (gDW/l). All cultivations were performed in biological triplicates, if not stated otherwise.

### Analytical methods

Extracellular glucose, ethanol, acetate, formate, succinate, lactate, pyruvate, and fumarate in the medium were quantified as described earlier (Boecker *et al*, 2019). Orotate was quantified by the same method but was not secreted in significant amounts by the strains.

For quantification of intracellular metabolites (except pyruvate), cells (~0.5 mg of biomass, from mid‐exponential growth phase, growth conditions as described above) were applied to filter disks (Merck Millipore, #HVLP02500) under constant nitrogen flow to keep anaerobic conditions. The medium was removed by suction filtration, and the filter disks were immediately transferred to 1 ml of a −20°C cold acetonitrile/methanol/water (40:40:20) quenching solution. After incubation at −20°C for at least 30 min, the samples were shaken vigorously, and 500 μl of the mixture was centrifuged at 17,000 *g* and −9°C for 15 min. Next, 400 μl of the supernatant was kept at −80°C until metabolite quantification. Extracts were mixed with a ^13^C‐labeled internal standard in a 1:1 ratio and analyzed by liquid chromatography‐tandem mass spectrometry, which was performed as previously described (Guder *et al*, 2017) using an Agilent 6495 triple quadrupole mass spectrometer (Agilent Technologies). The ratio of ^12^C and ^13^C peak heights was used to quantify metabolites. ^12^C/^13^C ratios were normalized to OD at the time point of sampling. Absolute ATP, ADP, and AMP concentrations were determined with the ^13^C internal standard and authentic standards (Guder *et al*, 2017). A specific cell volume of 2 µl/mg was used to calculate the cell volume. The intracellular adenosine energy charge was calculated with the formula ([ATP] + 0.5[ADP])/([ATP] + [ADP] + [AMP]). For quantification of intracellular pyruvate, cell extracts were prepared as described above, but ~1 mg of biomass and 2 ml of the quenching solution were used. 1.7 ml of the extract were centrifuged at 17,000 *g* and −9°C for 15 min and 1.5 ml of the supernatant transferred to a new test tube. The solvents were evaporated in a speed‐vac and the residues dissolved in 55 µl of H_2_O. Absolute pyruvate concentrations were determined using the pyruvic acid assay kit (Megazyme, #K‐PYRUV) and normalized to OD at the time point of sampling.

For proteomics analysis, cells were cultivated as described above. ~1 × 10^9^ cells were harvested by centrifugation (3 min, 4°C, 17,000 *g*), the supernatant discarded and the cells resuspended in 2 ml of ice‐cold PBS buffer. The cells were centrifuged again (3 min, 4°C, 17,000 *g*) and the PBS‐washing step was repeated twice. Cell pellets were stored at −80°C until further analysis.

The pelletized cells were lysed in 400 µl of 2% sodium lauroyl sarcosinate (SLS) in 100 mM ammonium bicarbonate by heat (20 min, 90°C) and sonication. After 10 min of centrifugation at 17,000 *g*, the protein concentration in the supernatant was determined with a bicinchoninic acid (BCA)‐based protein assay kit (Thermo Fisher, #23252). 7.5 µl of 0.2 M tris(carboxylethyl)phosphine in 100 mM ammonium carbonate were added to 300 µl of the supernatant. The solution was incubated for 15 min at 90°C. 7.5 µl of 74 mg/ml iodoacetamide were added to the cooled‐off samples and incubated for 30 min at 25°C under shaking of 500 rpm. Using 2% SLS in 100 mM ammonium bicarbonate, 200 µl aliquots of the samples were prepared containing a total protein mass of 50 µg. 600 µl of 100 mM ammonium bicarbonate and 8.5 µl of 0.1 µg/µl porcine trypsin were added to the samples for incubation overnight at 30°C.

5% trifluoroacetic acid was added to the samples to a final concentration of 1.5%. After incubation for 10 min at room temperature, the samples were centrifuged at 17,000 *g* for 10 min at 4°C. The supernatant was used for solid phase extraction of the peptides using C18‐columns (Macherey‐Nagel).

Peptides were analyzed using a Q‐Exactive Plus mass spectrometer connected to an Ultimate 3000 RSLC nano and a nanospray flex ion source (Thermo Scientific). The analytical setting was reported in detail previously (Donati *et al*, 2021). In short, peptide separation was performed on a reverse‐phase HPLC column (75 μm × 42 cm) packed in‐house with C18 resin (2.4 μm, Dr. Maisch GmbH, Germany). The following separating gradient was used: 96% solvent A (0.15% formic acid) and 4% solvent B (99.85% acetonitrile, 0.15% formic acid) to 30% solvent B over 60 min at a flow rate of 300 nl/min.

The data acquisition mode was set with the following parameters: 1 MS scan at a resolution of 70,000 with 50 ms max. ion injection fill time, MS/MS at 17,500 scans of the 10 most intense ions with 50 ms max. fill time. Label‐free quantification (LFQ) of the data was performed using Progenesis QIP (Waters) and MASCOT (v2.5, Matrix Science) for spectrum/peptide identification. Progenesis outputs were further processed with SafeQuant (Glatter *et al*, 2012; Ahrné *et al*, 2016).

### Calculation of growth rate, specific exchange rates, and yields

For experiments with growth, the growth rate (*µ*) for the exponential phase was determined by plotting the natural logarithm of the biomass concentrations of each sampled time point (within the exponential growth period) against the cultivation time. The slope of the linear regression equals *µ*.

Specific uptake and excretion rates for the exponential phase in growth‐coupled experiments were determined with the formula:

$$r_{M}=\mu \left(c_{\text{M,e}}‐c_{\text{M,s}}\right)/\left(c_{\text{X,e}}‐c_{\text{X,s}}\right)\;\left[\text{mmol/gDW/h}\right]$$

where *µ* is the growth rate, *c*_M,e_ and *c*_M,s_ represent the end and start concentrations of the respective metabolite M (mmol/l glucose, ethanol, acetate, formate, lactate, succinate, pyruvate, or fumarate), and *c*_X,e_ and *c*_X,s_ represent the end and start concentrations of the biomass (gDW/l). In experiments with growth arrest, where the biomass concentration remained nearly constant, the specific rates are calculated as:

$$r_{M}=\left(c_{\text{M,e}}‐c_{\text{M,s}}\right)/X_{\text{Av}}/\Delta t\;\left[\text{mmol/gDW/h}\right]$$

where *X*_Av_ is the average biomass concentration (gDW/l), and Δ*t* = *t*_e_ − *t*_s_ the length of the time period (difference of end and start time). This procedure was used for each of the three replicates from which then the mean and the standard deviation was calculated for each rate.

Metabolite yields were determined by plotting Δ*c*_M_ (mmol/l) against Δ*c*_Glc_ (mmol/l) for every sampled time point of the exponential growth period (for growth‐coupled cultivation) or of the indicated time period (of growth‐arrested cultivation). The slope of the linear regression equals the yield of the respective metabolite. Biomass yields were determined by plotting Δ*c*_X_ (gDW/l) against Δ*c*_Glc_ (g/l) for every sampled time point of the exponential growth period (for growth‐coupled cultivation). The slope of the linear regression equals the biomass yield.

### Statistical analysis

Unless stated otherwise, *P*‐values for comparisons between different strains were calculated using an unpaired two‐sample *t*‐test with the software OriginPro (version 2020b, OriginLab Corporation). Statistical details of the individual experiments can be found in the captions of the respective tables and figures.

### Metabolic flux analysis to determine ATP turnover rates through the ATPase

Using a stoichiometric model of the central metabolism of *E. coli* (83 reactions and 54 internal metabolites; adapted from (Hädicke & Klamt, 2017)) and the MATLAB (MathWorks, version R2020b) toolbox *CellNetAnalyzer* (Klamt *et al*, 2007; von Kamp *et al*, 2017), metabolic flux analysis based on the experimentally determined exchange rates was performed to estimate the ATPase flux in the different strains. In stoichiometric models, unspecific ATP consumption (which includes the non‐growth‐associated maintenance (NGAM) demand of ATP) is usually represented by an “ATPM” pseudo reaction hydrolyzing ATP. In the ATPase strains, the estimated flux through this reaction in the stoichiometric model contains both the NGAM demand as well as the actual ATPase flux and the latter can thus be calculated as the difference of the calculated ATPM flux in the ATPase strains and the calculated ATPM flux in the corresponding control strains. Herein, it was assumed that, after consideration of the measured growth rate and exchange fluxes, the remaining degrees in the network were used by the cell to produce a maximum amount of ATP (which is accounted for by maximizing the ATPM flux under the given constraints). When performing these calculations, it happens (especially for anaerobic conditions) that the experimentally determined rates contradict each other (e.g., due to linear dependencies). In those cases, *CellNetAnalyzer* can be used to minimally adjust the measured rates to obtain a consistent scenario (“Check feasibility” function) before the ATPM flux is maximized. The stoichiometric model together with a detailed description of the calculations is provided on GitHub (see Data availability).

### Kinetic model

The two versions for the kinetic model were implemented and simulated with COPASI (Hoops *et al*, 2006) and are described in detail in the Appendix Supplementary Text. The model files are also provided on GitHub (see Data availability).

## Author contributions

SK conceived and supervised the study. SB performed plasmid and strain construction, cultivations, external metabolite quantification, sampling for proteomics and metabolomics analysis, and determination of metabolites fluxes. GS constructed the kinetic model and carried out the simulations. RS helped in model construction and the Monte Carlo‐based sampling of kinetic parameters. TS and HL generated the metabolomics data. TS and WS generated the proteomic data. SB, GS, and SK analyzed the data and wrote the manuscript. SB and GS generated the figures. All authors discussed and approved the content of the manuscript.

## Supporting information



Appendix

Expanded View Figures PDF

Dataset EV1

Source Data for Expanded View and Appendix

Source Data for Figure 1

Source Data for Figure 2

Source Data for Figure 7

## Data Availability

The kinetic models (provided in COPASI and SBML format) as well as the stoichiometric model used for calculating the ATPase fluxes (provided as *CellNetAnalyzer* project and as SBML file) are available under the following GitHub repository: https://github.com/klamt‐lab/Models_E.coli_High_ATP_Demand Metabolomics MS data: Edmond Repository [Dyld9hM3KIMXqRg2] https://edmond.mpdl.mpg.de/imeji/collection/Dyld9hM3KIMXqRg2 Proteomics MS data: MassIVE Repository MSV000088475 https://massive.ucsd.edu/ProteoSAFe/dataset.jsp?accession=MSV000088475
