# Supplementary material for: Deciphering the physiological response of Escherichia coli under high ATP demand
Source: Mol Syst Biol. 2021 Dec 20;17(12):e10504. doi: 10.15252/msb.202110504 (PMC8686765; doi:10.15252/msb.202110504)
Supplement: Supplementary file 1 — Appendix [file MSB-17-e10504-s001.pdf]

Appendix

Deciphering the Physiological Response of *Escherichia coli*
Under High ATP Demand

Simon Boecker, Giulia Slaviero, Thorben Schramm,
Witold Szymanski, Ralf Steuer, Hannes Link, Steffen Klamt*

*) correspondence: klamt@mpi-magdeburg.mpg.de

This PDF file includes:

- Supplementary Text (Detailed documentation of kinetic model)
- Appendix Tables S1 to S4
- References

Supplementary Text

This Supplementary Text describes the two versions of the kinetic model of the central metabolism of *E. coli* under anaerobic conditions as used in the main text. We first describe version 1 before summarizing the changes introduced in version 2 and showing the results of the metabolic control analysis obtained with version 2.

1. Kinetic model version 1

1.1 Overview

The kinetic model describes the core carbon and energy metabolism of *Escherichia coli* during the exponential growth phase under anaerobic conditions. The model contains 33 metabolites and 28 reactions and consists of the two compartments cytoplasm and external environment (Figure S1). The following major pathways are included: glucose phosphotransferase system (PTS), glycolysis, two anaplerotic reactions (PEP carboxylase and PEP carboxykinase), left and right branch of the tricarboxylic acid (TCA) cycle active under anaerobic conditions, the main fermentative pathways (leading to the fermentation products lactate, ethanol, acetate, formate and succinate), transport reactions between cytoplasm and external environment, a biomass synthesis reaction, a NGAM reaction that accounts for non-growth associated maintenance ATP demands, and finally an ATPase reaction that hydrolyses ATP into ADP and whose activity depends on the level of the overexpression of the ATPase genes. Not included were gluconeogenic reactions, the Entner-Doudoroff (ED) pathway, the glyoxylate shunt (GS) and oxidative phosphorylation as they are not relevant for anaerobic growth on glucose. Furthermore, we did not include the pentose phosphate pathway (PPP), as it has, compared to glycolysis, only relatively low flux under anaerobic conditions. However, we explicitly account for the requirement of precursors (e.g. erythrose-4-phoshphate) from the PPP by adding a stoichiometrically equivalent efflux of other metabolites contained in the model (see description of the growth rate in section 1.3). Furthermore, since the PPP was not included, the reduction equivalents NADH and NADPH were lumped in one pool (referred to as NADH), which ensures that the model accounts for overall redox balance. To reduce the size of the model, some reaction steps were partially lumped as described below.

The two models used herein were implemented and simulated with Copasi (Hoops *et al*, 2006), version 4.28, and the two model versions are available as *.cps* (Copasi) files as well as in SBML format at
https://github.com/klamt-lab/Models_E.coli_High_ATP_Demand/tree/main/Kinetic%20Model.

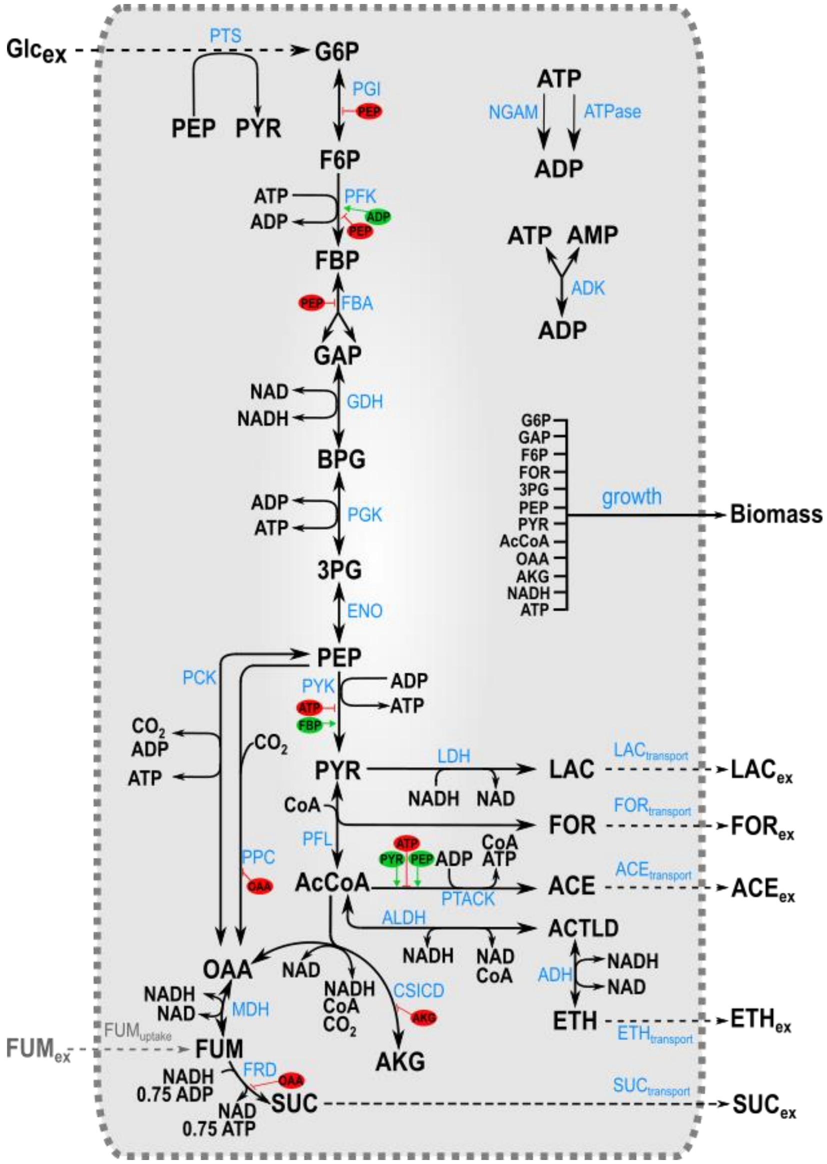

Figure S1: Schematic of the central anaerobic carbon and energy metabolism of *E. coli* implemented in the model. Metabolites are shown in black; reactions are reported in blue and activators and inhibitors are shown in green and red, respectively. Abbreviations of metabolites and reaction names (as also used throughout this document): Glc_{ex}: external glucose (substrate); G6P: D-glucose-6-phosphate; F6P: D-fructose-6-phosphate; FBP: fructose-1,6-bisphosphate; GAP: D-glyceraldehyde-3-phosphate; BPG: 1,3-bisphospho-D-glycerate; 3PG: 3-phosphoglycerate; PEP: phosphoenolpyruvate; PYR: pyruvate; AcCoA: acetyl coenzyme A; CoA: coenzyme A; AKG: α-ketoglutarate; OAA: oxaloacetate; FUM: fumarate; SUC: succinate (intracellular); FOR: formate (intracellular); LAC: lactate (intracellular); ACE: acetate (intracellular); ACTLD: acetaldehyde; ETH: ethanol (intracellular); FOR_{ex}: formate (extracellular); ETH_{ex}: ethanol (extracellular); ACE_{ex}: acetate (extracellular); LAC_{ex}: lactate (extracellular); SUC_{ex}: succinate (extracellular); FUM_{ex}: fumarate (extracellular): this metabolite was only included when simulating co-utilization of glucose and fumarate uptake by the HC ATPase strain (see main manuscript); ATP: adenosine triphosphate; ADP: adenosine diphosphate; AMP: adenosine monophosphate; NAD: lumped pool of oxidized nicotinamide adenine dinucleotide (NAD) and oxidized nicotinamide adenine dinucleotide phosphate (NADP); NADH: lumped pool of reduced nicotinamide adenine dinucleotide (NADH) and reduced nicotinamide adenine dinucleotide phosphate (NADPH); CO_2: carbon dioxide (fixed concentration). PTS: phoshotransferase system; PGI: glucose-6-phosphate isomerase; PFK: phosphofructokinase; FBA: fructose-bisphosphate aldolase (the associated reaction was lumped with the reaction of the triose-phosphate isomerase (TPI) thus yielding two molecules of GAP); GHD: glyceralde-hyde-3-phosphate dehydrogenase; PGK: phosphoglycerate kinase; ENO: enolase (the reaction of this

enzyme was lumped with the reaction of the phosphoglycerate mutase); PYK: pyruvate kinase; PFL: pyruvate formate lyase (also known as formate acteyltransferase); LDH: lactate dehydrogenase; PTACK: lumped reaction of acetate kinase and phosphate acetyltransferase; ALDH: acetaldehyde-CoA dehydrogenase; ADH: alcohol dehydrogenase; PCK: phosphoenolpyruvate carboxykinase; PPC: phosphoenolpyruvate carboxylase; CSICD: lumped reaction of citrate synthase, aconitate hydratase A, aconitate hydratase B and isocitrate dehydrogenase; MDH: lumped reaction of malate dehydrogenase and fumarase; FRD: lumped reaction of fumarate reductase and of other reactions involved in fumarate reduction; $SUC_{transport}$: succinate excretion; $LAC_{transport}$: lactate excretion; $FOR_{transport}$: formate excretion; $ACE_{transport}$: acetate excretion; $ETH_{transport}$: ethanol excretion; growth: biomass synthesis reaction (its rate will be indicated with μ); NGAM: consumption of ATP for non-growth associated maintenance; ATPase: reaction that hydrolyses ATP to ADP catalyzed by the ATPase (the latter being expressed in varying levels in the different strains); ADK: adenylate kinase. Orthophosphate, water, and protons are not shown since these compounds are not explicitly considered in the model. FUM_{uptake}: fumarate uptake (this reaction was only included when simulating co-utilization of glucose and fumarate uptake by the HC ATPase strain (see main manuscript)).

1.2 Mass balances and differential equations

The unit for concentrations of internal metabolites is [µmol/gDW] (DW: dry weight) whereas [g/L] is used for external metabolites (substrates and products). Units of measured internal metabolite concentrations and of some known parameters (from databases, literature etc.) given in molar concentrations were converted to [µmol/gDW] by considering a cytosolic density of 564 $\frac{g_{DW}}{L}$ as reported in (Chassagnole *et al*, 2002). Internal metabolic fluxes are given in [µmol/gDW/s] and converted to volumetric fluxes [g/L/s] for exchange reactions of external metabolites by using the molecular weight ([g/mol]) of the respective compound.
The differential equations given below (and the reactions shown in Figure S1) do not account for phosphate, water and protons since these compounds are not explicitly considered in the model.

$$\frac{d[\text{Biomass}]}{dt} = [\text{Biomass}] \cdot \mu$$
$$\frac{d[\text{Glc}_\text{ex}]}{dt} = -v_{PTS} \cdot \frac{[\text{Biomass}] \cdot MW_{Glucose}}{10^6}$$
$$\frac{d[\text{FOR}_\text{ex}]}{dt} = v_{FOR,transport} \cdot \frac{[\text{Biomass}] \cdot MW_{FOR}}{10^6}$$
$$\frac{d[\text{ACE}_\text{ex}]}{dt} = v_{ACE,transport} \cdot \frac{[\text{Biomass}] \cdot MW_{ACE}}{10^6}$$
$$\frac{d[\text{ETH}_\text{ex}]}{dt} = v_{ETH,transport} \cdot \frac{[\text{Biomass}] \cdot MW_{ETH}}{10^6}$$
$$\frac{d[\text{LAC}_\text{ex}]}{dt} = v_{LAC,transport} \cdot \frac{[\text{Biomass}] \cdot MW_{LAC}}{10^6}$$
$$\frac{d[\text{SUC}_\text{ex}]}{dt} = v_{SUC,transport} \cdot \frac{[\text{Biomass}] \cdot MW_{SUC}}{10^6}$$
$$\frac{d[\text{FOR}]}{dt} = v_{PFL} - v_{FOR,transport} - 107.59 \cdot \mu - \mu \cdot [\text{FOR}]$$
$$\frac{d[\text{ACE}]}{dt} = v_{PTACK} + 581.01 \cdot \mu - v_{ACE,transport} - \mu \cdot [\text{ACE}]$$
$$\frac{d[\text{ETH}]}{dt} = v_{ADH} - v_{ETH,transport} - \mu \cdot [\text{ETH}]$$
$$\frac{d[\text{LAC}]}{dt} = v_{LDH} - v_{LAC,transport} - \mu \cdot [\text{LAC}]$$
$$\frac{d[\text{SUC}]}{dt} = v_{FRD} + 1040.71 \cdot \mu - v_{SUC,transport} - \mu \cdot [\text{SUC}]$$
$$\frac{d[\text{G6P}]}{dt} = v_{PTS} - v_{PGI} - 600.28 \cdot \mu - \mu \cdot [\text{G6P}]$$
$$\frac{d[\text{F6P}]}{dt} = v_{PGI} - v_{PFK} - 466.42 \cdot \mu - \mu \cdot [\text{F6P}]$$
$$\frac{d[\text{FBP}]}{dt} = v_{PFK} - v_{FBA} - \mu \cdot [\text{FBP}]$$
$$\frac{d[\text{GAP}]}{dt} = 2 \cdot v_{FBA} - v_{GDH} - 459.27 \cdot \mu - \mu \cdot [\text{GAP}]$$
$$\frac{d[\text{BPG}]}{dt} = v_{GDH} - v_{PGK} - \mu \cdot [\text{BPG}]$$
$$\frac{d[\text{3PG}]}{dt} = v_{PGK} - v_{ENO} - 1717.48 \cdot \mu - \mu \cdot [\text{3PG}]$$

$$\frac{d[\mathrm{PEP}]}{dt} = v_{ENO} + v_{PCK} - v_{PYK} - v_{PPC} - v_{PTS} - 810.19 \cdot \mu - \mu \cdot [\mathrm{PEP}]$$
$$\frac{d[\mathrm{PYR}]}{dt} = v_{PTS} + v_{PYK} - v_{PFL} - v_{LDH} - 2784.13 \cdot \mu - \mu \cdot [\mathrm{PYR}]$$
$$\frac{d[\mathrm{AcCoA}]}{dt} = v_{PFL} - v_{PTACK} - v_{ALDH} - v_{CSICD} - 3856.57 \cdot \mu - \mu \cdot [\mathrm{AcCoA}]$$
$$\frac{d[\mathrm{ACTLD}]}{dt} = v_{ALDH} - v_{ADH} - \mu \cdot [\mathrm{ACTLD}]$$
$$\frac{d[\mathrm{OAA}]}{dt} = v_{PPC} - v_{MDH} - v_{CSICD} - v_{PCK} - 2924.97 \cdot \mu - \mu \cdot [\mathrm{OAA}]$$
$$\frac{d[\mathrm{AKG}]}{dt} = v_{CSICD} - 1075.32 \cdot \mu - \mu \cdot [\mathrm{AKG}]$$
$$\frac{d[\mathrm{FUM}]}{dt} = v_{MDH} - v_{FRD} - \mu \cdot [\mathrm{FUM}]$$
$$\frac{d[\mathrm{ATP}]}{dt} = v_{PTACK} + 0.75 \cdot v_{FRD} + v_{PGK} + v_{PYK} - v_{PFK} - v_{PCK} - v_{NGAM} - v_{ATPase} - v_{ADK} - 53107.60 \cdot \mu - \mu \cdot [\mathrm{ATP}]$$
$$\frac{d[\mathrm{ADP}]}{dt} = v_{PFK} + v_{PCK} + v_{NGAM} + v_{ATPase} + 2 \cdot v_{ADK} - v_{PTACK} - 0.75 \cdot v_{FRD} - v_{PGK} - v_{PYK} - 49739.50 \cdot \mu - \mu \cdot [\mathrm{ADP}]$$
$$\frac{d[\mathrm{NADH}]}{dt} = v_{GDH} + v_{CSICD} - v_{ADH} - v_{ALDH} - v_{LDH} - v_{FRD} - v_{MDH} - 14889.30 \cdot \mu - \mu \cdot [\mathrm{NADH}]$$

The following equations show the conservation rules of the conserved moieties contained in the model. These algebraic equations were used to balance the species [AMP], [NAD], and [CoA].

$[\mathrm{ATP}] + [\mathrm{ADP}] + [\mathrm{AMP}]$	= 2.70 µmol/gDW	(Measured in this study)
$[\mathrm{NAD}] + [\mathrm{NADH}]$	= 2.78 µmol/gDW	(Chassagnole *et al*, 2002)
$[\mathrm{AcCoA}] + [\mathrm{CoA}]$	= 3.49 µmol/gDW	(Park *et al*, 2016)

1.3 Reactions and rate laws

PTS reaction

$$\mathrm{Glc_{ex}} + \mathrm{PEP} \leftrightarrow \mathrm{PYR} + \mathrm{G6P}$$

The glucose phosphotransferase system (PTS) is the main glucose uptake mechanism in *E. coli*. The overall reaction is modeled using Michaelis-Menten kinetics. Inhibition terms for G6P reflect reduced uptake when and G6P accumulates.

$$v_{PTS} = v_{max,PTS} \cdot \frac{\frac{[\mathrm{PEP}]}{k_{PTS,PEP}} \cdot \frac{[\mathrm{Glc_{ex}}]}{k_{PTS,Glc}} \cdot \left(1 - \frac{\frac{[\mathrm{G6P}]\,[\mathrm{PYR}]}{[\mathrm{PEP}]\,[\mathrm{Glcex}]}}{k_{PTS,eq}}\right)}{1 + \frac{[\mathrm{PEP}]}{k_{PTS,PEP}} + \frac{[\mathrm{Glc_{ex}}]}{k_{PTS,Glc}} + \frac{[\mathrm{PYR}]}{k_{PTS,PYR}} + \left(\frac{[\mathrm{G6P}]}{k_{PTS,G6P}}\right)^{n_{PTS,G6P}}}$$

**** Here and in the following ** means that the parameter has been estimated in this study by fitting the parameters to the experimental data (see also section 3).**

Parameter	Value	Unit	Reference
$v_{max,PTS}$	13.53	[µmol/gDW/s]	**
$k_{PTS,eq}$	12	[-]	(Rohwer *et al*, 2000)
$k_{PTS,PEP}$	0.531	[µmol/gDW]	(Rohwer *et al*, 2000)
$k_{PTS,Glc}$	0.0345	[µmol/gDW]	(Rohwer *et al*, 2000)
$k_{PTS,G6P}$	1.0625	[µmol/gDW]	(Bettenbrock *et al*, 2006)
$k_{PTS,PYR}$	3.54	[µmol/gDW]	(Rohwer *et al*, 2000)
$n_{PTS,G6P}$	2.21	[-]	**

PGI reaction

$$\mathrm{G6P} \leftrightarrow \mathrm{F6P}$$

The isomerization of G6P into F6P is described using Michaelis-Menten kinetics as in (Peskov *et al*, 2012). As observed in (Ogawa *et al*, 2007), PEP acts as inhibitor for the reaction.

$$v_{PGI} = v_{max,PGI} \cdot \frac{\frac{[\mathrm{G6P}]}{k_{PGI,G6P}} \cdot \left(1 - \frac{\frac{[\mathrm{F6P}]}{[\mathrm{G6P}]}}{k_{PGI,EQ}}\right)}{1 + \frac{[\mathrm{G6P}]}{k_{PGI,G6P}} + \frac{[\mathrm{F6P}]}{k_{PGI,F6P}} + \frac{[\mathrm{PEP}]}{k_{PGI,PEP}}}$$

Parameter	Value	Unit	Reference
$v_{max,PGI}$	18.71	[µmol/gDW/s]	**
$k_{PGI,EQ}$	0.3	[-]	(Noor *et al*, 2013)
$k_{PGI,G6P}$	0.31	[µmol/gDW]	(Ogawa *et al*, 2007)
$k_{PGI,F6P}$	0.293	[µmol/gDW]	(Ishii *et al*, 2007)
$k_{PGI,PEP}$	0.46	[µmol/gDW]	(Ogawa *et al*, 2007)

PFK reaction

$$\mathrm{F6P} + \mathrm{ATP} \leftrightarrow \mathrm{FBP} + \mathrm{ADP}$$

The PFK reaction catalyzes the phosphorylation of F6P and is a key enzyme for the glycolysis pathway and regulated in different ways. As reported in (Peskov *et al*, 2008),

- a complex of ATP with magnesium ions ($ATPMg^{2+}$) acts as real substrate for PFK;
- the reaction rate depends sigmoidal on the F6P concentration;
- PFK is allosterically activated by ADP and inhibited by PEP.

To keep track of all these effects, a modular convenience kinetics is used (Liebermeister *et al*, 2010). For simplicity, ATP and ADP are directly used to avoid the introduction of the magnesium complexes.

$$v_{PFK} = v_{max,PFK} \cdot \left(\frac{[\mathrm{ADP}]}{k_{PFK,a,ADP} + [\mathrm{ADP}]}\right) \cdot \left(\frac{k_{PFK,i,PEP}}{k_{PFK,i,PEP} + [\mathrm{PEP}]}\right)^{n_{PFK,i,PEP}} \cdot \left(\frac{\frac{[\mathrm{ATP}]}{k_{PFK,ATP}} \cdot \left(\frac{[\mathrm{F6P}]}{k_{PFK,F6P}}\right)^{n_{PFK,F6P}} \cdot \left(1 - \frac{\frac{[\mathrm{ADP}]\,[\mathrm{FBP}]}{[\mathrm{ATP}]\,[\mathrm{F6P}]^{n_{PFK,F6P}}}}{k_{PFK,EQ}}\right)}{\left(1 + \frac{[\mathrm{ATP}]}{k_{PFK,ATP}}\right) \cdot \left(1 + \frac{[\mathrm{F6P}]}{k_{PFK,F6P}}\right)^{n_{PFK,F6P}} + \left(1 + \frac{[\mathrm{FBP}]}{k_{PFK,FBP}}\right) \cdot \left(1 + \frac{[\mathrm{ADP}]}{k_{PFK,ADP}}\right) - 1}\right)$$

Parameter	Value	Unit	Reference
$v_{max,PFK}$	17.74	[µmol/gDW/s]	**
$k_{PFK,EQ}$	1300	[-]	(Noor *et al*, 2013)
$k_{PFK,ATP}$	1.137	[µmol/gDW]	**
$k_{PFK,F6P}$	0.0531	[µmol/gDW]	(Berger & Evans, 1991)
$k_{PFK,FBP}$	5	[µmol/gDW]	**
$k_{PFK,ADP}$	4.28	[µmol/gDW]	**

$k_{PFK,a,ADP}$	0.2886	[µmol/gDW]	**
$k_{PFK,i,PEP}$	3.45	[µmol/gDW]	(Ogawa *et al*, 2007)
$n_{PFK,i,PEP}$	2.61	[-]	**
$n_{PFK,F6P}$	1.9	[-]	(Ishii *et al*, 2007)

Note that this (isolated) PFK kinetics was also used to generate Figure 4 in the main document.

FBA reaction

$$\mathrm{FBP} \leftrightarrow 2\ \mathrm{GAP}$$

This reaction lumps together the FBA and TPI (triose-phosphate isomerase) reactions and is described using a direct binding convenience rate law (Liebermeister *et al*, 2010). According to (Ogawa *et al*, 2007), an inhibition term for PEP was introduced.

$$v_{FBA} = \frac{v_{max,FBA,F} \cdot \frac{[\mathrm{FBP}]}{k_{FBA,FBP}} \cdot \left(1 - \frac{\frac{[\mathrm{GAP}]^2}{[\mathrm{FBP}]}}{k_{FBA,EQ}}\right)}{1 + \frac{[\mathrm{FBP}]}{k_{FBA,FBP}} + \left(\frac{[\mathrm{GAP}]}{k_{FBA,GAP}}\right)^2 + \left(\frac{[\mathrm{PEP}]}{k_{FBA,PEP}}\right)^{n_{FBA,PEP}}}$$

Parameter	Value	Unit	Reference
$v_{max,FBA,F}$	19	[µmol/gDW/s]	**
$k_{FBA,FBP}$	40	[µmol/gDW]	**
$k_{FBA,GAP}$	16.6	[µmol/gDW]	(Kotte *et al*, 2010)
$k_{FBA,PEP}$	0.81	[µmol/gDW]	**
$n_{FBA,PEP}$	3	[-]	**
$k_{FBA,EQ}$	0.177	[µmol/gDW]	(Babul *et al*, 1993)

GDH reaction

$$\mathrm{GAP} + \mathrm{NAD} \leftrightarrow \mathrm{BPG} + \mathrm{NADH}$$

This reaction is described using convenience kinetics.

$$v_{GDH} = v_{max,GDH} \cdot \frac{\frac{[\mathrm{GAP}]}{k_{GDH,GAP}} \cdot \frac{[\mathrm{NAD}]}{k_{GDH,NAD}} \cdot \left(1 - \frac{\frac{[\mathrm{BPG}]\ [\mathrm{NADH}]}{[\mathrm{GAP}][\mathrm{NAD}]}}{k_{GDH,EQ}}\right)}{\left(1 + \frac{[\mathrm{NAD}]}{k_{GDH,NAD}} + \frac{[\mathrm{NADH}]}{k_{GDH,NADH}}\right) \cdot \left(1 + \frac{[\mathrm{GAP}]}{k_{GDH,GAP}} + \frac{[\mathrm{BPG}]}{k_{GDH,BPG}}\right)}$$

Parameter	Value	Unit	Reference
$v_{max,GDH,F}$	20.86	[µmol/gDW/s]	**
$k_{GDH,EQ}$	1679	[-]	(Noor *et al*, 2013)
$k_{GDH,GAP}$	0.0983	[µmol/gDW]	**
$k_{GDH,NAD}$	0.0194	[µmol/gDW]	(Millard *et al*, 2017; Wright *et al*, 1995)
$k_{GDH,BPG}$	0.354	[µmol/gDW]	(Lambeir *et al*, 1991)
$k_{GDH,NADH}$	6.549	[µmol/gDW]	(Millard *et al*, 2017)

PGK reaction

$$\mathrm{BPG} + \mathrm{ADP} \leftrightarrow \mathrm{3PG} + \mathrm{ATP}$$

This reaction is modeled using a Random Bi-Bi kinetics as used in (Peskov *et al*, 2012).

$$v_{PGK} = v_{max,PGK}\ \frac{\frac{[\mathrm{BPG}]\cdot[\mathrm{ADP}]}{k_{PGK,BPG}\cdot k_{PGK,ADP}}\cdot\left(1-\frac{\frac{[\mathrm{ATP}]\cdot[\mathrm{3PG}]}{[\mathrm{BPG}]\cdot[\mathrm{ADP}]}}{k_{PGK,EQ}}\right)}{D_{PGK}}$$

$$D_{PGK} = 1 + \frac{[\mathrm{ADP}]}{k_{PGK,ADP}} + \frac{[\mathrm{BPG}]}{k_{PGK,BPG}} + \frac{[\mathrm{BPG}]\cdot[\mathrm{ADP}]}{k_{PGK,BPG}\cdot k_{PGK,ADP}} + \frac{[\mathrm{3PG}]}{k_{PGK,3PG}} + \frac{[\mathrm{ATP}]}{k_{PGK,ATP}} + \frac{[\mathrm{3PG}]\cdot[\mathrm{ATP}]}{k_{PGK,ATP}\cdot k_{PGK,3PG}}$$

Parameter	Value	Unit	Reference
$v_{max,PGK}$	28.9	[µmol/gDW/s]	**
$k_{PGK,EQ}$	2700	[-]	(Noor *et al*, 2013)
$k_{PGK,BPG}$	0.3186	[µmol/gDW]	(Kuntz & Krietsch, 1982)
$k_{PGK,ADP}$	0.0122	[µmol/gDW]	**
$k_{PGK,3PG}$	2.265	[µmol/gDW]	(Kuntz & Krietsch, 1982)
$k_{PGK,ATP}$	2.1996	[µmol/gDW]	**

ENO reaction

$$\mathrm{3PG} \leftrightarrow \mathrm{PEP}$$

This reaction lumps together the reactions of 2,3-bisphosphoglycerate phosphoglycerate mutase and of enolase and its kinetics is described using reversible Michaelis-Menten kinetics.

$$v_{ENO} = v_{max,ENO}\cdot\frac{\frac{[\mathrm{3PG}]}{k_{ENO,3PG}}\cdot\left(1-\frac{\frac{[\mathrm{PEP}]}{[\mathrm{3PG}]}}{k_{ENO,EQ}}\right)}{1+\frac{[\mathrm{3PG}]}{k_{ENO,3PG}}+\frac{[\mathrm{PEP}]}{k_{ENO,PEP}}}$$

Parameter	Value	Unit	Reference
$v_{max,ENO}$	32.12	[µmol/gDW/s]	**
$k_{ENO,EQ}$	4.7	[-]	(Noor *et al*, 2013)
$k_{ENO,3PG}$	1.94	[µmol/gDW]	(Watabe & Freese, 1979)
$k_{ENO,PEP}$	0.177	[µmol/gDW]	(Spring & Wold, 1971)

PYK reaction

$$\mathrm{PEP} + \mathrm{ADP} \leftrightarrow \mathrm{PYR} + \mathrm{ATP}$$

It has been observed that the pyruvate kinase is allosterically activated by FBP, and inhibited by ATP (Iwakura *et al*, 1979; Somani *et al*, 1977; Wohl & Markus, 1972) . The reaction is modeled using a common modular convenience kinetics (Liebermeister *et al*, 2010).

$$v_{PYK} = v_{max,PYK} \cdot \left(\frac{[\mathrm{FBP}]}{[\mathrm{FBP}] + k_{PYK,a,FBP}}\right)^{n_{PYK,a,FBP}} \cdot \left(\frac{k_{PYK,i,ATP}}{[\mathrm{ATP}] + k_{PYK,i,ATP}}\right) \cdot \left(\frac{\frac{[\mathrm{PEP}]}{k_{PYK,PEP}}^{n_{PYK,PEP}} \cdot \frac{[\mathrm{ADP}]}{k_{PYK,ADP}} \cdot \left(1 - \frac{\frac{[\mathrm{PYR}] \cdot [\mathrm{ATP}]}{[\mathrm{PEP}]^{n_{PYK,PEP}} [\mathrm{ADP}]}}{k_{PYK,EQ}}\right)}{\left(1 + \frac{[\mathrm{PEP}]}{k_{PYK,PEP}}\right)^{n_{PYK,PEP}} \cdot \left(1 + \frac{[\mathrm{ADP}]}{k_{PYK,ADP}}\right) + \left(1 + \frac{[\mathrm{PYR}]}{k_{PYK,PYR}}\right) \cdot \left(1 + \frac{[\mathrm{ATP}]}{k_{PYK,ATP}}\right) - 1}\right)$$

Parameter	Value	Unit	Reference
$v_{max,PYK}$	8.44	[µmol/gDW/s]	**
$k_{PYK,EQ}$	24000	[-]	(Noor *et al*, 2013)
$k_{PYK,PEP}$	0.177	[µmol/gDW]	(Peskov *et al*, 2012)
$n_{PYK,PEP}$	1.05	[-]	(Speranza *et al*, 1990)
$k_{PYK,ADP}$	0.0027	[µmol/gDW]	**
$k_{PYK,PYR}$	0.3154	[µmol/gDW]	**
$k_{PYK,ATP}$	0.5357	[µmol/gDW]	**
$k_{PYK,a,FBP}$	5.28	[µmol/gDW]	**
$k_{PYK,i,ATP}$	22.5	[µmol/gDW]	(Chassagnole *et al*, 2002)
$n_{PYK,a,FBP}$	1.3	[-]	**

PFL reaction

$$\mathrm{PYR} + \mathrm{CoA} \leftrightarrow \mathrm{FOR} + \mathrm{AcCoA}$$

The PFL reaction is present in *E. coli* only under anaerobic conditions and is a key fermentation reaction. The reaction is modeled using Ping-Pong kinetics (as formerly used in (Knappe & Sawers, 1990)).

$$v_{PFL} = v_{max,PFL} \cdot \frac{\left(\frac{[\mathrm{PYR}] \cdot [\mathrm{CoA}]}{k_{PFL,CoA} \cdot k_{PFL,PYR}} \cdot \left(1 - \frac{\frac{[\mathrm{FOR}] \cdot [\mathrm{AcCoA}]}{[\mathrm{PYR}] \cdot [\mathrm{CoA}]}}{k_{PFL,EQ}}\right)\right)}{D_{PFL}}$$

$$D_{PFL} = \frac{[\mathrm{PYR}] \cdot [\mathrm{CoA}]}{k_{PFL,CoA} \cdot k_{PFL,PYR}} + \frac{[\mathrm{PYR}]}{k_{PFL,PYR}} + \frac{[\mathrm{CoA}]}{k_{PFL,CoA}} \cdot \left(1 + \frac{[\mathrm{AcCoA}]}{k_{PFL,i,AcCoA}}\right) + \frac{1}{k_{PFL,AcCoA} \cdot k_{PFL,FOR}} \cdot \left(k_{PFL,AcCoA} \cdot [\mathrm{FOR}] \cdot \left(1 + \frac{[\mathrm{PYR}]}{k_{PFL,i,PYR}}\right) + [\mathrm{AcCoA}] \cdot \left(k_{PFL,FOR} + [\mathrm{FOR}]\right)\right)$$

Parameter	Value	Unit	Reference
$v_{max,PFL}$	15.01	[µmol/gDW/s]	**
$k_{PFL,EQ}$	3000	[-]	(Noor *et al*, 2013)
$k_{PFL,PYR}$	0.4518	[µmol/gDW]	**

$k_{PFL,CoA}$	0.0124	[µmol/gDW]	(Knappe *et al*, 1974)
$k_{PFL,AcCoA\cdot}$	0.0903	[µmol/gDW]	(Knappe *et al*, 1974)
$k_{PFL,FOR}$	43.36	[µmol/gDW]	(Cintolesi *et al*, 2012)
$k_{PFL,i,AcCoA}$	1.51	[µmol/gDW]	**
$k_{PFL,i,PYR}$	1	[µmol/gDW]	**

PTACK reaction

$$\mathrm{AcCoA} + \mathrm{ADP} \leftrightarrow \mathrm{ACE} + \mathrm{ATP} + \mathrm{CoA}$$

The PTACK reaction lumps together the phosphate acetyltransferase and acetate kinase reactions and is modeled using a direct binding convenience kinetics (Liebermeister *et al*, 2010). As reported in (Campos-Bermudez *et al*, 2010), PEP and PYR are activators for the reaction and ATP acts as inhibitor.

$$v_{PTACK} = v_{max,PTACK} \cdot \left(\frac{[\mathrm{PYR}]}{k_{PTACK,PYR} + [\mathrm{PYR}]}\right) \cdot \left(\frac{[\mathrm{PEP}]}{k_{PTACK,PEP} + [\mathrm{PEP}]}\right) \cdot \left(\frac{k_{PTACK,i,ATP}}{k_{PTACK,i,ATP} + [\mathrm{ATP}]}\right) \cdot \left(\frac{\left(\frac{[\mathrm{AcCoA}]}{k_{PTACK,AcCoA}}\right)^{n_{PTACK,AcCoA}} \cdot \frac{[\mathrm{ADP}]}{k_{PTACK,ADP}} \left(1 - \frac{\frac{[\mathrm{ACEin}] \cdot [\mathrm{CoA}] \cdot [\mathrm{ATP}]}{[\mathrm{AcCoA}]^{n_{PTACK,AcCoA}} \cdot [\mathrm{ADP}]}}{k_{PTACK,EQ}}\right)}{\left(1 + \frac{[\mathrm{AcCoA}]}{k_{PTACK,AcCoA}}\right)^{n_{PTACK,AcCoA}} \cdot \left(1 + \frac{[\mathrm{ADP}]}{k_{PTACK,ADP}}\right) + \left(1 + \frac{[\mathrm{ATP}]}{k_{PTACK,ATP}}\right) \cdot \left(1 + \frac{[\mathrm{CoA}]}{k_{PTACK,CoA}}\right) \cdot \left(1 + \frac{[\mathrm{ACEin}]}{k_{PTACK,ACEin}}\right) - 1}\right)$$

Parameter	Value	Unit	Reference
$v_{max,PTACK}$	23.53	[µmol/gDW/s]	**
$k_{PTACK,EQ}$	4790	[-]	(Murabito *et al*, 2014)
$k_{PTACK,AcCoA}$	0.0794	[µmol/gDW]	(Campos-Bermudez *et al*, 2010)
$k_{PTACK,ADP}$	0.3161	[µmol/gDW]	**
$k_{PTACK,ATP}$	0.1239	[µmol/gDW]	(Fox & Roseman, 1986)
$k_{PTACK,CoA}$	0.118	[µmol/gDW]	(Campos-Bermudez *et al*, 2010)
$k_{PTACK,i,ATP}$	4.56	[µmol/gDW]	**
$k_{PTACK,PYR}$	0.0261	[µmol/gDW]	**
$k_{PTACK,PEP}$	0.25	[µmol/gDW]	** in accordance with the value of 0.5 mM of (Campos-Bermudez *et al*, 2010)
$n_{PTACK,AcCoA}$	4	[-]	**
$k_{PTACK,ACEin}$	12.39	[µmol/gCDW]	(Fox & Roseman, 1986)

ALDH reaction

$$\mathrm{AcCoA} + \mathrm{NADH} \leftrightarrow \mathrm{ACTLD} + \mathrm{CoA} + \mathrm{NAD}$$

The acetaldehyde-CoA dehydrogenase reaction is modeled using an ordered Bi-Bi rate law (Wratten & Cleland, 1963).

$$v_{ALDH} = \frac{v_{max,ALDH} \cdot \left(\frac{[\mathrm{AcCoA}] \cdot [\mathrm{NADH}]}{k_{ALDH,i,AcCoA} \cdot k_{ALDH,NADH}} \left(1 - \frac{\frac{[\mathrm{ACTLD}] \cdot [\mathrm{CoA}]}{[\mathrm{AcCoA}] \cdot [\mathrm{NADH}]}}{k_{ALDH,EQ}} \right) \right)}{D_{ALDH}}$$

$$D_{ALDH} = 1 + \frac{[\mathrm{AcCoA}]}{k_{ALDH,i,AcCoA}} + \frac{[\mathrm{AcCoA}] \cdot [\mathrm{NADH}]}{k_{ALDH,i,AcCoA} \cdot k_{ALDH,NADH}} + \frac{[\mathrm{CoA}]}{k_{ALDH,i,CoA}} + \frac{[\mathrm{ACTLD}] \cdot [\mathrm{CoA}]}{k_{ALDH,i,CoA} \cdot k_{ALDH,ACTLD}}$$

Parameter	Value	Unit	Reference
$v_{max,ALDH}$	30	[µmol/gDW/s]	**
$k_{ALDH,EQ}$	0.273	[-]	**
$k_{ALDH,i,AcCoA}$	0.012	[µmol/gDW]	(Shone & Fromm, 1981)
$k_{ALDH,NADH}$	0.1	[µmol/gDW]	**
$k_{ALDH,i,CoA}$	0.014	[µmol/gDW]	(Cintolesi *et al*, 2012)
$k_{ALDH,ACTLD}$	17.7	[µmol/gDW]	(Shone & Fromm, 1981)

ADH reaction

$$\mathrm{ACTLD} + \mathrm{NADH} \leftrightarrow \mathrm{ETH} + \mathrm{CoA} + \mathrm{NAD}$$

The alcohol dehydrogenase reaction is modeled using an ordered Bi-Bi rate equation (Wratten & Cleland, 1963) :

$$v_{ADH} = \frac{v_{max,ADH} \cdot \left(\frac{[\mathrm{ACTLD}] \cdot [\mathrm{NADH}]}{k_{ADH,i,ACTLD} \cdot k_{ADH,NADH}} \left(1 - \frac{\frac{[\mathrm{ETH}] \cdot [\mathrm{NAD}]}{[\mathrm{ACTLD}] \cdot [\mathrm{NADH}]}}{k_{ALDH,EQ}} \right) \right)}{D_{ADH}}$$

$$D_{ADH} = 1 + \frac{[\mathrm{ACTLD}]}{k_{ADH,i,ACTLD}} + \frac{[\mathrm{ACTLD}] \cdot [\mathrm{NADH}]}{k_{ADH,i,ACTLD} \cdot k_{ADH,NADH}} + \frac{[\mathrm{NAD}]}{k_{ADH,i,NAD}} + \frac{[\mathrm{NAD}] \cdot [\mathrm{ETH}]}{k_{ADH,i,NAD} \cdot k_{ADH,ETH}}$$

Parameter	Value	Unit	Reference
$v_{max,ADH}$	18	[µmol/gDW/s]	**
$k_{ADH,EQ}$	1500	[-]	(Noor *et al*, 2013)
$k_{ADH,i,ACTLD}$	0.0531	[µmol/gDW]	(Hoefnagel *et al*, 2002)
$k_{ADH,NADH}$	0.172	[µmol/gDW]	**
$k_{ADH,i,NAD}$	0.142	[µmol/gDW]	(Matsuoka & Kurata, 2017)
$k_{ADH,ETH}$	1.77	[µmol/gDW]	(Hoefnagel *et al*, 2002)

LDH reaction

$$\mathrm{PYR} + \mathrm{NADH} \leftrightarrow \mathrm{LAC} + \mathrm{NAD}$$

The reaction is modeled using a common modular rate law (Liebermeister *et al*, 2010) including the homotropic effect of PYR and NADH reported in (Tarmy & Kaplan, 1968)..

$$v_{LDH} = v_{max,LDH} \cdot \frac{\left(\frac{[PYR]}{k_{LDH,PYR}}\right)^{n_{LDH,PYR}} \left(\frac{[NADH]}{k_{LDH,NADH}}\right)^{n_{LDH,NADH}} \left(\frac{[LACin][NAD]}{\frac{[PYR]^{n_{LDH,PYR}}[NADH]^{n_{LDH,NADH}}}{k_{LDH,EQ}}}\right)}{\left(1+\frac{[PYR]}{k_{LDH,PYR}}\right)^{n_{LDH,PYR}} \left(1+\frac{[NADH]}{k_{LDH,NADH}}\right)^{n_{LDH,NADH}} + \left(1+\frac{[NAD]}{k_{LDH,NAH}}\right)\left(1+\frac{[LACin]}{k_{LDH,LACin}}\right) - 1}$$

Parameter	Value	Unit	Reference
$v_{max,LDH}$	11	[µmol/gCDW/s]	**
$k_{LDH,PYR}$	12.74	[µmol/gCDW]	(Tarmy & Kaplan, 1968)
$n_{LDH,PYR}$	1.2	[-]	(Tarmy & Kaplan, 1968)
$k_{LDH,NADH}$	0.004	[µmol/gCDW]	**
$n_{LDH,NADH}$	1	[-]	(Tarmy & Kaplan, 1968)
$k_{LDH,EQ}$	14000	[-]	(Noor *et al*, 2013)
$k_{LDH,LAC}$	7.84	[µmol/gCDW]	**
$k_{LDH,NAD}$	0.4425	[µmol/gCDW]	(Tarmy & Kaplan, 1968)

CSICD reaction

$$AcCoA + NAD + OAA \leftrightarrow AKG + NADH + CoA + CO_2$$

This reaction lumps all steps from AcCoA to AKG (see also legend of Figure S1). Reversible common modular convenience kinetics (Liebermeister *et al*, 2010)is used to describe the reaction. The inhibition of the citrate synthase by AKG and NADH (as observed in(Robinson *et al*, 1983)) is accounted for in the kinetics.

$$v_{CSICD} = v_{max,CSICD} \cdot \frac{\frac{[AcCoA]}{k_{CSICD,AcCoA}} \cdot \frac{[OAA]}{k_{CSICD,OAA}} \frac{[NAD]}{k_{CSICD,NAD}} \left(1 - \frac{[AKG][CoA][NADH][CO_2]}{[AcCoA][NAD][OAA]\ k_{CSICD,EQ}}\right)}{D_{CSICD}} \cdot \left(\frac{k_{CSICD,i,NADH}}{k_{CSICD,i,NADH} + [NADH]}\right) \cdot \left(\frac{k_{CSICD,i,AKG}}{k_{CSICD,i,AKG} + [AKG]}\right)^{n_{CSICD,i,AKG}}$$

$$D_{CSICD} = \left(\left(1 + \frac{[AcCoA]}{k_{CSICD,AcCoA}}\right) \cdot \left(1 + \frac{[OAA]}{k_{CSICD,OAA}}\right) \cdot \left(1 + \frac{[NAD]}{k_{CSICD,NAD}}\right) + \left(1 + \frac{[AKG]}{k_{CSICD,AKG}}\right) \cdot \left(1 + \frac{[NADH]}{k_{CSICD,NADH}}\right) \cdot \left(1 + \frac{[CoA]}{k_{CSICD,CoA}}\right) \cdot \left(1 + \frac{[CO_2]}{k_{CSICD,CO2}}\right) - 1\right)$$

Parameter	Value	Unit	Reference
$v_{max,CSICD}$	1.956	[µmol/gDW/s]	**
$k_{CSICD,EQ}$	6.2	[µmol/gDW]	**
$k_{CSICD,AcCoA}$	0.2124	[µmol/gDW]	(Anderson & Duckworth, 1988)
$k_{CSICD,NAD}$	0.067	[µmol/gDW]	**
$k_{CSICD,OAA}$	0.046	[µmol/gDW]	**
$k_{CSICD,AKG}$	0.628	[µmol/gDW]	(Kotte *et al*, 2010)

$n_{CSICD,AKG}$	4	[-]	**
$k_{CSICD,i,NADH}$	0.7	[µmol/gDW]	(Danson & Weitzman, 1973)
$k_{CSICD,NADH}$	0.7	[µmol/gDW]	**
$k_{CSICD,CoA}$	1.53	[µmol/gDW]	**
$k_{CSICD,AKG}$	0.628	[µmol/gDW]	**
$k_{CSICD,CO2}$	2.87	[µmol/gDW]	**

PCK reaction

$$\mathrm{OAA} + \mathrm{ATP} \leftrightarrow \mathrm{PEP} + \mathrm{CO_2} + \mathrm{ADP}$$

This reaction catalyzes the carboxylation of oxaloacetate to PEP under consumption of ATP. Under particular conditions, the reaction can also run in the reverse direction (Zhang *et al*, 2009) accompanied with the production of ATP. A simplified Random Bi Ter mechanism is used to model the kinetics as in (Peskov *et al*, 2012):

$$v_{PCK} = \frac{v_{max,PCK} \cdot \left(\frac{[\mathrm{OAA}] \cdot [\mathrm{ATP}]}{k_{PCK,OAA} \cdot k_{PCK,ATP}} \cdot \left(1 - \frac{\frac{[\mathrm{PEP}] \cdot [\mathrm{CO_2}] \cdot [\mathrm{ADP}]}{[\mathrm{OAA}] \cdot [\mathrm{ATP}]}}{k_{PCK,EQ}} \right) \right)}{D_{PCK}}$$

$$D_{PCK} = 1 + \frac{[\mathrm{ATP}]}{k_{PCK,ATP}} + \frac{[\mathrm{OAA}]}{k_{PCK,OAA}} + \frac{[\mathrm{OAA}] \cdot [\mathrm{ATP}]}{k_{PCK,OAA} \cdot k_{PCK,ATP}} + \left(\frac{[\mathrm{CO_2}]}{k_{PCK,CO_2}} + \frac{[\mathrm{PEP}]}{k_{PCK,PEP}} + \frac{[\mathrm{ADP}]}{k_{PCK,ADP}} + \frac{[\mathrm{PEP}] \cdot [\mathrm{CO_2}]}{k_{PCK,PEP} \cdot k_{PCK,CO_2}} + \frac{[\mathrm{CO_2}] \cdot [\mathrm{ADP}]}{k_{PCK,CO_2} \cdot k_{PCK,ADP}} + \frac{[\mathrm{PEP}] \cdot [\mathrm{ADP}]}{k_{PCK,PEP} \cdot k_{PCK,ADP}} + \frac{[\mathrm{PEP}] \cdot [\mathrm{ADP}] \cdot [\mathrm{CO_2}]}{k_{PCK,PEP} \cdot k_{PCK,ADP} \cdot k_{PCK,CO_2}} \right)$$

Parameter	Value	Unit	Reference
$v_{max,PCK}$	0.0513	[µmol/gDW/s]	**
$k_{PCK,EQ}$	1.88	[µmol/gDW]	(Peskov *et al*, 2012)
$k_{PCK,OAA}$	1.18	[µmol/gDW]	(Krebs & Bridger, 1980)
$k_{PCK,ATP}$	0.1062	[µmol/gDW]	(Krebs & Bridger, 1980)
k_{PCK,CO_2}	2.571	[µmol/gDW]	**
$k_{PCK,PEP}$	0.885	[µmol/gDW]	(Krebs & Bridger, 1980)
$k_{PCK,ADP}$	0.0885	[µmol/gDW]	(Krebs & Bridger, 1980)
$[\mathrm{CO_2}]$	2.478	[µmol/gDW]	(Millard *et al*, 2017)

PPC reaction

$$\mathrm{PEP} + \mathrm{CO_2} \leftrightarrow \mathrm{OAA}$$

The anaplerotic PPC reaction catalyzes the carboxylation of PEP into OAA. A sigmoidal response to PEP has been observed (Wohl & Markus, 1972), and the reaction is modeled using direct binding convenience kinetics (Liebermeister *et al*, 2010). Activation from ACoA and FBP ((Izui *et al*, 1981)) and inhibition of by FUM ((Yano & Izui, 1997)) are take into account.

$$r_{PPC} = v_{max,PPC} \cdot \frac{\left(\frac{[\mathrm{PEP}]}{k_{PPC,PEP}}\right)^{n_{PPC,PEP}} \cdot \frac{[\mathrm{CO_2}]}{k_{PPC,CO_2}} \cdot \left(1 - \frac{\frac{[\mathrm{OAA}]}{[\mathrm{PEP}]^{n_{PPC,PEP}}[\mathrm{CO_2}]}}{k_{PPC,EQ}}\right)}{\left(1 + \left(\frac{[\mathrm{PEP}]}{k_{PPC,PEP}}\right)^{n_{PPC,PEP}} \cdot \frac{[\mathrm{CO_2}]}{k_{PPC,CO_2}} + \frac{[\mathrm{OAA}]}{k_{PPC,OAA}}\right)} \cdot \left(\frac{[\mathrm{AcCoA}]}{k_{PPC,a,AcCoA} + [\mathrm{AcCoA}]}\right)^{n_{PPC,a,AcCoA}} \cdot \left(\frac{[\mathrm{FBP}]}{k_{PPC,a,FBP} + [\mathrm{FBP}]}\right) \cdot \left(\frac{k_{PPC,i,FUM}}{k_{PPC,i,FUM} + [\mathrm{FUM}]}\right)$$

Parameter	Value	Unit	Reference
$v_{max,PPC}$	4.087	[µmol/gCDW/s]	**
$k_{PPC,PEP}$	0.2655	[µmol/gCDW]	
$k_{PPC,EQ}$	2001	[-]	(Noor *et al*, 2013)
k_{PPC,CO_2}	1.09	[µmol/gCDW]	(Kai *et al*, 1999)
$k_{PPC,OAA}$	1.387	[µmol/gCDW]	**
$n_{PPC,PEP}$	4	[-]	(Peskov *et al*, 2012)
$k_{PPC,a,AcCoA}$	0.7611	[µmol/gCDW]	(Smith *et al*, 1980)
$n_{PPC,a,AcCoA}$	1.4	[-]	(Smith *et al*, 1980)
$k_{PPC,a,FBP}$	5.98	[µmol/gCDW]	(Smith *et al*, 1980)
$k_{PPC,i,FUM}$	0.2124	[µmol/gCDW]	(Yano & Izui, 1997)
CO_2	2.478	[µmol/gCDW]	(Millard *et al*, 2017)

MDH reaction

$$\mathrm{OAA} + \mathrm{NADH} \leftrightarrow \mathrm{FUM} + \mathrm{NAD}$$

This reaction describes the left branch of the TCA cycle under anaerobic conditions, lumping together the malate dehydrogenase and fumarase reactions. The reaction is modeled using ordered Bi-Bi reaction law as observed in (Wright *et al*, 1995).

$$v_{MDH} = \frac{v_{max,MDH} \cdot \frac{[\mathrm{OAA}] \cdot [\mathrm{NADH}]}{k_{MDH,i,OAA} \cdot k_{MDH,NADH}}\left(1 - \frac{\frac{[\mathrm{FUM}] \cdot [\mathrm{NAD}]}{[\mathrm{OAA}] \cdot [\mathrm{NADH}]}}{k_{MDH,EQ}}\right)}{D_{MDH}}$$

$$D_{MDH} = 1 + \frac{[\mathrm{NADH}] \cdot k_{MDH,OAA}}{k_{MDH,i,OAA} \cdot k_{MDH,NADH}} + \frac{[\mathrm{OAA}]}{k_{MDH,i,OAA}} + \frac{[\mathrm{OAA}] \cdot [\mathrm{NADH}]}{k_{MDH,i,OAA} \cdot k_{MDH,NADH}} + \frac{k_{MDH,NAD} \cdot [\mathrm{OAA}] \cdot [\mathrm{FUM}]}{k_{MDH,FUM} \cdot k_{MDH,i,NAD} \cdot k_{MDH,i,OAA}} + \frac{[\mathrm{NAD}] \cdot [\mathrm{FUM}]}{k_{MDH,MAL} \cdot k_{MDH,i,NAD}} + \frac{[\mathrm{NADH}] \cdot [\mathrm{NAD}] \cdot [\mathrm{FUM}]}{k_{MDH,FUM} \cdot k_{MDH,i,NAD} \cdot k_{MDHi,NADH}} + \frac{[\mathrm{OAA}] \cdot [\mathrm{NADH}] \cdot [\mathrm{FUM}]}{k_{MDH,i,FUM} \cdot k_{MDH,i,OAA} \cdot k_{MDH,NADH}} + \frac{k_{MDH,OAA} \cdot [\mathrm{NADH}] \cdot [\mathrm{NAD}]}{k_{MDH,i,NAD} \cdot k_{MDH,i,OAA} \cdot k_{MDH,NADH}} + \frac{k_{MDH,NAD} \cdot [\mathrm{FUM}]}{k_{MDH,FUM} \cdot k_{MDH,i,NAD}} + \frac{[\mathrm{NAD}]}{k_{MDH,i,NAD}}$$

Parameter	Value	Unit	Reference
$v_{max,MDH}$	2.75	[µmol/gCDW/s]	**
$k_{MDH,EQ}$	4.4e+04	[-]	(Noor *et al*, 2013)

$k_{MDH,OAA}$	0.09912	[µmol/gCDW]	(Kim *et al*, 1999)
$k_{MDH,NADH}$	0.10797	[µmol/gCDW]	(Muslin *et al*, 1995)
$k_{MDH,FUM}$	0.1699	[µmol/gCDW]	(Vorobieva *et al*, 2014)
$k_{MDH,NAD}$	0.16638	[µmol/gCDW]	(Vorobieva *et al*, 2014)
$k_{MDH,i,OAA}$	0.0354	[µmol/gCDW]	(Wright *et al*, 1995)
$k_{MDH,i,NAD}$	0.041	[µmol/gCDW]	(Millard *et al*, 2017)
$k_{MDH,i,FUM}$	0.8	[µmol/gCDW]	**
$k_{MDH,i,NADH}$	0.02832	[µmol/gCDW]	(Wright *et al*, 1995)

FRD reaction

$$\mathrm{FUM} + \mathrm{NADH} + 0.75\ \mathrm{ADP} \leftrightarrow \mathrm{SUC} + \mathrm{NAD} + 0.75\ \mathrm{ATP}$$

This lumped reaction describes ATP synthesis via fumarate reduction. The first step is the conversion of fumarate into succinate via fumarate reductase in which menaquinol is oxidized to menaquinone. In *E. coli*, menaquinol is regenerated by the oxidation of NADH via an NADH dehydrogenase that in turn is linked to proton translocation from cytoplasm to periplasm which then enables ATP production via ATP synthase. In the cumulated stoichiometry of this reaction in the model, the menaquinol pool is not explicitly modeled and the effects on NADH and ATP directly taken into account. The reaction is modeled using Michaelis-Menten kinetics. As reported in (Maklashina *et al*, 2006), OAA acts as an inhibitor for the reaction.

$$r_{FRD} = v_{max,FRD} \cdot \frac{\frac{[\mathrm{FUM}]}{k_{FRD,FUM}} \cdot \frac{[\mathrm{NADH}]}{k_{FRD,NADH}} \cdot \left(\frac{[\mathrm{ADP}]}{k_{FRD,ADP}}\right)^{0.75} \cdot \left(1 - \frac{\frac{[\mathrm{SUC}][\mathrm{NAD}]\,[\mathrm{ATP}]^{0.75}}{[\mathrm{FUM}][\mathrm{NADH}]\,[\mathrm{ADP}]^{0.75}}}{k_{FRD,EQ}}\right)}{D_{FRD}}$$

$$D_{FRD} = \left(1 + \frac{[\mathrm{FUM}]}{k_{FRD,FUM}}\right) \cdot \left(1 + \frac{[\mathrm{NADH}]}{k_{FRD,NADH}}\right) \cdot \left(1 + \frac{[\mathrm{ADP}]}{k_{FRD,ADP}}\right)^{0.75} + \left(1 + \frac{[\mathrm{SUC}]}{k_{FRD,SUC}}\right) \cdot \left(1 + \frac{[\mathrm{NAD}]}{k_{FRD,NAD}}\right) \cdot \left(1 + \frac{[\mathrm{ATP}]}{k_{FRD,ATP}}\right)^{0.75} + \frac{[\mathrm{OAA}]}{k_{FRD,i,OAA}} - 1$$

Parameter	Value	Unit	Reference
$v_{max,FRD}$	2.89	[µmol/gDW/s]	**
$k_{FRD,EQ}$	120	[-]	(Noor *et al*, 2013)
$k_{FRD,FUM}$	0.0354	[µmol/gDW]	(Maklashina *et al*, 2006)
$k_{FRD,NADH}$	0.02	[µmol/gDW]	**
$k_{FRD,ADP}$	0.1429	[µmol/gDW]	**
$k_{FRD,OAA}$	2.63	[µmol/gDW]	**
$k_{FRD,SUC}$	1.45	[µmol/gDW]	**
$k_{FRD,NAD}$	0.007	[µmol/gDW]	**
$k_{FRD,ATP}$	0.84	[µmol/gDW]	**

NGAM reaction

$$\mathrm{ATP} \rightarrow \mathrm{ADP}$$

The NGAM reaction accounts for the consumption of ATP for non-growth associated maintenance processes. The reaction is modeled as a demand reaction by using a hill kinetics and a small K_m

value for ATP to ensure that ATP is provided for NGAM also under low ATP concentration. The v_{max} has been chosen to yield a minimum non-growth associated maintenance flux for all strains similar to the one determined experimentally and close to 3.15 mmol (gDW h)$^{-1}$, a value that is often used as reference for NGAM demand of ATP in *E. coli* (Orth *et al*, 2011).

$$v_{NGAM} = v_{max,NGAM} \cdot \frac{[\text{ATP}]^{\,n_{NGAM}}}{k_{NGAM,ATP}^{n_{NGAM}} + [\text{ATP}]^{\,n_{NGAM}}}$$

Parameter	Value	Unit	Reference
$v_{max,NGAM}$	1.2	[µmol/gDW/s]	**
$k_{NGAM,ATP}$	0.008	[µmol/gDW]	**
n_{NGAM}	8	[-]	**

ATPase reaction

$$\text{ATP} \rightarrow \text{ADP}$$

The ATPase reaction describes the hydrolysis of ATP into ADP via the F_1-ATPase (the genes of the F_1-ATPase is overexpressed in LC, MC and HC strains; see main text). When simulating the effect of increasing levels of ATPase in the cell, the $v_{max,ATPase}$ is increased accordingly.

$$v_{ATPase} = v_{max,ATPase} \cdot \frac{[\text{ATP}]^{\,n_{ATPase}}}{k_{ATPase,ATP}^{n_{ATPase}} + [\text{ATP}]^{\,n_{ATPase}}}$$

Parameter	Value	Unit	Reference
$v_{max,ATPase}$	0 – 25 (variable)	[µmol/gDW/s]	**
$k_{ATPase,ATP}$	0.48	[µmol/gDW]	**
n_{ATPase}	2.2	[-]	**

ADK reaction

$$\text{AMP} + \text{ATP} \leftrightarrow 2\ \text{ADP}$$

The adenylate kinase reaction accounts for the conversion of AMP and ATP into (2) ADP and is modeled using Michaelis-Menten kinetics.

$$v_{ADK} = v_{max,ADK} \cdot \frac{\frac{[\text{ATP}]}{k_{ADK,ATP}} \cdot \frac{[\text{AMP}]}{k_{ADK,AMP}} \cdot \left(1 - \frac{\frac{[\text{ADP}]^2}{[\text{ATP}][\text{AMP}]}}{k_{ADK,eq}}\right)}{1 + \frac{[\text{ATP}]}{k_{ADK,ATP}} + \frac{[\text{AMP}]}{k_{ADK,AMP}} + \frac{[\text{ADP}]}{k_{ADK,ADP}}}$$

Parameter	Value	Unit	Reference
$v_{max,ADK}$	14	[µmol/gCDW/s]	**
$k_{ADK,eq}$	0.9	[-]	(Noor *et al*, 2013)
$k_{ADK,ATP}$	0.87	[µmol/gCDW]	**
$k_{ADK,AMP}$	0.25	[µmol/gCDW]	**
$k_{ADK,ADP}$	0.08	[µmol/gCDW]	**

Growth reaction

A biomass synthesis reaction was included in the model to account for growth. This pseudo reaction consumes intracellular precursors (here: PEP, PYR, GAP, ATP, AcCoA, AKG, OAA, NADH, F6P, G6P, 3PG) with the following stoichiometries (the metabolite stoichiometries (except biomass) are given in µmol):

$$\begin{aligned} 459.27\ \mathrm{GAP} + 810.19\ \mathrm{PEP} + 2784.13\ \mathrm{PYR} + 53107.60\ \mathrm{ATP} + 3856.57\ \mathrm{AcCoA} + 1075.32\ \mathrm{AKG} \\ + 2924.97\ \mathrm{OAA} + 14889.30\ \mathrm{NADH} + 466.42\ \mathrm{F6P} + 600.28\ \mathrm{G6P} + 1717.48\ \mathrm{3PG} \\ + 107.95\ \mathrm{FOR} \\ \rightarrow 1\ gram\ \mathrm{Biomass} + 581.01\ \mathrm{ACE} + 2334.41\ \mathrm{CO_2} + 1040.71\ \mathrm{SUC} + 14889.30\ \mathrm{NAD} \\ + 3856.57\ \mathrm{CoA} + 49739.50\ \mathrm{ADP} + 3368.10\ \mathrm{AMP} \end{aligned}$$

The stoichiometry was obtained by reducing a genome-scale stoichiometric model of *E coli* (iJO1366(Orth *et al*, 2011)) to the core metabolic network shown in Figure S1 using the NetworkReducer package of *CellNetAnalyzer* (Erdrich *et al*, 2015; Klamt *et al*, 2007). The stoichiometry obtained with this reduction method accounts implicitly for all carbon precursors and cofactors from the core model needed to build the original building blocks (e.g. amino acids, nucleotides etc.) of the biomass in the genome-scale model. For example, 0.51 mmol alanine is needed (among many other building blocks) in *i*JO1366 for building 1 gram of biomass. Since alanine is synthesized by transamination of pyruvate (pyruvate + glutamate → alanine + oxoglutarate) and since recycling of glutamate from oxoglutarate consumes one NADPH (and one ammonia molecule not considered herein), alanine synthesis is accounted for in the reduced biomass synthesis stoichiometry by consumption of 0.51 mmol pyruvate and 0.51 mmol NADH (as was described earlier, in our model, NADPH is included in the NADH pool). As was mentioned in section 1.1, the cumulated biomass stoichiometry also accounts for consumption of the precursors erythrose-4-phosphate and ribose-5-phosphate in the PPP.
For the kinetics of the growth rate we use a Monod kinetics term for each precursor and cofactor consumed in the biomass stoichiometry given above. The requirement of ATP for growth has been given a higher weight by using a hill kinetics and μ_{max} represents the maximum growth rate (here with unit [1/s]).

$$\begin{aligned} \mu = \mu_{max} \cdot \frac{[\mathrm{F6P}]}{k_{g,F6P} + [\mathrm{F6P}]} \cdot \frac{[\mathrm{GAP}]}{k_{g,GAP} + [\mathrm{GAP}]} \cdot \frac{[\mathrm{PEP}]}{k_{g,PEP} + [\mathrm{PEP}]} \cdot \frac{[\mathrm{PYR}]}{k_{g,PYR} + [\mathrm{PYR}]} \cdot \\ \frac{[\mathrm{ATP}]^{n_{g,ATP}}}{k_{g,ATP}^{n_{g,ATP}} + [\mathrm{ATP}]^{n_{g,ATP}}} \cdot \frac{[\mathrm{AcCoA}]}{k_{g,AcCoA} + [\mathrm{AcCoA}]} \cdot \frac{[\mathrm{AKG}]}{k_{g,AKG} + [\mathrm{AKG}]} \cdot \frac{[\mathrm{OAA}]}{k_{g,OAA} + [\mathrm{OAA}]} \cdot \\ \frac{[\mathrm{NADH}]}{k_{g,NADH} + [\mathrm{NADH}]} \cdot \frac{[\mathrm{G6P}]}{k_{g,G6P} + [\mathrm{G6P}]} \cdot \frac{[\mathrm{3PG}]}{k_{g,3PG} + [\mathrm{3PG}]} \cdot \frac{[\mathrm{FOR}]}{k_{g,FOR} + [\mathrm{FOR}]} \end{aligned}$$

Parameter	Value	Unit	Reference
μ_{max}	2.074e-04	[1/s]	**
$k_{g,F6P}$	0.015	[µmol/gDW]	**
$k_{g,GAP}$	0.0021	[µmol/gDW]	**
$k_{g,PEP}$	0.0075	[µmol/gDW]	**
$k_{g,PYR}$	0.007	[µmol/gDW]	**
$k_{g,ATP}$	1.43	[µmol/gDW]	**
$k_{g,AcCoA}$	0.0043	[µmol/gDW]	**
$k_{g,AKG}$	0.00143	[µmol/gDW]	**
$k_{g,OAA}$	0.009	[µmol/gDW]	**
$k_{g,NADH}$	7.2e-04	[µmol/gDW]	**

$k_{g,3PG}$	0.0018	[µmol/gDW]	**
$k_{g,G6P}$	0.075	[µmol/gDW]	**
$k_{g,FOR}$	0.0018	[µmol/gDW]	**

Export reactions

The reactions describing the export of FOR, LAC, ACE, ETH, PYR and SUC between the cytoplasm and the external environment are modeled using Michaelis-Menten kinetics. The parameters values were chosen to ensure that the transport reactions are no limiting steps to the fermentative fluxes (similarly to (Millard *et al*, 2017)).

Formate transport

$$\mathrm{FOR} \rightarrow \mathrm{FOR}_{ex}$$

$$v_{FOR_{transport}} = v_{max,FOR_{transport}} \frac{[\mathrm{FOR}]}{\left([\mathrm{FOR}] + k_{FOR_{transport}}\right)}$$

Parameter	Value	Unit	Reference
$v_{max,FOR_{transport}}$	100	[µmol/gDW/s]	**
$k_{FOR_{transport}}$	10	[µmol/gDW]	**

Lactate transport

$$\mathrm{LAC} \rightarrow \mathrm{LAC}_{ex}$$

$$v_{LAC_{transport}} = v_{max,LAC_{transport}} \frac{[\mathrm{LAC}]}{\left([\mathrm{LAC}] + k_{LAC_{transport}}\right)}$$

Parameter	Value	Unit	Reference
$v_{max,LAC_{transport},}$	100	[µmol/gDW/s]	**
$k_{LAC_{transport}}$	10	[µmol/gDW]	**

Acetate transport

$$\mathrm{ACE} \rightarrow \mathrm{ACE_{ex}}$$

$$v_{ACE_{transport}} = v_{max,ACE_{transport}} \frac{[\mathrm{ACE}]}{\left([\mathrm{ACE}] + k_{ACE_{transport}}\right)}$$

Parameter	Value	Unit	Reference
$v_{max,ACE_{transport}}$	100	[µmol/gDW/s]	**
$k_{ACE_{transport}}$	10	[µmol/gDW]	**

Ethanol transport

$$\mathrm{ETH} \rightarrow \mathrm{ETH}_{ex}$$

$$v_{ETH_{transport}} = v_{max,ETH_{transport}} \frac{[\mathrm{ETH}]}{\left([\mathrm{ETH}] + k_{ETH_{transport}}\right)}$$

Parameter	Value	Unit	Reference

$v_{max,ETH_{transport}}$	100	[µmol/gDW/s]	**
$k_{ETH_{transport}}$	10	[µmol/gDW]	**

Succinate transport

$$\mathrm{SUC} \rightarrow \mathrm{SUC}_{ex}$$

$$v_{SUC_{transport}} = v_{max,SUC_{transport}} \cdot \frac{[\mathrm{SUC}]}{[\mathrm{SUC}] + k_{SUC_{transport}}}$$

Parameter	Value	Unit	Reference
$v_{SUC_{transport},max}$	100	[µmol/gDW/s]	**
$k_{SUC_{transport}}$	10	[µmol/gDW]	**

Fumarate uptake

This reaction was set inactive in all simulations except for a single case, where we tested the effect of supplying fumarate as additional substrate under ATPase HC conditions (see Discussion section in the main text). The reaction is modeled using reversible Michaelis-Menten.

$$\mathrm{FUM}_{ex} \leftrightarrow \mathrm{FUM}$$

$$v_{FUM_{transport}} = v_{max,FUM_{uptake}} \frac{\frac{[\mathrm{FUM}_{ex}]}{k_{FUM_{uptake},FUMex}} \cdot \left(1 - \frac{\frac{[\mathrm{FUM}]}{[\mathrm{FUM}_{ex}]}}{k_{FUM_{uptake},EQ}}\right)}{\left(1 + \frac{[\mathrm{FUM}_{ex}]}{k_{FUM_{uptake},FUMex}} + \frac{[\mathrm{FUM}]}{k_{FUM_{uptake},FUM}}\right)}$$

Parameter	Value	Unit	Reference
$v_{max,FUM_{uptake}}$	0 - 10	[µmol/gDW/s]	**
$k_{FUM_{uptake},FUMex}$	0.4	[g/l]	**
$k_{FUM_{uptake},FUM}$	1	[µmol/gDW]	**
$k_{FUM_{uptake},EQ}$	1	[-]	**
$[\mathrm{FUM}_{ex}]$	1	[g/l]	**

1.4 Simulations and reference points

For a direct comparison of simulated and measured rates and concentrations for the different ATPase strains (Figures 5 and 6A in the main document) we determined, for a given strain, the (reference) $v_{max,ATPase}$ value such that the simulated and the measured (steady-state) glucose uptake rate in the respective ATPase strain was identical. This reference point was then chosen to compare the other measured fluxes and the metabolite concentrations for this strain. The determined reference points are as follows for model version 1 (cf. Figure 5 and 6A in main manuscript): (1) WT strain: $v_{max,ATPase}$ = 0 mmol/(gDW h); (2) LC ATPase strain : $v_{max,ATPase}$ = 9.15 mmol/(gDW h); (3) MC ATPase strain: $v_{max,ATPase}$ = 27.65 mmol/(gDW h); and (4) HC ATPase strain: $v_{max,ATPase}$ = 81.22 mmol/(gDW h).

2. Kinetic model version 2

As described in the main text, the first version of the model was able to reproduce the biphasic behavior of the measured exchange fluxes. However, as shown in Figure 5 in the main document, the model failed in predicting major trends of the intracellular metabolite concentrations (in particular, of FBP, PEP and PYR) and almost no lactate was produced by ATPase HC. To overcome this issue and for the reasons reported in the main text, few modifications have been introduced in model version 2, which are described below. Afterwards, metabolic control analysis was performed with model version 2 to identify potential bottleneck reactions for the glycolytic flux.

2.1 Modified rate laws and parameters

In the following we describe only changes in the kinetic rate laws and parameters introduced in model version 2 (changes are indicated in bold and with gray background). All other rate laws, ODEs and parameters are identical to model version 1.

PFL reaction: introduction of a regulation term

A regulation term is introduced for PFL to ensure that it is only fully active (or expressed) under conditions with sufficiently high ATP concentrations.

$$\mathrm{PYR} + \mathrm{CoA} \leftrightarrow \mathrm{FOR} + \mathrm{AcCoA}$$

$$v_{PFL,modified} = \frac{[\mathbf{ATP}]^{n_{rf}}}{[\mathbf{ATP}]^{n_{rf}} + \boldsymbol{k_{rf}^{n_{rf}}}} \cdot v_{max,PFL} \cdot \frac{\left(\frac{[\mathrm{PYR}] \cdot [\mathrm{CoA}]}{k_{PFL,CoA} \cdot k_{PFL,PYR}} \cdot \left(1 - \frac{\frac{[\mathrm{FOR}] \cdot [\mathrm{AcCoA}]}{[\mathrm{PYR}] \cdot [\mathrm{CoA}]}}{k_{PFL,EQ}} \right) \right)}{D_{PFL}}$$

$$D_{PFL} = \frac{[\mathrm{PYR}] \cdot [\mathrm{CoA}]}{k_{PFL,CoA} \cdot k_{PFL,PYR}} + \frac{[\mathrm{PYR}]}{k_{PFL,PYR}} + \frac{[\mathrm{CoA}]}{k_{PFL,CoA}} \cdot \left(1 + \frac{[\mathrm{AcCoA}]}{k_{PFL,i,AcCoA}} \right) + \frac{1}{k_{PFL,AcCoA} \cdot k_{PFL,FOR}} \cdot \left(k_{PFL,AcCoA} \cdot [\mathrm{FOR}] \cdot \left(1 + \frac{[\mathrm{PYR}]}{k_{PFL,i,PYR}} \right) + [\mathrm{AcCoA}] \cdot \left(k_{PFL,FOR} + [\mathrm{FOR}] \right) \right)$$

Parameter	Value	Unit	Reference
$\boldsymbol{v_{max,PFL}}$	**20.1**	[µmol/gDW/s]	**
$k_{PFL,EQ}$	3000	[-]	(Noor *et al*, 2013)
$k_{PFL,PYR}$	0.4518	[µmol/gDW]	**
$k_{PFL,CoA}$	0.0124	[µmol/gDW]	(Knappe *et al*, 1974)
$k_{PFL,AcCoA\cdot}$	0.0903	[µmol/gDW]	(Knappe *et al*, 1974)
$k_{PFL,FOR}$	43.36	[µmol/gDW]	(Cintolesi *et al*, 2012)
$k_{PFL,i,AcCoA}$	1.51	[µmol/gDW]	**
$k_{PFL,i,PYR}$	1	[µmol/gDW]	**
$\boldsymbol{k_{rf}}$	**0. 98**	[µmol/gDW]	**
$\boldsymbol{n_{rf}}$	**3.1**	[-]	**

PYK reaction: introduction of a PYR dependent inhibition term

An inhibition term depending on the abundance of PYR has been introduced to reflect decreased activity of PYK in presence of high amounts of PYR.

$$\mathrm{PEP} + \mathrm{ADP} \leftrightarrow \mathrm{PYR} + \mathrm{ATP}$$

$$v_{PYK} = v_{max,PYK} \cdot \left(\frac{[\mathrm{FBP}]}{[\mathrm{FBP}] + k_{PYK,a,FBP}}\right)^{n_{PYK,a,FBP}} \cdot \left(\frac{k_{PYK,i,ATP}}{[\mathrm{ATP}] + k_{PYK,i,ATP}}\right) \cdot \left(\left(\frac{\boldsymbol{k_{PYK,i,PYR}}}{[\mathbf{PYR}] + \boldsymbol{k_{PYK,i,PYR,}}}\right)^{\boldsymbol{n_{PYK,i,PYR}}}\right) \cdot \left(\frac{\frac{[\mathrm{PEP}]}{k_{PYK,PEP}}^{n_{PYK,PEP}} \cdot \frac{[\mathrm{ADP}]}{k_{PYK,ADP}} \cdot \left(1 - \frac{\frac{[\mathrm{PYR}] \cdot [\mathrm{ATP}]}{[\mathrm{PEP}]^{n_{PYK,PEP}}\,[\mathrm{ADP}]}}{k_{PYK,EQ}}\right)}{\left(1 + \frac{[\mathrm{PEP}]}{k_{PYK,PEP}}\right)^{n_{PYK,PEP}} \cdot \left(1 + \frac{[\mathrm{ADP}]}{k_{PYK,ADP}}\right) + \left(1 + \frac{[\mathrm{PYR}]}{k_{PYK,PYR}}\right) \cdot \left(1 + \frac{[\mathrm{ATP}]}{k_{PYK,ATP}}\right) - 1}\right)$$

Parameter	Value	Unit	Reference
$\boldsymbol{v_{max,PYK}}$	**14.64**	[µmol/gDW/s]	**
$k_{PYK,EQ}$	24000	[-]	(Noor *et al*, 2013)
$k_{PYK,PEP}$	0.177	[µmol/gDW]	(Peskov *et al*, 2012)
$n_{PYK,PEP}$	1.05	[-]	(Speranza *et al*, 1990)
$k_{PYK,ADP}$	0.0027	[µmol/gDW]	**
$k_{PYK,PYR}$	0.3154	[µmol/gDW]	**
$k_{PYK,ATP}$	0.5357	[µmol/gDW]	**
$k_{PYK,a,FBP}$	5.28	[µmol/gDW]	**
$k_{PYK,i,ATP}$	22.5	[µmol/gDW]	(Chassagnole *et al*, 2002)
$n_{PYK,a,FBP}$	1.3	[-]	**
$\boldsymbol{k_{PYK,i,PYR}}$	**6.4**	[µmol/gDW]	**
$\boldsymbol{n_{PYK,i,PYR}}$	**2.8**	[-]	**

PFK reaction: parameter modification

$$v_{PFK} = v_{max,PFK} \cdot \left(\frac{[\mathrm{ADP}]}{k_{PFK,a,ADP} + [\mathrm{ADP}]}\right) \cdot \left(\frac{k_{PFK,i,PEP}}{k_{PFK,i,PEP} + [\mathrm{PEP}]}\right)^{n_{PFK,i,PEP}} \cdot \left(\frac{\frac{[\mathrm{ATP}]}{k_{PFK,ATP}} \cdot \left(\frac{[\mathrm{F6P}]}{k_{PFK,F6P}}\right)^{n_{PFK,F6P}} \cdot \left(1 - \frac{\frac{[\mathrm{ADP}]\,[\mathrm{FBP}]}{[\mathrm{ATP}]\,[\mathrm{F6P}]^{n_{PFK,F6P}}}}{k_{PFK,EQ}}\right)}{\left(1 + \frac{[\mathrm{ATP}]}{k_{PFK,ATP}}\right) \cdot \left(1 + \frac{[\mathrm{F6P}]}{k_{PFK,F6P}}\right)^{n_{PFK,F6P}} + \left(1 + \frac{[\mathrm{FBP}]}{k_{PFK,FBP}}\right) \cdot \left(1 + \frac{[\mathrm{ADP}]}{k_{PFK,ADP}}\right) - 1}\right)$$

Parameter	Value	Unit	Reference
$\boldsymbol{v_{max,PFK}}$	**18.74**	[µmol/gDW/s]	**
$k_{PFK,EQ}$	1300	[-]	(Noor *et al*, 2013)

$k_{PFK,ATP}$	1.137	[µmol/gDW]	**
$k_{PFK,F6P}$	0.0531	[µmol/gDW]	(Berger & Evans, 1991)
$k_{PFK,FBP}$	5	[µmol/gDW]	**
$k_{PFK,ADP}$	4.28	[µmol/gDW]	**
$k_{PFK,a,ADP}$	0.2886	[µmol/gDW]	**
$k_{PFK,i,PEP}$	3.45	[µmol/gDW]	(Ogawa *et al*, 2007)
$n_{PFK,i,PEP}$	2.61	[-]	**
$n_{PFK,F6P}$	1.9	[-]	(Ishii *et al*, 2007)

ATPase reaction: parameter modification

$$v_{ATPase} = v_{max,ATPase} \cdot \frac{[\mathrm{ATP}]^{\ n_{ATPase}}}{k_{ATPase,ATP}^{n_{ATPase}} + [\mathrm{ATP}]^{\ n_{ATPase}}}$$

Parameter	Value	Unit	Reference
$v_{max,ATPase}$	0 – 25 (variable)	[µmol/gDW/s]	**
$\boldsymbol{k_{ATPase,ATP}}$	**0.74**	[µmol/gDW]	**
$\boldsymbol{n_{ATPase}}$	**3.8**	[-]	**

Growth reaction: parameter modification

$$\mu = \mu_{max} \cdot \frac{[\mathrm{F6P}]}{k_{g,F6P} + [\mathrm{F6P}]} \cdot \frac{[\mathrm{GAP}]}{k_{g,GAP} + [\mathrm{GAP}]} \cdot \frac{[\mathrm{PEP}]}{k_{g,PEP} + [\mathrm{PEP}]} \cdot \frac{[\mathrm{PYR}]}{k_{g,PYR} + [\mathrm{PYR}]} \cdot \frac{[\mathrm{ATP}]^{n_{g,ATP}}}{k_{g,ATP}^{n_{g,ATP}} + [\mathrm{ATP}]^{n_{g,ATP}}}$$
$$\cdot \frac{[\mathrm{AcCoA}]}{k_{g,AcCoA} + [\mathrm{AcCoA}]} \cdot \frac{[\mathrm{AKG}]}{k_{g,AKG} + [\mathrm{AKG}]} \cdot \frac{[\mathrm{OAA}]}{k_{g,OAA} + [\mathrm{OAA}]} \cdot \frac{[\mathrm{NADH}]}{k_{g,NADH} + [\mathrm{NADH}]}$$
$$\cdot \frac{[\mathrm{G6P}]}{k_{g,G6P} + [\mathrm{G6P}]} \cdot \frac{[\mathrm{3PG}]}{k_{g,3PG} + [\mathrm{3PG}]} \cdot \frac{[\mathrm{FOR}]}{k_{g,FOR} + [\mathrm{FOR}]}$$

Parameter	Value	Unit	Reference
$\boldsymbol{\mu_{max}}$	**3.323e-04**	**[1/s]**	**
$k_{g,F6P}$	0.015	[µmol/gDW]	**
$k_{g,GAP}$	0.0021	[µmol/gDW]	**
$k_{g,PEP}$	0.0075	[µmol/gDW]	**
$k_{g,PYR}$	0.007	[µmol/gDW]	**
$\boldsymbol{k_{g,ATP}}$	**2.814**	**[µmol/gDW]**	**
$k_{g,AcCoA}$	0.0043	[µmol/gDW]	**
$k_{g,AKG}$	0.00143	[µmol/gDW]	**
$k_{g,OAA}$	0.009	[µmol/gDW]	**
$k_{g,NADH}$	7.2e-04	[µmol/gDW]	**
$k_{g,3PG}$	0.0018	[µmol/gDW]	**
$k_{g,G6P}$	0.075	[µmol/gDW]	**
$k_{g,FOR}$	0.0018	[µmol/gDW]	**
$n_{g,ATP}$	1.7	[-]	**

2.2 Simulations and reference points

As for kinetic model version 1, we chose (reference) $v_{max,ATPase}$ values for the different strains such that the simulated and the measured (steady-state) glucose uptake rate in the respective ATPase strain was identical. The reference points for model version 2 (cf. Figure 5 in main manuscript) changed slightly to: (1) WT strain: $v_{max,ATPase}$ = 0 mmol/(gDW h) (this represents the non-growth associated ATP maintenance demand without ATPase expression); (2) LC ATPase strain: $v_{max,ATPase}$ = 9.79 mmol/(gDW h); (3) MC ATPase strain: $v_{max,ATPase}$ = 37.72 mmol/(gDW h); and (4) HC ATPase strain: $v_{max,ATPase}$ = 68.68 mmol/(gDW h).

2.3 Metabolic control analysis with model version 2

Metabolic control analysis (MCA) was performed with the model version 2 for the HC ATPase strain to identify reaction steps having the highest control for the glycolytic flux (represented by glucose uptake via the PTS reaction). We determined the scaled flux control coefficients (FCCs) (Sauro, 2019) for the PTS reaction using the FCC calculation function of Copasi (Figure S2, left). The values indicate roughly the % (steady-state) flux change when increasing the v_{max} (i.e., the enzyme abundance) by 1%.

Since the FCCs are valid only for small (infinitesimal) changes, we also checked the fractional flux change of the steady state glycolytic flux when doubling the v_{max} (or the enzyme abundance) for each reaction (each separately). The results are shown in Figure S2 (right).

Figure S2: Left: (Ordered) scaled flux control coefficient for glucose uptake rate (PTS flux) determined for the HC ATPase strain (corresponding to a chosen value of $\boldsymbol{v_{max,ATPase}}$ = 68.68 [µmol/gDW/s] in the model version 2). The rows indicate the calculated FCCs for the respective reactions. **Right:** Percentage change of (steady-state) glucose uptake (PTS) flux when doubling the $\boldsymbol{v_{max}}$ (or enzyme abundance) of the respective reactions in the model (reactions ordered with respect to percentage change). No result is reported for doubling $\boldsymbol{v_{max}}$ of NGAM since this results in an unstable system.

Flux Control Coefficients	
	PTS
ATPase	-0.85
NGAM	-0.62
Growth	-0.29
FBA	-0.22
PTACK	-0.08
ADH	-0.07
ALDH	-0.006
CSICD	-0.0002
ENO	0.0003
ADK	0.001
PGK	0.003
PTS	0.004
PGI	0.006
PCK	0.01
MDH	0.01
FRD	0.02
GDH	0.04
PPC	0.32
PYK	0.42
LDH	0.54
PFK	0.85
PFL	0.92

Flux % change with 100% enzyme abundance increase	
	PTS
NGAM	
ATPase	-18.38
FBA	-17.64
Growth	-10.76
ADH	-4.54
PTACK	-4.32
ALDH	-0.40
CSICD	-0.14
ENO	-0.10
ADK	-0.08
PGK	0.03
PTS	0.06
PGI	0.18
MDH	0.77
PCK	0.88
FRD	0.97
GDH	3.08
PYK	29.01
PPC	30.05
PFK	34.51
LDH	36.10
PFL	163.42

3. Model calibrations and parameter estimations

For setting up the model, parameter values were extensively researched in literature and databases (see references given for the parameters in sections 1 and 2). In total, the model version 1 comprises 175 parameters (179 in model version 2), of which 97 (101 in model version 2) were considered unknown and therefore estimated. Unknown parameter values in model version 1 were initially defined through educated guesses (for example, unknown Michaelis-Menten constants were set to typical metabolite concentrations and $\boldsymbol{v_{max}}$ values to known maximal fluxes in the central metabolism). A first model calibration was then conducted manually to obtain metabolic fluxes and metabolite concentrations that roughly agree with the available measurements for all four strains (regarding measurements see Figure 5 in the main document). Afterwards, for model fine-tuning, parameters were fitted to the experimental data using available parameter estimation methods in *Copasi*. Due to the complex nature of the network, stochastic parameter estimation algorithms were preferred. Specifically, the *Genetic Algorithm* and *Evolutionary Programming* method implemented in *Copasi* were used. Due to the large number of parameters in the network and the relative scarcity of experimental data, only a smaller subset (36) of the 97 unknown parameters in model version 1 was selected for parameter estimation. For this purpose, parameters were ranked in a preprocessing step by performing a sensitivity analysis with respect to growth rate and glucose uptake rate. The most sensitive parameters were used for estimation. Reasonable lower and upper bounds were defined for each estimated parameter. To first fit model version 1, the measured growth rate, the substrate uptake rate and the product exchange fluxes of the wild type and the different ATPase strains (given in Table 1) as well as the corresponding metabolomics data were used in the fitting procedure. As described in the main text and in section 2 above, for constructing model version 2, two inhibition terms were introduced in the PFL and PYK reactions (see section 2). The so obtained model was then again fitted to the experimental data. This time, only the parameters of the newly introduced terms as well as μ_{max} and ATP-related parameters of the growth rate as well as the $\boldsymbol{v_{max}}$of the PFK were fitted in the parameter estimation procedure.

<u>Comment</u>: With the relatively large model size and considerable number of parameters, many parameters will not be uniquely identifiable in the presented kinetic model, despite the available experimental datasets used for parameter estimation. However, the main goals of the presented model in our study are (i) to show that we can build, in a bottom-up manner, a kinetic model that includes basic mechanisms and biological knowledge of the central metabolism of *E. coli* and that this model can reproduce the experimental findings; (ii) to use this model to gain insights regarding the biphasic (steady-state) response curve of the glucose uptake rate, and (iii) to make meaningful predictions that can be verified with experiments (and thereby to strengthen the confidence in the model). In fact, even with non-identifiable parameters, our model shows that our argumentation with the kinetics of the PFK is one possible mechanistic interpretation regarding the biphasic response curve of glucose uptake and the model's predictive capabilities could be clearly demonstrated (see MCA analysis of model version 2 above and the experimental validation in the main document). However, as is true for all models, this cannot prove correctness of the model (or of our hypothesis) since the existence of other plausible models that can also explain the observations can never be excluded. Generally, a model is useful if it can exclude certain behaviors. Here, the fact that the first version of the kinetic model (also after several rounds of parameter fitting) could not reflect the measured high concentrations of glycolytic metabolites already demonstrates that the kinetic model cannot be calibrated to arbitrary data and observations.

Furthermore, predictions of the model should, at least qualitatively, be robust against parameter variations. This issue is addressed in the following section.

4. Using Monte-Carlo sampling of kinetic parameters to assess the robustness of model predictions

The kinetic model of *E coli*'s central metabolism constructed in the previous section is relatively large and comprises many unknown parameters. Even with a larger set of experimental data, parameters remain often non-identifiable in such models. These uncertainties in parameter values may translate to uncertainties in model predictions. We therefore used Monte-Carlo (MC) sampling of kinetic parameters to assess the robustness of model predictions with respect to the choice of kinetic parameters. To this end, we employed the approach previously used in (Murabito *et al*, 2014). This approach avoids a shortcoming of conventional MC analysis: A straightforward sampling of kinetic parameters is either restricted to parameters in the close vicinity of a reference set, or, if larger variations are considered, the sampled parameter sets give rise to different new metabolic steady states - but these newly identified *in-silico* steady states are typically neither physiological, nor are they suitable to characterize the specific metabolic reference state (wild type and HC ATPase) considered in our analysis. Following (Murabito *et al*, 2014), we therefore sampled all Michaelis-Menten parameters such that the metabolic reference state, to which the original parameters were fitted, was preserved for each set of sampled parameters. For each model with its randomly sampled parameters it is possible to determine specific systems properties, doing that for all parameter sets a probabilistic distribution of this property is obtained. Here, we focused on the flux control coefficients (FCCs), which are global control properties of a kinetic model and were also used for testing the effects of overexpressing certain enzymes in the HC ATPase strain (see main manuscript).

For a complete description of the method, we refer to (Murabito *et al*, 2014). Briefly, we sampled all Michaelis-Menten parameters of the kinetic rate laws (but not the equilibrium constants) in a range of two orders of magnitude (one order of magnitude above and one below) of the respective original parameter value in the kinetic model (version 2) described above. According to Murabito et al, given a particular parameter sample, together with the known steady-state fluxes and metabolite concentrations of the reference state, it is possible to uniquely determine the $\boldsymbol{v_{max}}$ values of the rate laws such that the so obtained model reproduces the reference state. Only parameter sets giving rise to a stable model were retained. Stability is ensured when all eigenvalues of the associated Jacobian matrix have a negative real part, which is directly checked with this method (Murabito *et al*, 2014). For each sampled parameter set that leads to a stable model, we then calculated all FCCs (of all pairs of controlling enzyme vs. affected fluxes) in the model.

We considered two different scenarios (reference states) under anaerobic growth: (a) the wild type strain and (b) the HC ATPase strain. The steady-state fluxes and metabolite concentrations of the reference state were directly taken from model version 2, which has a good fit with measured fluxes and concentrations. For both reference states, we sampled 10^4 parameter sets and computed then the respective FCCs for the stable models. The script files used for the MC study and further information can be found on https://github.com/klamt-lab/Models_E.coli_High_ATP_Demand/tree/main/Kinetic%20Model/Monte%20Carlo%20Parameter%20Sampling

Figure S3 shows the probabilistic distribution of all FCCs for the wild-type metabolic steady state. Since the parameters are chosen randomly, we do not interpret individual values of the FCCs. Rather, the resulting distributions are interpreted with respect to their width, indicating the variability of the FCCs with respect to changes in parameters, as well as with respect to their sign as a qualitative property. We consider a FCC to be sign-dominant when more than 80% of sampled instances share the same sign. The latter indicates that, while the precise value of a FCC may depend on parameters, the qualitative impact upon a change in enzyme levels can be robustly predicted. Generally, we found predominantly narrow distributions for the FCCs, indicating a low sensitivity of these FCCs against parameter variations. The control properties of the ensemble of sampled models are in good agreement with the FCCs obtained from the fitted model (shown as red lines in Figure S3). We also recovered the well-known fact that reactions close to equilibrium

typically exert low control over a pathway and that control is shared between several enzymes. The FCCs of few enzymes show broader distributions, but those are typically sign-dominant. One example of an enzyme with broader FCC distributions is the PFK, a known regulator of glycolytic flux. We also noticed that the FCC distribution of the NGAM reaction on PTS was narrow with predominantly positive sign (>95% of sampled instances), confirming that the increase of glucose uptake in the wild type as response to higher ATP demand is a robust feature of the model.

The respective FCC distributions with the HC ATPase strain as reference state are shown in Figure S4. Although the majority of distributions are still narrow, we observe that the fraction of enzymes with a broader distribution of FCC increased, indicating a higher sensitivity to parameter variations, which can also be interpreted as reduced robustness of the HC ATPase metabolic state. In particular, in addition to the PFK, the FCC for the enzymes PYK, PFL and lactate dehydrogenase (LDH) now exhibit a broader (but still positive sign-dominant) distribution, confirming that the FCCs obtained with the kinetic model (section 2.3) and used for experimental validation of model predictions are robust properties of the system. It can also be noticed that the FCC of the NGAM (and ATPase) reaction on PTS and glycolytic flux are here predominantly negative. Together with the predominantly positive FCC of NGAM on PTS and glycolytic flux in the wild type, this confirms the bi-phasic response of the glycolytic flux to increased ATP demand as a robust feature of the model.

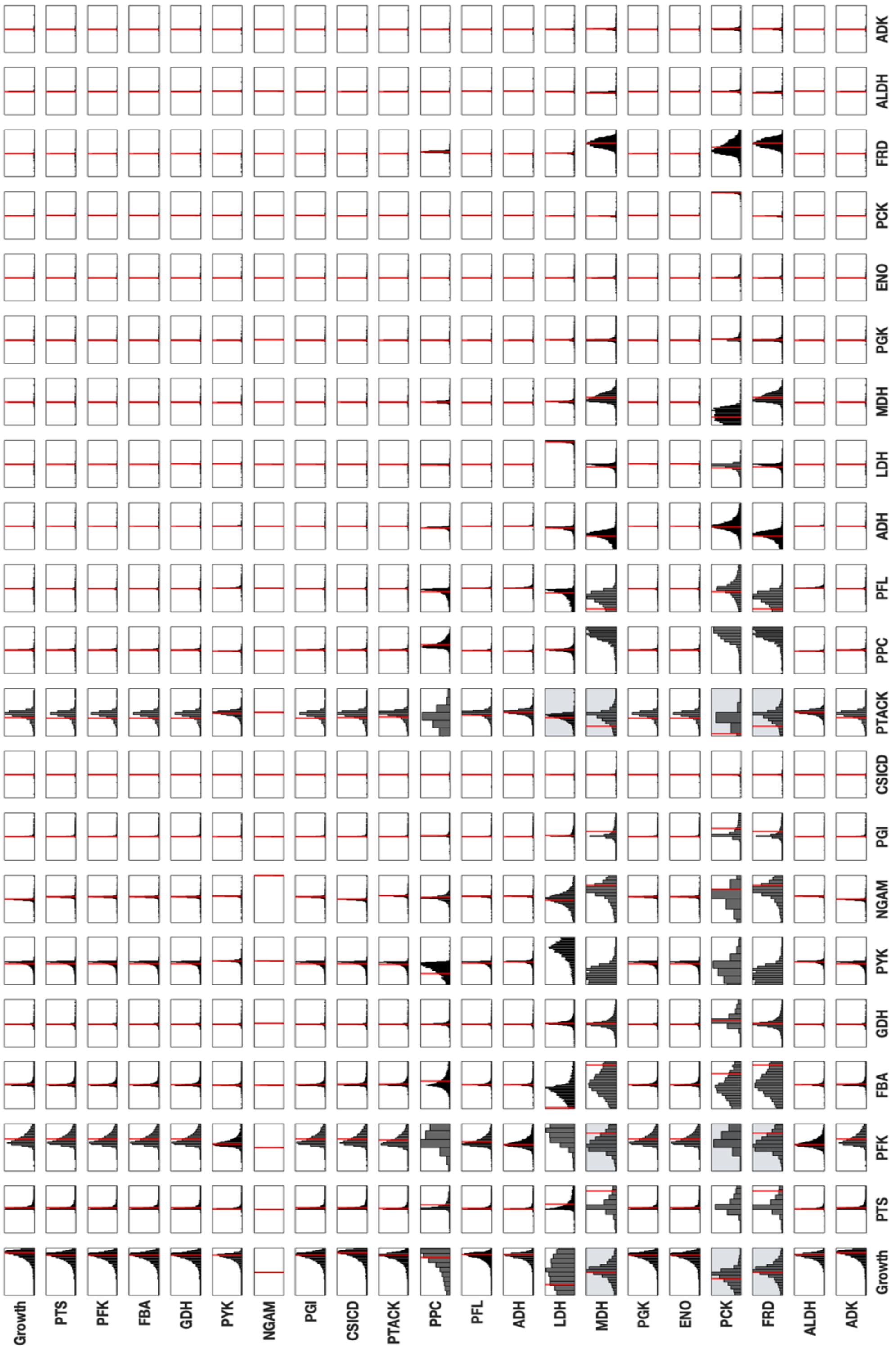

Figure S3: Probability distribution of scaled flux control coefficients obtained by sampling kinetic parameters of model version 2 for wild-type conditions ($v_{max,ATPase} = 0$). Columns represent the controlling enzyme (cause) and rows the controlled flux (effect). In each plot, the abscissa corresponds to the FCC with an interval of [-1,1], except for the plots with dark background where the interval is [-10,10]. The red lines indicate the FCCs calculated with the (fitted) parameter set of model version 2 for wild-type conditions. Since the ATPase flux is zero in the wild type, the corresponding reaction was not included in the sampling procedure.

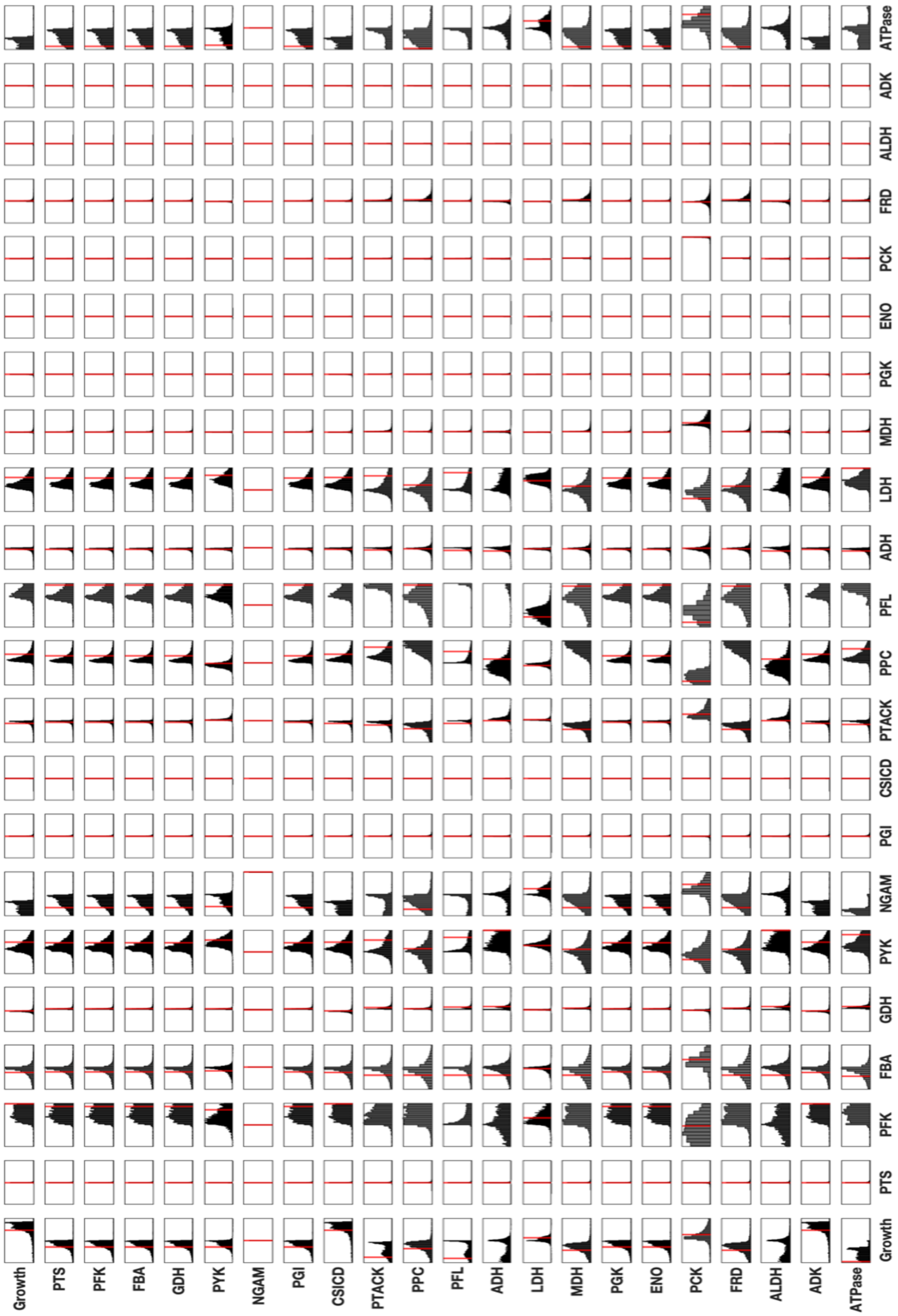

Figure S4: Probability distribution of scaled flux control coefficients obtained by sampling kinetic parameters of model version 2 for HC ATPase conditions (high $v_{max,ATPase}$). Columns represent the controlling enzyme (cause) and rows the controlled flux (effect). In each plot, the abscissa corresponds to the FCC with an interval of [-1,1]. The red lines indicate the FCCs calculated with the (fitted) parameter set of model version 2 for HC ATPase conditions (for the FCC governing the PTS flux in the HC ATPase strain cf. also with Figure S2).

Supplementary Tables

Table S1. Strains, plasmids and primers used in this study

Strain	Relevant characteristics	Reference
E. coli NEB 5-alpha	Competent cells for cloning and plasmid propagation	New England Biolabs, #C2987U
E. coli MG1655	*E. coli* wild type strain	(Blattner *et al*, 1997)
LC control strain	*E. coli* MG1655 transformed with plasmid pSB60.1	This study
MC control strain	*E. coli* MG1655 transformed with plasmid pSB68.1	This study
HC control strain	*E. coli* MG1655 transformed with plasmid pSB64.1	This study
LC ATPase strain	*E. coli* MG1655 transformed with plasmid pSB58.6	This study
MC ATPase strain	*E. coli* MG1655 transformed with plasmid pSB66.1	This study
HC ATPase strain	*E. coli* MG1655 transformed with plasmid pSB62.6	This study
HC ATPase strain +*pfkA*	*E. coli* MG1655 transformed with plasmid pSB84.3	This study
HC ATPase Strain +*pflB*	*E. coli* MG1655 transformed with plasmid pSB85.3	This study
HC ATPase Strain +*pfkA* +*pflB*	*E. coli* MG1655 transformed with plasmid pSB86.4	This study
HC ATPase Strain +*pgk*	*E. coli* MG1655 transformed with plasmid pSB88.11	This study
**Plasmid**	**Relevant characteristics**	**Reference**
pCP41::*atpAGD*	*atpAGD* under control of constitutive CP41-*lacLM* promoter, Erm^R	(Koebmann *et al*, 2002)
pSB-T1g	*gfpmut3* under control of IPTG inducible $lacI^q/P_{trc}$ promoter system, RK2 replicon (low copy), Kan^R	(Balzer *et al*, 2013)
pSB-T2g	*gfpmut3* under control of IPTG inducible $lacI^q/P_{trc}$ promoter system, pMB1 replicon (high copy), Kan^R	(Balzer *et al*, 2013)
pZA31-luc	*luc* under control of doxycyclin inducible $P_{LtetO\text{-}1}$ promoter system, p15A replicon (medium copy), Cm^R	(Lutz & Bujard, 1997)
pSB58.6	*atpAGD* under control of IPTG inducible $lacI^q/P_{trc}$ promoter system, RK2 replicon (low copy), Kan^R	This study
pSB60.1	pSB58.6 without *atpAGD* (empty control plasmid), RK2 replicon (low copy), Kan^R	This study
pSB62.6	*atpAGD* under control of IPTG inducible $lacI^q/P_{trc}$ promoter system, pMB1 replicon (high copy), Kan^R	This study
pSB64.1	pSB62.6 without *atpAGD* (empty control plasmid), pMB1 replicon (high copy), Kan^R	This study
pSB66.1	*atpAGD* under control of IPTG inducible $lacI^q/P_{trc}$ promoter system, p15A replicon (medium copy), Kan^R	This study
pSB68.1	pSB66.1 without *atpAGD* (empty control plasmid), p15A replicon (medium copy), Kan^R	This study

pSB84.3	*atpAGD+pfkA* under control of IPTG inducible *lacI*^{q}/P_{trc} promoter system, pMB1 replicon (high copy), Kan^R	This study
pSB85.3	*atpAGD+pflB* under control of IPTG inducible *lacI*^{q}/P_{trc} promoter system, pMB1 replicon (high copy), Kan^R	This study
pSB86.4	*atpAGD+pfkA+pflB* under control of IPTG inducible *lacI*^{q}/P_{trc} promoter system, pMB1 replicon (high copy), Kan^R	This study
pSB88.11	*atpAGD+pgk* under control of IPTG inducible *lacI*^{q}/P_{trc} promoter system, pMB1 replicon (high copy), Kan^R	This study

**Primer**	**Sequence (5' → 3')**
atpAGD_mono_fw	CATGAACATATGCAACTGAATTCCACCGAAATC
atpAGD_mono_rv	CTAGAGGATCCTTAAAGTTTTTTGGCTTTTTCC
p15A_SpeI_fw	GATCACTAGTAACAACTTATATCGTATG
p15A_AscI_rv	CTGAGGCGCGCCGGATATATTCCGCTTCCTCGCTC
pSB73.4_Gibson_fw	GGATCCTCTAGCTAGAGTCAGC
atpD_rv	TTAAAGTTTTTTGGCTTTTTCC
pfkA_rbs_fw	TGTGGAAAAAGCCAAAAAACTTTAA*CGCCTTAATCGGAGGGTGAT* ATGATTAAGAAAATCGGTGTGTTG*
pfkA_rbs_rv	CTGACTCTAGCTAGAGGATCCTTAATACAGTTTTTTCGCGCAGTCC
pflB_rbs_fw	TGTGGAAAAAGCCAAAAAACTTTAA*CGCCTTAATCGGAGGGTGAT* ATGTCCGAGCTTAATGAAAAGTTAG*
pflB_rbs_rv	CTGACTCTAGCTAGAGGATCCTTACATAGATTGAGTGAAGGTACG
pgk_rbs_fw	TGTGGAAAAAGCCAAAAAACTTTAA*CGCCTTAATCGGAGGGTGAT* ATGTCTGTAATTAAGATGACCGATC*
pgk_rbs_rv	CTGACTCTAGCTAGAGGATCCTTACTTCTTAGCGCGCTCTTCGAGC
pfkA_rv	TTAATACAGTTTTTTCGCGCAGTCC
pflB_rbs2_fw	GGACTGCGCGAAAAAACTGTATTAA*CGCCTTAATCGGAGGGTGAT* ATGTCCGAGCTTAATGAAAAGTTAG*

*) The inserted ribosomal binding sites are marked in italics

Table S2. Specific growth, glucose uptake, and acetate synthesis rates and acetate yields of the tested strains under aerobic growth. Regarding the calculation of the ATPM and the ATPase rates see Methods. The averages ± standard deviations of *n* = 3 biologically independent samples are shown.

	**WT**	**WT + IPTG**	**LC control**	**LC ATPase**	**MC control**	**MC ATPase**	**HC control**	**HC ATPase**
**μ [h^{-1}]**	0.652 ± 0.002	0.668 ± 0.002	0.703 ± 0.004	0.580 ± 0.003	0.673 ± 0.003	0.474 ± 0.008	0.708 ± 0.002	0.040 ± 0.001
**r_{Glc} [mmol/gDW/h]**	7.62 ± 0.43	8.11 ± 1.30	9.39 ± 0.07	10.00 ± 0.19	8.09 ± 0.35	12.07 ± 0.23	8.77 ± 0.89	5.34 ± 0.17
**r_{Ace} [mmol/gDW/h]**	3.98 ± 0.21	4.24 ± 0.51	4.09 ± 0.65	7.02 ± 0.68	3.39 ± 0.32	12.12 ± 0.32	4.64 ± 0.69	7.87 ± 0.83
**r_{ATPM} (calculated) [mmol/gDW/h]**	4.11 ± 5.80	18.54 ± 16.03	29.14 ± 3.81	54.30 ± 2.28	10.71 ± 8.42	96.60 ± 6.41	18.49 ± 14.06	66.91 ± 4.53
**r_{ATPase} (calculated) [mmol/gDW/h]**				25.16		85.89		48.42
**Y_{BM} [gDW/g]**	0.485 ± 0.001	0.414 ± 0.011	0.424 ± 0.002	0.311 ± 0.003	0.479 ± 0.004	0.186 ± 0.000	0.408 ± 0.003	0.032 ± 0.004
**Y_{Ace} [mol/mol]**	0.544 ± 0.023	0.532 ± 0.117	0.436 ± 0.070	0.680 ± 0.063	0.442 ± 0.061	0.996 ± 0.043	0.512 ± 0.108	1.393 ± 0.081

Table S3. Specific growth, glucose uptake, and acetate synthesis rates and acetate yields of the tested strains under aerobic conditions with growth arrest. Regarding the calculation of the ATPM and the ATPase rates see Methods. The averages ± standard deviations of n = 3 biologically independent samples are shown.

	**WT**	**WT + IPTG**	**LC control**	**LC ATPase**	**MC control**	**MC ATPase**	**HC control**	**HC ATPase**
**μ [h^{-1}]**	no exp. growth	no exp. growth	no exp. growth	no exp. growth	no exp. growth	~0	no exp. growth	~0
**r_{Glc} [mmol/gDW/h]**	1.05 ± 0.06	0.89 ± 0.15	1.08 ± 0.05	4.90 ± 0.05	0.91 ± 0.17	10.16 ± 0.30	1.31 ± 0.14	7.00 ± 0.28
**r_{Ace} [mmol/gDW/h]**	0.72 ± 0.04	0.76 ± 0.03	0.59 ± 0.03	4.01 ± 0.18	0.80 ± 0.03	7.21 ± 0.20	0.91 ± 0.09	5.28 ± 0.35
**r_{ATPM} (calculated) [mmol/gDW/h]**	20.17 ± 1.63	16.28 ± 3.27	21.66 ± 1.16	90.13 ± 1.98	16.40 ± 4.06	193.71 ± 7.94	25.00 ± 3.62	131.36 ± 8.39
**r_{ATPase} (calculated) [mmol/gDW/h]**				68.47		177.31		106.36
**Y_{BM} [gDW/g]**	0.194 ± 0.004	0.212 ± 0.012	0.197 ± 0.010	0.031 ± 0.001	0.168 ± 0.010	~0	0.096 ± 0.006	~0
**Y_{Ace} [mol/mol]**	0.629 ± 0.026	0.695 ± 0.041	0.474 ± 0.055	0.840 ± 0.014	0.774 ± 0.036	0.827 ± 0.024	0.649 ± 0.012	0.718 ± 0.034

Table S4. Specific growth, glucose uptake, fumarate uptake, and product synthesis rates of the HC control and HC ATPase strains under anaerobic growth with (+Fum) and without (-Fum) fumarate addition. Regarding the calculation of the ATPM and the ATPase rates see Methods. The averages ± standard deviations of *n* = 3 (-Fum) and *n* = 2 (+Fum) biologically independent samples are shown. *P* values for a two-sample t test are given with respect to the cultivations without fumarate addition of the respective strains. *P* > 0.05 was considered as not significant (n.s.). See also Table S2 and Fig. EV5.

	**HC control -Fum (from Table S2)**	**HC control +Fum**	**HC ATPase -Fum (from Table S2)**	**HC ATPase +Fum**
**μ [h^{-1}]**	0.431 ± 0.004	0.404 ± 0.002	0.063 ± 0.009	0.019 ± 0.001
**r_{Glc} [mmol/gDW/h]**	12.93 ± 1.11	11.75 ± 0.45 (*P* = 0.350, n.s.)	5.13 ± 0.51	10.80 ± 0.02 (*P* = 0.001)
**r_{Fum} [mmol/gDW/h]**	-	7.02 ± 0.36	-	9.00 ± 0.15
**r_{Eth} [mmol/gDW/h]**	9.84 ± 0.52	7.87 ± 0.08	2.29 ± 0.51	1.30 ± 0.11
**r_{Ace} [mmol/gDW/h]**	9.89 ± 0.24	10.46 ± 0.79	3.28 ± 0.39	5.81 ± 0.00
**r_{For} [mmol/gDW/h]**	20.46 ± 0.66	20.07 ± 1.25	4.69 ± 0.96	4.51 ± 0.25
**r_{Lac} [mmol/gDW/h]**	0.83 ± 0.09	1.06 ± 0.22	2.95 ± 0.13	14. 58 ± 0.13
**r_{Suc} [mmol/gDW/h]**	1.65 ± 0.09	5.33 ± 0.43	0.45 ± 0.17	9.15 ± 0.16
**r_{Pyr} [mmol/gDW/h]**	-	0.68 ± 0.05	-	1.26 ± 0.27
**r_{ATPM} (calculated) [mmol/gDW/h]**	2.67 ± 1.05	9.97 ± 0.98	6.99 ± 0.73	29.42 ± 0.46
**r_{ATPase} (calculated) [mmol/gDW/h]**			4.32	19.45

References

Anderson DH, Duckworth HW (1988) *In vitro* mutagenesis of *Escherichia coli* citrate synthase to clarify the locations of ligand binding sites. *J Biol Chem* 263: 2163-2169

Babul J, Clifton D, Kretschmer M, Fraenkel DG (1993) Glucose metabolism in *Escherichia coli* and the effect of increased amount of aldolase. *Biochemistry* 32: 4685-4692

Balzer S, Kucharova V, Megerle J, Lale R, Brautaset T, Valla S (2013) A comparative analysis of the properties of regulated promoter systems commonly used for recombinant gene expression in *Escherichia coli*. *Microb Cell Fact* 12: 26

Berger SA, Evans PR (1991) Steady-state fluorescence of *Escherichia coli* phosphofructokinase reveals a regulatory role for ATP. *Biochemistry* 30: 8477-8480

Bettenbrock K, Fischer S, Kremling A, Jahreis K, Sauter T, Gilles ED (2006) A quantitative approach to catabolite repression in *Escherichia coli*. *J Biol Chem* 281: 2578-2584

Blattner FR, Plunkett III G, Bloch CA, Perna NT, Burland V, Riley M, Collado-Vides J, Glasner JD, Rode CK, Mayhew GF *et al.* (1997) The Complete Genome Sequence of *Escherichia coli* K-12. *Science* 277: 1453

Campos-Bermudez VA, Bologna FP, Andreo CS, Drincovich MF (2010) Functional dissection of *Escherichia coli* phosphotransacetylase structural domains and analysis of key compounds involved in activity regulation. *FEBS J* 277: 1957-1966

Chassagnole C, Noisommit-Rizzi N, Schmid JW, Mauch K, Reuss M (2002) Dynamic modeling of the central carbon metabolism of *Escherichia coli*. *Biotechnol Bioeng* 79: 53-73

Cintolesi A, Clomburg JM, Rigou V, Zygourakis K, Gonzalez R (2012) Quantitative analysis of the fermentative metabolism of glycerol in Escherichia coli. *Biotechnol Bioeng* 109: 187-198

Danson MJ, Weitzman PD (1973) Functional groups in the activity and regulation of *Escherichia coli* citrate synthase. *Biochem J* 135: 513-524

Erdrich P, Steuer R, Klamt S (2015) An algorithm for the reduction of genome-scale metabolic network models to meaningful core models. *BMC Syst Biol* 9: 48

Fox DK, Roseman S (1986) Isolation and characterization of homogeneous acetate kinase from *Salmonella typhimurium* and *Escherichia coli*. *J Biol Chem* 261: 13487-13497

Hoefnagel MHN, Starrenburg MJC, Martens DE, Hugenholtz J, Kleerebezem M, Van S, II, Bongers R, Westerhoff HV, Snoep JL (2002) Metabolic engineering of lactic acid bacteria, the combined approach: kinetic modelling, metabolic control and experimental analysis. *Microbiology (Reading, Engl)* 148: 1003-1013

Hoops S, Sahle S, Gauges R, Lee C, Pahle J, Simus N, Singhal M, Xu L, Mendes P, Kummer U (2006) COPASI — a COmplex PAthway SImulator. *Bioinformatics* 22: 3067-3074

Ishii N, Suga Y, Hagiya A, Watanabe H, Mori H, Yoshino M, Tomita M (2007) Dynamic simulation of an in vitro multi-enzyme system. *FEBS Lett* 581: 413-420

Iwakura M, Hattori J, Arita Y, Tokushige M, Katsuki H (1979) Studies on regulatory functions of malic enzymes. VI. Purification and molecular properties of NADP-linked malic enzyme from *Escherichia coli* W. *J Biochem* 85: 1355-1365

Izui K, Sabe H, Katsuki H (1981) Increased synthesis of phosphoenolypyruvate carboxylase in a strain of *Escherichia coli* bearing a ColE1-ppc$^+$ hybrid plasmid. *FEBS Lett* 133: 311-315

Kai Y, Matsumura H, Inoue T, Terada K, Nagara Y, Yoshinaga T, Kihara A, Tsumura K, Izui K (1999) Three-dimensional structure of phosphoenolpyruvate carboxylase: a proposed mechanism for allosteric inhibition. *Proc Natl Acad Sci USA* 96: 823-828

Kim SY, Hwang KY, Kim SH, Sung HC, Han YS, Cho Y (1999) Structural basis for cold adaptation. Sequence, biochemical properties, and crystal structure of malate dehydrogenase from a psychrophile *Aquaspirillium arcticum*. *J Biol Chem* 274: 11761-11767

Klamt S, Saez-Rodriguez J, Gilles ED (2007) Structural and functional analysis of cellular networks with *CellNetAnalyzer*. *BMC Syst Biol* 1: 2

Knappe J, Blaschkowski HP, Gröbner P, Schmitt T (1974) Pyruvate formate-lyase of *Escherichia coli*: the acetyl-enzyme intermediate. *Eur J Biochem* 50: 253-263

Knappe J, Sawers G (1990) A radical-chemical route to acetyl-CoA: the anaerobically induced pyruvate formate-lyase system of Escherichia coli. *FEMS Microbiol Rev* 6: 383-398

Koebmann BJ, Westerhoff HV, Snoep JL, Nilsson D, Jensen PR (2002) The Glycolytic Flux in *Escherichia coli* Is Controlled by the Demand for ATP. *J Bacteriol* 184: 3909-3916

Kotte O, Zaugg JB, Heinemann M (2010) Bacterial adaptation through distributed sensing of metabolic fluxes. *Mol Syst Biol* 6: 355

Krebs A, Bridger WA (1980) The kinetic properties of phosphoenolpyruvate carboxykinase of *Escherichia coli*. *Can J Biochem* 58: 309-318

Kuntz GW, Krietsch WK (1982) Phosphoglycerate kinase from spinach, blue-green algae, and yeast. *Methods Enzymol* 90 Pt E: 110-114

Lambeir AM, Loiseau AM, Kuntz DA, Vellieux FM, Michels PA, Opperdoes FR (1991) The cytosolic and glycosomal glyceraldehyde-3-phosphate dehydrogenase from *Trypanosoma brucei*. Kinetic properties and comparison with homologous enzymes. *Eur J Biochem* 198: 429-435

Liebermeister W, Uhlendorf J, Klipp E (2010) Modular rate laws for enzymatic reactions: thermodynamics, elasticities and implementation. *Bioinformatics* 26: 1528-1534

Lutz R, Bujard H (1997) Independent and tight regulation of transcriptional units in *Escherichia coli* via the LacR/O, the TetR/O and AraC/I_1-I_2 regulatory elements. *Nucleic Acids Res* 25: 1203-1210

Maklashina E, Iverson TM, Sher Y, Kotlyar V, Andréll J, Mirza O, Hudson JM, Armstrong FA, Rothery RA, Weiner JH *et al.* (2006) Fumarate reductase and succinate oxidase activity of *Escherichia coli* complex II homologs are perturbed differently by mutation of the flavin binding domain. *J Biol Chem* 281: 11357-11365

Matsuoka Y, Kurata H (2017) Modeling and simulation of the redox regulation of the metabolism in *Escherichia coli* at different oxygen concentrations. *Biotechnol Biofuels* 10: 183

Millard P, Smallbone K, Mendes P (2017) Metabolic regulation is sufficient for global and robust coordination of glucose uptake, catabolism, energy production and growth in *Escherichia coli*. *PLOS Comput Biol* 13: e1005396

Murabito E, Verma M, Bekker M, Bellomo D, Westerhoff HV, Teusink B, Steuer R (2014) Monte-Carlo modeling of the central carbon metabolism of *Lactococcus lactis*: insights into metabolic regulation. *PLOS ONE* 9: e106453

Muslin EH, Li D, Stevens FJ, Donnelly M, Schiffer M, Anderson LE (1995) Engineering a domain-locking disulfide into a bacterial malate dehydrogenase produces a redox-sensitive enzyme. *Biophys J* 68: 2218-2223

Noor E, Haraldsdottir HS, Milo R, Fleming RM (2013) Consistent estimation of Gibbs energy using component contributions. *PLOS Comput Biol* 9: e1003098

Ogawa T, Mori H, Tomita M, Yoshino M (2007) Inhibitory effect of phosphoenolpyruvate on glycolytic enzymes in *Escherichia coli*. *Res Microbiol* 158: 159-163

Orth JD, Conrad TM, Na J, Lerman JA, Nam H, Feist AM, Palsson BO (2011) A comprehensive genome-scale reconstruction of *Escherichia coli* metabolism—2011. *Mol Syst Biol* 7: 535

Park JO, Rubin SA, Xu Y-F, Amador-Noguez D, Fan J, Shlomi T, Rabinowitz JD (2016) Metabolite concentrations, fluxes and free energies imply efficient enzyme usage. *Nat Chem Biol* 12: 482-489

Peskov K, Goryanin I, Demin O (2008) Kinetic Model of Phosphofructokinase-1 from *Escherichia coli*. *J Bioinform Comput Biol* 6: 843-867

Peskov K, Mogilevskaya E, Demin O (2012) Kinetic modelling of central carbon metabolism in *Escherichia coli*. *FEBS J* 279: 3374-3385

Robinson MS, Easom RA, Danson MJ, Weitzman PDJ (1983) Citrate synthase of *Escherichia coli*. *FEBS Lett* 154: 51-54

Rohwer JM, Meadow ND, Roseman S, Westerhoff HV, Postma PW (2000) Understanding glucose transport by the bacterial phosphoenolpyruvate:glycose phosphotransferase system on the basis of kinetic measurements *in vitro*. *J Biol Chem* 275: 34909-34921

Sauro MH (2019) *Systems Biology: An Introduction to Metabolic Control Analysis*. Ambrosius Publishing, USA

Shone CC, Fromm HJ (1981) Steady-state and pre-steady-state kinetics of coenzyme A linked aldehyde dehydrogenase from *Escherichia coli*. *Biochemistry* 20: 7494-7501

Smith TE, Balasubramanian KA, Beezley A (1980) *Escherichia coli* phosphoenolpyruvate carboxylase. Studies on the mechanism of synergistic activation by nucleotides. *J Biol Chem* 255: 1635-1642

Somani BL, Valentini G, Malcovati M (1977) Purification and molecular properties of the AMP-activated pyruvate kinase from *Escherichia coli*. *Biochim Biophys Acta* 482: 52-63

Speranza ML, Valentini G, Malcovati M (1990) Fructose-1,6-bisphosphate-activated pyruvate kinase from *Escherichia coli*. Nature of bonds involved in the allosteric mechanism. *Eur J Biochem* 191: 701-704

Spring TG, Wold F (1971) The purification and characterization of *Escherichia coli* enolase. *J Biol Chem* 246: 6797-6802

Tarmy EM, Kaplan NO (1968) Chemical characterization of D-lactate dehydrogenase from *Escherichia coli* B. *J Biol Chem* 243: 2579-2586

Vorobieva AA, Khan MS, Soumillion P (2014) *Escherichia coli* D-malate dehydrogenase, a generalist enzyme active in the leucine biosynthesis pathway. *J Biol Chem* 289: 29086-29096

Watabe K, Freese E (1979) Purification and properties of the manganese-dependent phosphoglycerate mutase of *Bacillus subtilis*. *J Bacteriol* 137: 773-778

Wohl RC, Markus G (1972) Phosphoenolpyruvate carboxylase of *Escherichia coli*. Purification and some properties. *J Biol Chem* 247: 5785-5792

Wratten CC, Cleland WW (1963) Product Inhibition Studies on Yeast and Liver Alcohol Dehydrogenases*. *Biochemistry* 2: 935-941

Wright SK, Zhao FJ, Rardin J, Milbrandt J, Helton M, Furumo NC (1995) Mechanistic studies on malate dehydrogenase from *Escherichia coli*. *Arch Biochem Biophys* 321: 289-296

Yano M, Izui K (1997) The replacement of Lys620 by serine desensitizes *Escherichia coli* phosphoenolpyruvate carboxylase to the effects of the feedback inhibitors L-aspartate and L-malate. *Eur J Biochem* 247: 74-81

Zhang X, Jantama K, Moore JC, Jarboe LR, Shanmugam KT, Ingram LO (2009) Metabolic evolution of energy-conserving pathways for succinate production in *Escherichia coli*. *Proc Natl Acad Sci USA* 106: 20180-20185
