# Supplementary material for: Deciphering the physiological response of Escherichia coli under high ATP demand
Source: Mol Syst Biol. 2021 Dec 20;17(12):e10504. doi: 10.15252/msb.202110504 (PMC8686765; doi:10.15252/msb.202110504)
Supplement: Supplementary file 2 — Expanded View Figures PDF [file MSB-17-e10504-s006.pdf]

Expanded View Figures

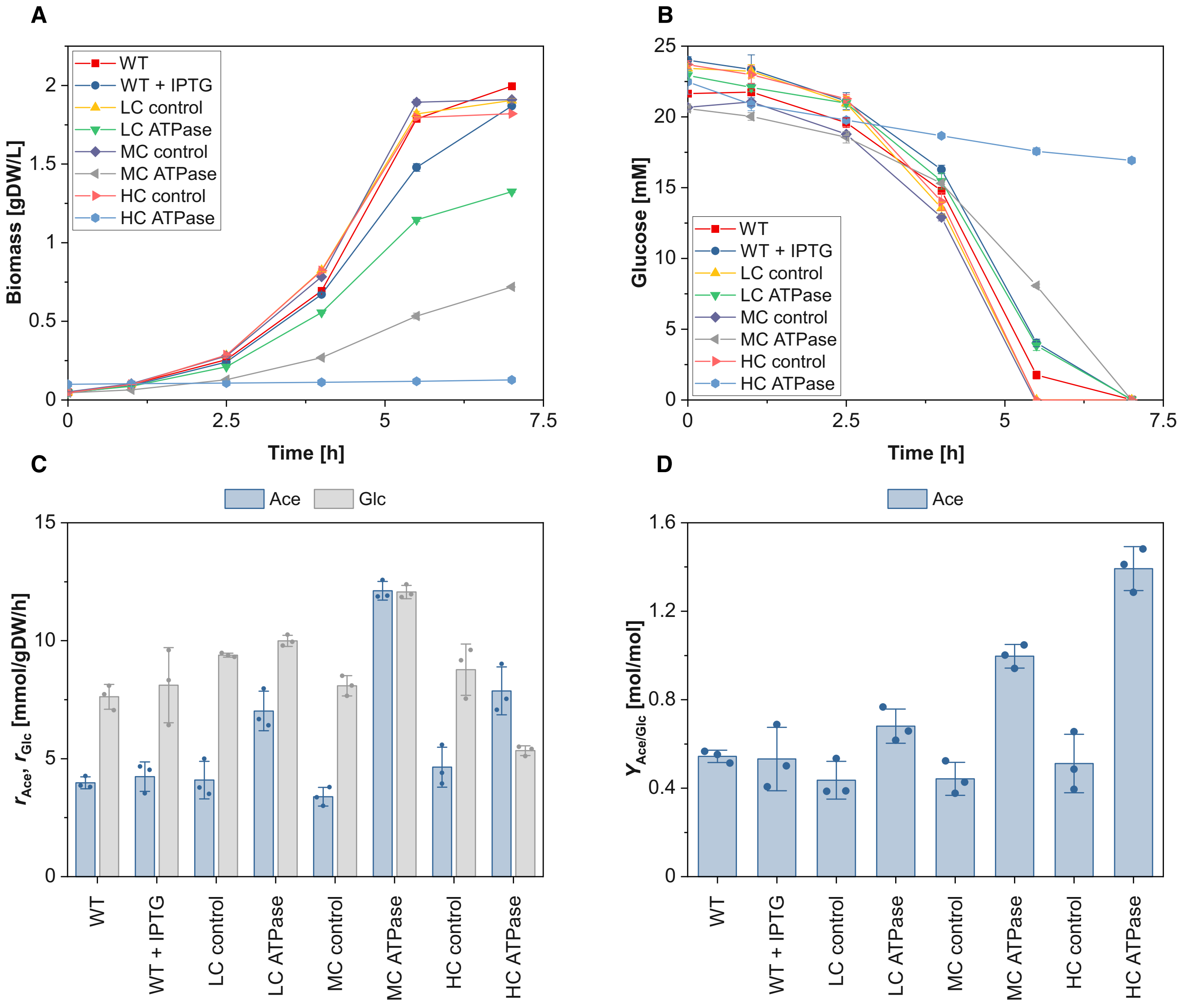

Figure EV1. Aerobic growth of the different ATPase strains.

A Time course of biomass concentration.

B Time course of glucose concentration.

C Specific glucose (Glc) uptake rate and specific productivity for acetate (Ace).

D Yield of Ace.

Data information: The reaction rates in (C) were calculated for the exponential phase under assumption of quasi-steady state. Because changes in glucose and acetate are rather small during cultivation of the HC ATPase strain, a higher initial biomass concentration of 0.1 gDW/l was used for this strain to get data that are more robust for calculating the metabolite exchange rates. The means (A and B) and the means and individual data (C and D) for $n = 3$ biologically independent samples are shown. The error bars represent ± SD.
Source data are available online for this figure.

© 2021 The Authors

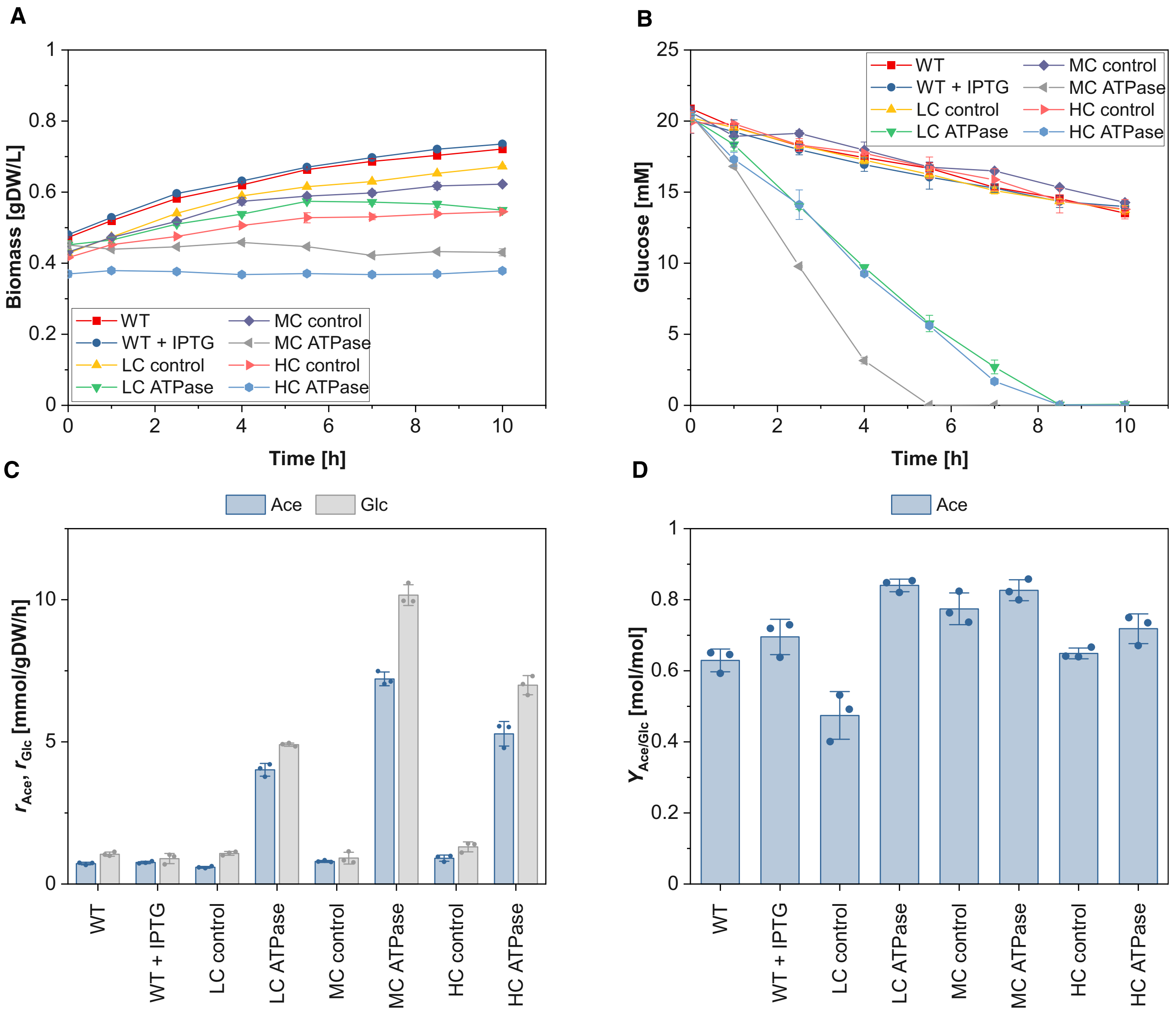

Figure EV2. Aerobic cultivation of the different strains under growth arrest.

A Time course of biomass concentration.
B Time course of glucose concentration.
C Specific glucose (Glc) uptake rate and specific productivity for acetate (Ace).
D Yield of Ace.

Data information: The reaction rates in (C) were calculated from the beginning of cultivation until the last sampling time point where glucose was still present in the medium. The means (A and B) and the means and individual data (C and D) for $n = 3$ biologically independent samples are shown. The error bars represent ± SD. Note: although no nitrogen source was present in the medium, some minor growth (especially of the control and wild-type strains) remained (A), which is a known phenomenon within the first hours of cultivation after nitrogen depletion (Switzer *et al*, 2020).
Source data are available online for this figure.

 © 2021 The Authors

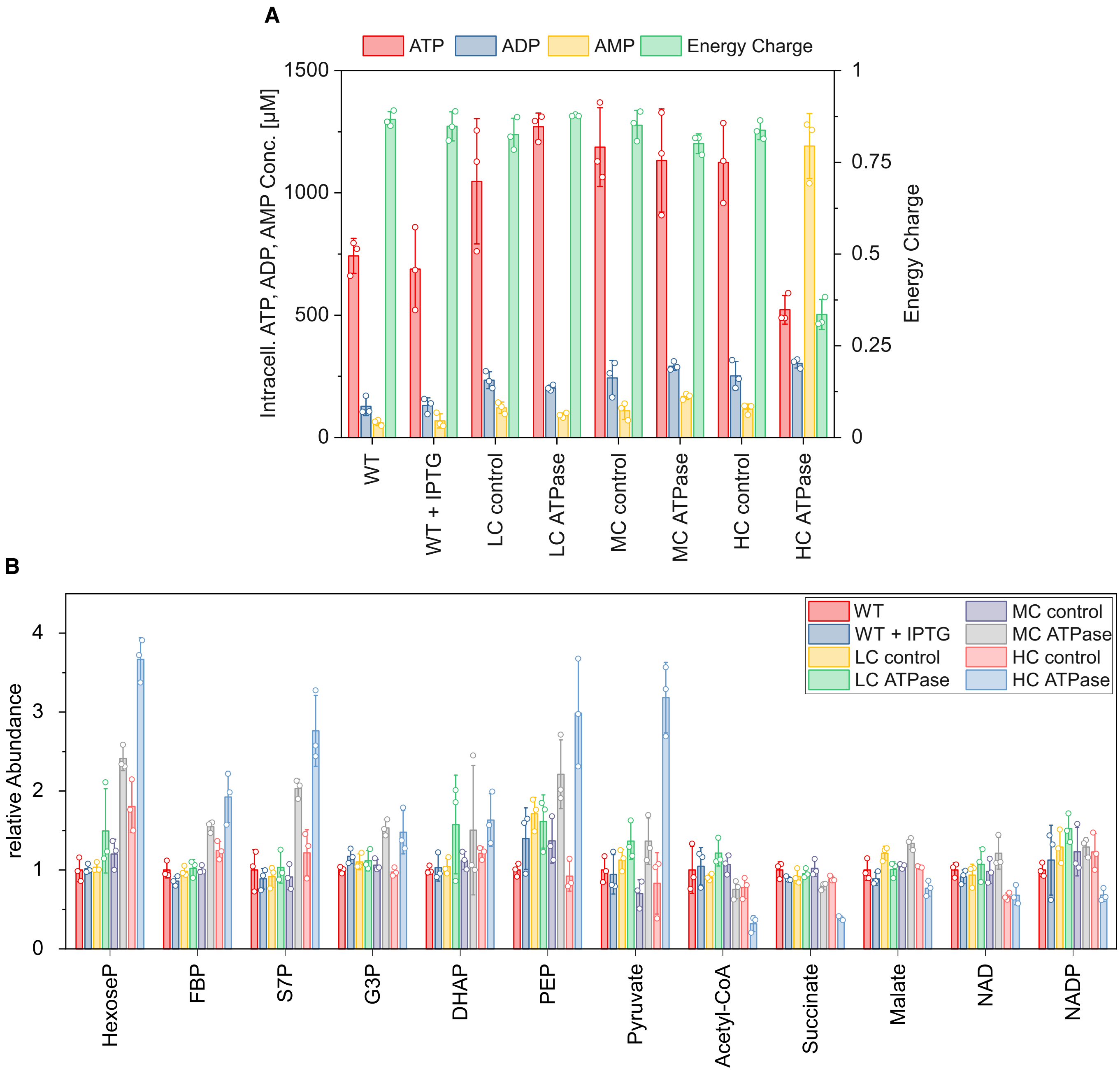

Figure EV3. Intracellular metabolite concentrations of the different strains during anaerobic growth.

A Absolute intracellular ATP, ADP, and AMP concentrations and energy charge.

B Relative intracellular metabolite concentrations from core metabolism.

Data information: The means and individual data of $n = 3$ biologically independent samples are shown and the error bars represent ± SD.

© 2021 The Authors

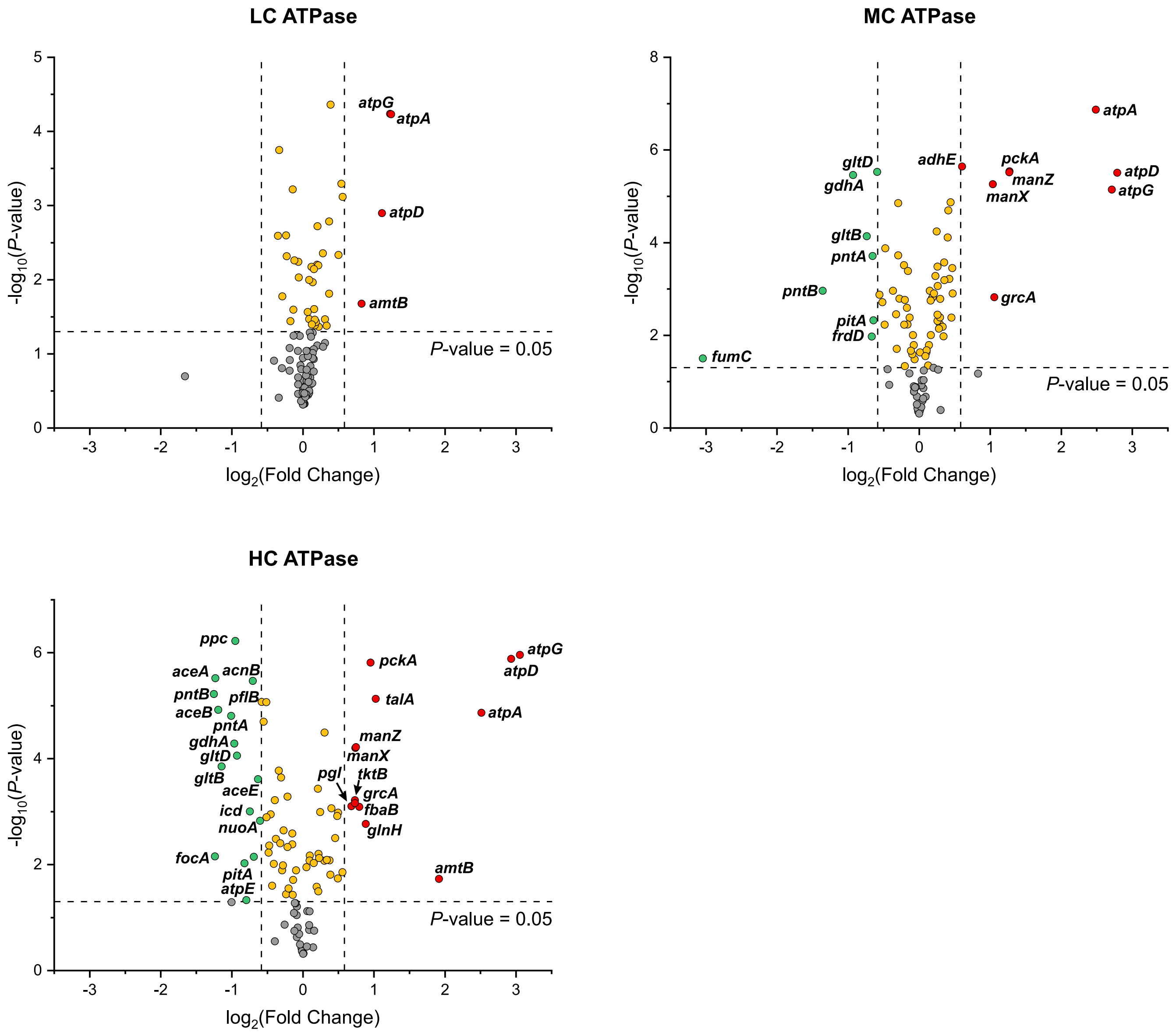

Figure EV4. Changes on proteome level (only proteins from core metabolism are shown) of the three ATPase strains in regard to the corresponding control strains under anaerobic cultivation conditions with growth.

Significantly (*P* value < 0.05) upregulated (upregulation > 1.5-fold) proteins are depicted in red, significantly (*P* value < 0.05) downregulated (downregulation > 0.33-fold) proteins are depicted in green. Proteins with a significant down- or upregulation but below the thresholds of 1.5-fold up- or 0.33-fold downregulation are depicted in yellow, proteins with no significant change (*P* value > 0.05) are depicted in grey. The gene names of the corresponding proteins are given for significantly down- or upregulated proteins. *P* values were calculated for a two-sample *t*-test from *n* = 3 biologically independent samples.

© 2021 The Authors

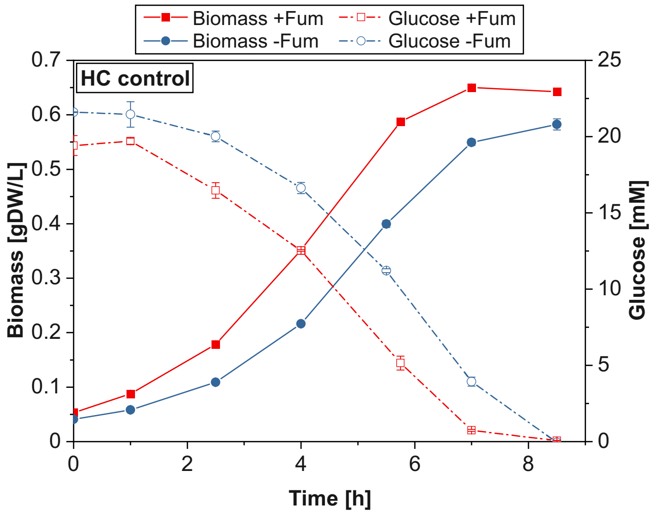

Figure EV5. Anaerobic growth of the HC control (left) and HC ATPase (right) strains with (red) and without (blue) fumarate addition.

The average time courses of biomass and glucose concentrations of n = 3 (−Fum) and n = 2 (+Fum) biologically independent samples are shown. The error bars represent ± SD. See also Appendix Table S4 for the determined specific rates.

Source data are available online for this figure.
