# Supplementary material for: Deciphering the physiological response of Escherichia coli under high ATP demand
Source: Mol Syst Biol. 2021 Dec 20;17(12):e10504. doi: 10.15252/msb.202110504 (PMC8686765; doi:10.15252/msb.202110504)
Supplement: Supplementary file 8 [file MSB-17-e10504-s008.pdf]

Deciphering the Physiological Response of Escherichia coli Under High ATP Demand

Simon Boecker, Giulia Slaviero, Thorben Schramm, Witold Szymanski, Ralf Steuer, Hannes Link, and Steffen Klamt
DOI: 10.15252/msb.202110504

Corresponding author: Steffen Klamt (klamt@mpi-magdeburg.mpg.de)

Review Timeline:

Submission Date:	15th Jun 21
Editorial Decision:	22nd Jul 21
Revision Received:	30th Jul 21
Editorial Decision:	2nd Aug 21
Revision Received:	11th Oct 21
Editorial Decision:	15th Nov 21
Revision Received:	30th Nov 21
Accepted:	30th Nov 21

Editor: Jingyi Hou

Transaction Report:

(Note: With the exception of the correction of typographical or spelling errors that could be a source of ambiguity, letters and reports are not edited. Depending on transfer agreements, referee reports obtained elsewhere may or may not be included in this compilation. Referee reports are anonymous unless the Referee chooses to sign their reports.)

22nd Jul 2021
RE: Manuscript MSB-2021-10504, Deciphering the Physiological Response of Escherichia coli Under High ATP Demand

Thank you again for submitting your work to Molecular Systems Biology. We have now heard back from the three reviewers who agreed to evaluate your study. As you will see below, the reviewers raise substantial concerns on your work, which unfortunately preclude the publication of the study in its current form.

While Reviewers #1 and #2 are relatively more supportive, Reviewer #1 still raises a series of concerns about the model. In particular, Reviewer #3 points to substantial issues with respect to the model and thinks that due to these shortcomings, the major conclusions of the study do not seem to be fully supported by the data and analyses. This reviewer rated the "validity of conclusions" and "suitability of publication" as "low".

During our pre-decision cross-commenting process (in which the reviewers are given the chance to make additional comments, including on each other's reports), Reviewer #3 added, "The model is huge: The first question is whether the model structure is correct. Second, the model contains lots of unknown parameters. Just to mention two issues with the first point: (i) The authors use a fixed summed concentration for ATP and ADP as a constraint. Is there no AMP? Is it not likely that under strong perturbation of energy homeostasis also more AMP is formed? Indeed, this is what even their measurements show. (ii) The authors used (as far as I can tell) for all conditions the same vmax values for the enzymes. Vmax values are influenced by changes in enzyme levels. However, by using one condition-independent vmax value this essentially assumes that enzyme levels do not change. However, their experiments show that enzyme levels change. Of course, when plotting log2 changes of enzyme levels, one only sees the strong changes, but I would consider that a 2-fold increase of at least some enzymes to have an effect on the system. This is not considered. And even these model structure question are not an issue, then there is still the uncertainty of the model parameters. The number of model parameters is huge, the estimation process seem to have involved quite a bit of manual tweaking as described in the SText, and the data that went in there was just the physiological data. From my understanding the proteomics and metabolomics data was NOT used in the estimation. Physiological data is surely not sufficient from a practical identifiability point of view to identify the model parameters. Evidence for this point comes from the following: Fitting physiological data to a stoichiometric model (which has much fewer parameters, i.e. namely only the fluxes) is already an issue and fluxes can usually not be identified. Now, here, the others have even model with much more parameters (namely also kinetic parameters). Thus, it simply cannot be that they can identify the parameters. And as such, I have difficulty in trusting conclusions that were derived from such a model developed in this manner."

Under these circumstances and given that the concerns raised by the reviewers are substantial and are unlikely to be addressable within the scope of a major revision, we see no other choice than to return the manuscript with the message that we cannot offer to publish it.

I am sorry that the review of your work did not result in a more favorable outcome on this occasion, but I hope that you will not be discouraged from sending your work to Molecular Systems Biology in the future. In any case, thank you for the opportunity to examine this work.

Reviewer #1:

This study looks at the role of ATP demand on glycolytic flux, growth rate and product formation, and provides many important new insights into the regulation of central metabolism. It is a well-designed, clearly described study that combines experimental data with a kinetic model. I think this is an important paper for the field. I have a number of suggestions for improvements mainly.

line51: holistic is not a word to be used in this context. The understanding is not gained by looking only at the hole.

Line81: the terminology of rate-limiting steps should perhaps be avoided in a systems biology journal, and replaced by rate-controlling, especially in the context of this topic.

Line210: lower ATPase -> higher I suppose

Line 239: why is AMP not in the model? Is that not an even more potent activator of PFK than ADP?

Line 252: Of course the accumulation of FBP does not have to correlate with glycolytic flux if you stretch the system beyond its normal regulatory boundaries, which you clearly do in HC. Also in line 485, it is unfair to compare your highly arteficial strain with expectations of optimality.
To explain accumulation of FBP in HC I miss reference to the redox state and GAPDH as a clear potential bottleneck of glycolysis. Your NAD levels are lower (but also in HC control?), so possibly the NADH/NAD ratio is higher, inhibiting GAPDH (and explaining high lactate by higher NADH for LDH?). The redox state is notoriously difficult to model properly, but instead of your changes in PFL and PYK, could changes in NADH/NAD ratios (e.g. inhibiting GAPDH instead of PFL and PYK) also improve the model?

Line 373: lactate yield dropped even more in PflB (which makes much more sense), why is this not highlighted? I find the description and especially the explanation of the Table 1 results in general rather poor, and I am missing a direct correspondence with the modeling results. Does the model explain the changes in flux as well? That would be quite impressive.

Fig. 1 and 2. Panels C and D are not very insightful, please change into a Table with the actual numbers. I also suggest to provide the data of A and B on log scale, and I also suggest to additionally plot glucose against biomass: this gives a direct readout of the stoichiometry (yield) of growth, which should change if ATP expenditure increases.

Supplements: ATPase reaction (ATPM) in the model seems crucial. Should it not be a function of demand processes, and relatively insensitive to ATP? I think the Hill function sort of achieves that (and I would prefer to give that rationale with the equation, perhaps citing Hofmeyr or Reich and Sel'kov), but with the parameters used the line is close to k*ATP. Based also on the data of ATP in Fig S4, I would suspect you can describe your data much better by a lower Km and and higher Hill coefficient of the ATPase reaction. By plotting the ATPM flux as a function of the measured ATP (or ATP/ADP, or energy charge, many options), you may be able to get the apparent rate equation. Have you tried? Moreover, what happens if you make the ATPM also a function of mu, as would be biologically relevant? Would you be able to compare growth versus non-growth?

Reviewer #2:

Summary
This study analyzed the impact of varying levels of ATP turnover in E. coli by overexpressing genes of ATP hydrolyzing ATPase under anaerobic and aerobic conditions. The results were compared growth results, metabolomic and proteomic data to a kinetic model of E. coli central metabolism. The authors observed a biphasic behavior of glucose uptake in response to ATP demand. They show that this biphasic behavior is induced by the dependency of the phosphofructokinase on ATP and ADP.

General remarks
This is a well designed study and the results were convincing and revealing of mechanistic and metabolic principals that can guide further metabolic engineering of E. coli. The ability to directly test a concept with both extensive experimental and modeling approaches is unique and compelling. The authors also observed that their first few versions of their kinetic model didn't match the observations and likely lacked regulatory elements. They were able to make modifications that qualitatively matched with their findings.The findings will be directly applicable to researchers focused on efficient design of E. coli and other chassis strains for biomanufacturing and pathway expression systems. These results will also be interesting to a growing community of researchers involved with metabolic modeling. I would like to recommend that this manuscript be published without revisions.

Minor points
Table 1 could potentially be reformatted into a graphical display instead of a table. I found the table useful as a reference but doesn't directly communicate a clear take-away message. The authors should consider converting this into a figure or putting it into the supplemental information.

Reviewer #3:

The authors found that upon that upon an increased ATP hydrolysis, E. coli responds with in increased glycolysis, but only up to a point at which the rate through glycolysis collapses and the growth rate as well. The authors find that allosteric regulation of the glycolytic enzyme phosphofructokinase is responsible for this "collapse". Beyond, with a kinetic model, they provide some further insights on E. coli metabolic regulation. The findings (i.e. the response of E. coli upon increased ATP demand and how

cells compensate this (to some extent) by increase in glycolysis) are interesting for researchers studying and working with carbon/energy metabolism of E. coli.
Main concerns:
KINETIC MODEL/PARAMETER ESTIMATION: My main concern refers to the kinetic model, which is key for the finding on the PFK and the other insights generated. This model contains a huge number of unknown parameters. Unfortunately, the whole modeling building approach and the approach that was used to fit the model is incompletely described. For instance, what are the number of unknown parameters, how was the parameter estimation was done, were all experimental conditions fitted together, were the v_max values allowed to vary across conditions, are the parameters actually identifiable from a structural/practical point of view, etc? What is described makes me somewhat worried (i.e. "first model calibration was conducted by hand"; p. 20 of supplementary text). The data to fit the many model parameters is very scares (i.e. only physiological data), compared to the number of parameters. I simply can't see how such limited data can result in a reliable fitting of the many unknown parameters (overfitting?) and whether I at all can trust this kinetic model, which is the basis on which the main conclusions were drawn (i.e. the one on PFK and the ones presented on page 14). Could other models also explain the data? Also, several choices had to be made to assemble this model. One choice refers to constraining the sum of ATP and ADP to a particular value (cf. Supplemental text p. 5). I would be fine with this assumption of AMP concentrations were identical over the different perturbations applied. However, this is not the case (cf. Fig S3A). Also, I do not understand why the authors, who had measured proteome and metabolome data for the strains here, did not use this data in the fitting. Wouldn't this not have delivered a much more trustworthy model? Overall, I have doubts on the solidness of the conclusions that can be drawn with this kinetic model.
PROTEOME ALLOCATION: According to the concept of 'protein allocation', protein overexpression and also changes in growth rates should lead to changes in protein expression. On the basis of their proteome analyses, the authors argue that there are only very few small changes in protein abundances (p. 13), even though they overexpress proteins and also have strains with largely different growth rates. Does this mean that the protein allocation concept is not correct? Or are the proteome measurements inaccurate? Yet, the authors observe small expression changes, but as far as I can see these expression changes were not used in the model. I would expect a 2-fold change of some enzymes (as the authors have found them in the proteomics analysis) to have some effect on the outcome of the model. I am puzzled why this has not been taken along (see previous point) and whether I then can trust the model.
DETERMINATION OF PHYSIOLOGICAL PARAMETERS: The physiological parameters are the key input data for their work. Yet, I have some concerns about the manner how these data have been determined. First, the authors only determine the uptake/excretion rates on the basis of two measurement time points. This approach is fine if the two time points really originate from the exponential growth phase AND if the measurement data does not have any measurement noise. However, looking at Fig. 1a, I am not sure whether over the full time period cells are in an exponential phase and I do not know at which two time points the authors took their samples to determine the uptake and excretion rates. I think for a work that builds so heavily on physiological data, it would have been much better if the authors had acquired time courses on the extracellular metabolites and had fitted this data to an ODE-based model to estimate the rates. This approach would also have dealt with the issue of measurement noise much better. Second, I am worried about the fact that the authors have not directly measured the biomass dry weight, but have only inferred this from OD measurements by assuming a constant conversion factor between OD and dry weight. At different conditions, it is possible that cell sizes changes or that otherwise an assumption of a constant conversion factor is incorrect. While these two points seem minor, I would like to highlight again that the model only builds on the physiological data, and as such it is even more crucial that the data is 100% solid.
Minor:
1) For quite some time during the reading, it wasn't clear to me whether the observations you describe are observations after dynamic perturbations (i.e. in the abstract, i.e. "increases", "biophasic response curve"; these terms all contain some elements of "time", which would rather be consistent with a dynamic perturbation). You might want to change the wording (here and throughout the manuscript) such that the reader directly is put on the right track, i.e. on the track on steady-state perturbations, i.e. no DYANMCIS!
2) If I remember correctly, the long-known short term glycolytic oscillations also involved PFK and its regulation by ADP. Could this be connected?
3) Line 149: Why lactate? Is there an explanation for this? Redox?
4) Fig. 2a: It seems that in some of the strains there is still an increase in the biomass, even though cells do not have any nitrogen source. How can this be explained?
5) Figures are not mentioned in the order as they indeed appear in the figure panels. E.g. Fig. 4a is mentioned before Fig. 3a. This is confusing.
6) Fig. 4C: This lumped reaction with ICDH shows NADPH as cofactor. Is this correct? Isn't this enzyme not NADH dependent?
7) Line 353/354: I don't get the statement.
8) I strongly advise the authors to display their results as shown in Table 1 as a figure.
9) While reading the result section I always wondered whether protein allocation changes could also play a role. The authors discuss this (and in fact even show new results about this) in the discussion section. I strongly suggest that the authors move this part into the result section.
10) Why are the ATP/ADP levels only shown in the supplement? Isn't this a key piece of information for the story here?
11) The description of the MFA M&M section is short. Information such as on the size of the metabolic model is missing as well as info on what is actually optimized. Sum of fluxes or ATP hydrolysis? If it is the latter, I think the authors should then rather refer to MAXIMAL ATP turnover rates in their text. Also, it wasn't clear to me what was meant with lines 661-665.
12) The CSICD involves production of CO_2. While several reactions in the kinetic model, involve CO_2 as reactant, the CSICD

reaction does not have CO_2 as product.

Thank you very much for handling our manuscript and for sending us your decision letter. Based on the comments of the reviewers, you decided to reject the manuscript. When reading the reviewer comments in detail, we felt that this decision is based on some misunderstandings and misinterpretations and we therefore kindly ask you to consider our appeal.

The preliminary response letter with point-by-point responses is provided in a separate file.

We are grateful for your consideration.

Sincerely,

2nd Aug 2021

Manuscript Number: MSB-2021-10504R-Q
Title: Deciphering the Physiological Response of Escherichia coli Under High ATP Demand
Author: Simon Boecker
Giulia Slaviero
Thorben Schramm
Witold Szymanski
Hannes Link
Steffen Klamt

Thank you for your message asking us to reconsider our decision regarding your manuscript MSB-2021-10504. I have carefully read your manuscript and the referee reports once again and have also discussed your preliminary point-by-point responses with the editorial team. Based on the outline you provide, we think that the proposed revisions sound reasonable. As such, we would welcome the submission of a revised version for further consideration.

Without reiterating all the points raised in the reviews and your preliminary point-by-point responses, some of the more substantial issues are the following:

- The model construction, as well as the scope and limitations of the model, should be better described and discussed.
- Reviewer #3's concern about the unknown parameters, and other raised issues about the model, need to be carefully clarified and/or addressed.

As you may already know, our editorial policy allows in principle a single round of major revision. It is therefore essential to provide responses to the reviewers' comments that are as complete as possible.

On a more editorial level, we would ask you to address the following issues:

Response to the comments of the reviewers

We thank the reviewers for the careful evaluation of our manuscript and their comments. Below, we outline how we addressed the raised points and suggestions of the reviewers.

The changes introduced in the manuscript are highlighted in that document.

Reviewer #1:

Reviewer #1 general comment: This study looks at the role of ATP demand on glycolytic flux, growth rate and product formation, and provides many important new insights into the regulation of central metabolism. It is a well-designed, clearly described study that combines experimental data with a kinetic model. I think this is an important paper for the field. I have a number of suggestions for improvements mainly.

Author response: We thank the reviewer for the very positive overall assessment.

Reviewer #1 comment 1: line51: holistic is not a word to be used in this context. The understanding is not gained by looking only at the hole.

Author response: We agree and changed this formulation (holistic comprehensive).

Reviewer #1 comment 2: Line81: the terminology of rate-limiting steps should perhaps be avoided in a systems biology journal, and replaced by rate-controlling, especially in the context of this topic.

Author response: We agree and changed this formulation as suggested by the reviewer.

Reviewer #1 comment 3: Line210: lower ATPase -> higher I suppose

Author response: We actually meant “lower ATPase levels” but we changed this formulation to avoid confusion.

Reviewer #1 comment 4: Line 239: why is AMP not in the model? Is that not an even more potent activator of PFK than ADP?

Author response: No, in contrast to some eukaryotic organisms (e.g., yeast), the PFK1 in *E. coli* is allosterically regulated by ADP, GDP and PEP, but NOT by AMP. See also: https://biocyc.org/gene?orgid=ECOLI&id=6PFK-1-MONOMER

However, we included also AMP as a metabolite (but not as allosteric effector) in the model due to a comment of Reviewer #3 (see below).

Reviewer #1 comment 5: Line 252: Of course the accumulation of FBP does not have to correlate with

glycolytic flux if you stretch the system beyond its normal regulatory boundaries, which you clearly do in HC. Also in line 485, it is unfair to compare your highly arteficial strain with expectations of optimality. To explain accumulation of FBP in HC I miss reference to the redox state and GAPDH as a clear potential bottleneck of glycolysis. Your NAD levels are lower (but also in HC control?), so possibly the NADH/NAD ratio is higher, inhibiting GAPDH (and explaining high lactate by higher NADH for LDH?). The redox state is notoriously difficult to model properly, but instead of your changes in PFL and PYK, could changes in NADH/NAD ratios (e.g. inhibiting GAPDH instead of PFL and PYK) also improve the model?

Author response: First of all, we agree that it is “unfair” to expect that the HC ATPase strain would behave normally and show optimal behavior under these extreme conditions. We removed the sentence from line 252 and changed the wording in the referred sentence in the Discussion section to weaken this statement.

Regarding the possible explanation of the reviewer for the high FBP accumulation: in principle, yes, increasing NADH/NAD ratios might inhibit the GAPDH (unfortunately, as the reviewer mentioned, we could not measure NADH as this is notoriously difficult). However, this could then not explain the accumulation of PEP and pyruvate (and possibly also of other glycolytic metabolites between DHAP and PEP, for which we do not have measurements). Our model analysis suggests that there should be regulations acting on PFL and PYK with which (i) the metabolite accumulations up to FBP, (ii) the high lactate production (due to high levels of pyruvate, the substrate of the LDH), and (iii) the reduced production of acetate and ethanol in the HC strain can be explained.

Reviewer #1 comment 6: Line 373: lactate yield dropped even more in PflB (which makes much more sense), why is this not highlighted? I find the description and especially the explanation of the Table 1 results in general rather poor, and I am missing a direct correspondence with the modeling results. Does the model explain the changes in flux as well? That would be quite impressive.

Author response: Yes, the drop in lactate yield was indeed to be expected (and also predicted by the model). We extended the discussion of the results (and included also a new Figure, as suggested by Reviewer #2) to highlight this point. We now also mention that the model predicted the changes in the product yields qualitatively (it is difficult to make a direct quantitative comparison of measured yields and model predictions since we do not know how much the PFL level increased in the strain overexpressing the pflB gene compared to the standard HC ATPase strains).

Reviewer #1 comment 7: Fig. 1 and 2. Panels C and D are not very insightful, please change into a Table with the actual numbers. I also suggest to provide the data of A and B on log scale, and I also suggest to additionally plot glucose against biomass: this gives a direct readout of the stoichiometry (yield) of growth, which should change if ATP expenditure increases.

Author response: We now moved the Tables with the exact values of rates and yields for growth and growth arrest under anaerobic conditions from the Appendix to the main manuscript (now Table 1). As reviewer #2 and #3 seem to prefer a graphics (for example, instead of using Table 1 (now Table2)), we decided to include both, Figure and Table, in the main manuscript.

Reviewer #1 comment 8: Supplements: ATPase reaction (ATPM) in the model seems crucial. Should it not be a function of demand processes, and relatively insensitive to ATP? I think the Hill function sort of achieves that (and I would prefer to give that rationale with the equation, perhaps citing Hofmeyr or Reich and Sel'kov), but with the parameters used the line is close to k*ATP. Based also on the data of ATP in Fig S4, I would suspect you can describe your data much better by a lower Km and and higher Hill coefficient of the ATPase reaction. By plotting the ATPM flux as a function of the measured ATP (or ATP/ADP, or energy charge, many options), you may be able to get the apparent rate equation. Have you tried? Moreover, what happens if you make the ATPM also a function of mu, as would be biologically relevant? Would you be able to compare growth versus non-growth?

Author response: Although it will be clear to the reviewer, let us first explain the role of the ATPM reaction. In many (also constraint-based) models, the ATPM (or NGAM) reaction reflects the consumption of ATP for non-growth associated maintenance (NGAM) processes. While this can be fixed to a single flux value (mmol/gDW/h) in constraint-based models, this cannot be done in kinetic models: if we would fix that flux then it might happen that ATP is consumed faster than it is produced by other reactions in the model and the ATP concentration may become negative. Therefore, in kinetic models, the ATPM reaction must somehow depend on the ATP concentration and it must become zero if there is no ATP (this would also correspond to what happens in the cell). If we would not consider expression of the ATPase, then we could use a kinetics for the ATPM reaction as suggested by the reviewer (namely as a function of demand processes, i.e. with relative insensitivity against ATP for a large range of ATP values which could be achieved with a low Km value and a Hill function with high exponents). Furthermore, it should also be noted that the growth-related consumption of ATP is accounted for by a fixed ATP stoichiometry per gram biomass produced in the growth reaction (see ATP balance in the model description). This growth-related demand of ATP was computed from a stoichiometric model as explained in the Appendix. It thus would make no sense to make the ATPM flux a function of mu. (Note also that, again, the growth rate kinetics depends explicitly on ATP and the growth rate will become zero if the ATP concentration is zero).

So far, we have described the situation without overexpressing the ATPase. In our original model we have “abused” the ATPM reaction in a sense that it aggregates both the actual non-growth associated ATP demand as well as the ATP consumed by the overexpressed ATPase levels (both reactions have the same net stoichiometry). The ATPase activity was simulated by increasing the vmax of the ATPM flux: each strain had a characteristic vmax level for the ATPM reaction. For the wild type we adjusted the vmax value of the ATPM reaction such that the ATPM flux comes close to the known wild type ATPM demand (~ 3mmol/gDW/h; this corresponds to the case where no ATPase is expressed). Now, for the aggregated processes, we cannot use the suggested kinetics of a demand process since this would not correctly describe the activity of the ATPase.

However, in the revised model version what we now did is to split the ATPM reaction into two reactions: one for the NGAM demand of ATP (= classical ATPM) and one for the ATPase activity (which would be zero in the wild type). This allows us to really describe the (new) NGAM reaction as a demand process, as suggested by the reviewer.

Reviewer #2:

Reviewer #2 general comment: This is a well designed study and the results were convincing and revealing of mechanistic and metabolic principals that can guide further metabolic engineering of E. coli. The ability to directly test a concept with both extensive experimental and modeling approaches is unique and compelling. The authors also observed that their first few versions of their kinetic model didn't match the observations and likely lacked regulatory elements. They were able to make modifications that qualitatively matched with their findings.The findings will be directly applicable to researchers focused on efficient design of E. coli and other chassis strains for biomanufacturing and pathway expression systems. These results will also be interesting to a growing community of researchers involved with metabolic modeling. I would like to recommend that this manuscript be published without revisions.

Author response: We thank the reviewer for the positive general evaluation of our manuscript.

Reviewer #2 comment 1: Table 1 could potentially be reformatted into a graphical display instead of a table. I found the table useful as a reference but doesn't directly communicate a clear take-away message. The authors should consider converting this into a figure or putting it into the supplemental information.

Author response: We now added a graphical display additionally to the Table. As reviewer #1 prefers a Table with the exact values, we decided to include both, Figure and Table, to the main manuscript.

Reviewer #3:

Reviewer #3 general comment: The authors found that upon that upon an increased ATP hydrolysis, E. coli responds with in increased glycolysis, but only up to a point at which the rate through glycolysis collapses and the growth rate as well. The authors find that allosteric regulation of the glycolytic enzyme phosphofructokinase is responsible for this "collapse". Beyond, with a kinetic model, they provide some further insights on E. coli metabolic regulation. The findings (i.e. the response of E. coli upon increased ATP demand and how cells compensate this (to some extent) by increase in glycolysis) are interesting for researchers studying and working with carbon/energy metabolism of E. coli.

Author response: We thank the reviewer for his comments and give a detailed response below.

Reviewer #3, Comments from pre-decision cross-commenting: "The model is huge: The first question is whether the model structure is correct. Second, the model contains lots of unknown parameters. Just to mention two issues with the first point: (i) The authors use a fixed summed concentration for ATP and ADP as a constraint. Is there no AMP? Is it not likely that under strong perturbation of energy homeostasis also more AMP is formed? Indeed, this is what even their measurements show. (ii) The authors used (as far as I can tell) for all conditions the same vmax values for the enzymes. Vmax values

are influenced by changes in enzyme levels. However, by using one condition-independent vmax value this essentially assumes that enzyme levels do not change. However, their experiments show that enzyme levels change. Of course, when plotting log2 changes of enzyme levels, one only sees the strong changes, but I would consider that a 2-fold increase of at least some enzymes to have an effect on the system. This is not considered. And even these model structure question are not an issue, then there is still the uncertainty of the model parameters. The number of model parameters is huge, the estimation process seem to have involved quite a bit of manual tweaking as described in the SText, and the data that went in there was just the physiological data. From my understanding the proteomics and metabolomics data was NOT used in the estimation. Physiological data is surely not sufficient from a practical identifiability point of view to identify the model parameters. Evidence for this point comes from the following: Fitting physiological data to a stoichiometric model (which has much fewer parameters, i.e. namely only the fluxes) is already an issue and fluxes can usually not be identified. Now, here, the others have even model with much more parameters (namely also kinetic parameters). Thus, it simply cannot be that they can identify the parameters. And as such, I have difficulty in trusting conclusions that were derived from such a model developed in this manner."

Arguably, the most serious concerns of the reviewer are related to the identifiability of the parameters of the model and the value of the model (and its conclusions) under non-identifiability and parameter uncertainty, as reflected by this statement:

"Thus, it simply cannot be that they can identify the parameters. And as such, I have difficulty in trusting conclusions that were derived from such a model developed in this manner."

This is also reflected by the following statement taken from the main concern 1 of this Reviewer (see below):

"I simply can't see how such limited data can result in a reliable fitting of the many unknown parameters (overfitting?) and whether I at all can trust this kinetic model, which is the basis on which the main conclusions were drawn ..."

The statements of the reviewer express the concern that a model is generally only useful if all parameters are identifiable. But this is not true and undermines that models with non-identifiable parameters can be very valuable! Let us start with a very simple example to illustrate this point: assume there is a (algebraic) model that states Y=a*b*X with unknown parameters a and b. Given measurements of Y and X we might be able to demonstrate that this model is able to reasonably reflect the measured data and we can then find a unique estimate for the value of the product of a*b. Clearly, the individual values for a and b are not identifiable (the estimation will deliver values for a and b such that a*b gives an optimal fit; but a and b are clearly non-unique). But the model still has a great value: it could be falsified if there is no linear relationship between X and Y, and, if there is a linear relationship, the (non-unique) estimated values of a and b will lead to a model that can predict the behavior of Y given X. The non-identifiability of the parameters a and b is simply not relevant for this procedure.

In our study, we built, in a bottom-up manner, a kinetic model of the central metabolism of E.coli for anaerobic conditions and used experimental data to calibrate it. The reviewer is right that, with the relatively large number of parameters (179 in model version 2; thereof 101 unknown parameters to be estimated) and the relatively small set of experimental data, there will be parameters that are not identifiable - as in most other kinetic models of this size. This would indeed be crucial for some model

purposes, for example, if the aim is to uniquely identify certain parameters (e.g., a Km value) or for model selection, where the concrete level of a certain parameter may decide about the existence of certain interactions. However, this was not relevant for our study since the main goal of our model was instead to (i) show that we can build, in a bottom-up manner, a kinetic model that includes basic mechanisms and biological knowledge of the central metabolism of E. coli and that this model can reproduce the experimental data; (ii) to use this model to gain insights regarding the biphasic (steady-state) response curve of the glucose uptake rate and to possibly falsify our hypothesis (if the biphasic response curve cannot be reproduced), and (iii) to make meaningful predictions that can be verified with experiments (and thereby to strengthen the confidence in the model). We (and apparently also reviewers #1 and #2) think that our model indeed fulfilled these goals. It was never our intention to obtain a model, where the parameters are all identifiable with the given measurements (and we never claimed that; but maybe we should make this point clearer; see also below). In our opinion, the reviewer underestimates the value of a model with non-identifiable parameters and demands too much (even to exclude that other models can explain the data – this is, in general, impossible, even for models where all parameters are identifiable). We would like to convince the reviewer to take another perspective: we HAVE a model that is consistent with our hypotheses and can explain the observed behavior and its predictive capabilities could be clearly demonstrated (see MCA analysis with experimental validation). To say it in other words: would the reviewer trust our argumentation (e.g., the effect of the PFK mechanism on the glycolytic flux) more if we would discuss our hypotheses only verbally without providing an integrated model that demonstrates feasibility of our mechanistic explanation while taking into account a large set of metabolic interactions and regulations? The model proves that our argumentation is a possible mechanistic interpretation (of course, this cannot exclude that there might be other models explaining the observations – but, again, NO model can do that!). Clearly, one may argue that a model is only useful if it cannot reproduce arbitrary datasets and if its predictions are, at least qualitatively, robust against parameter variations. Regarding the first point, the fact that the (first version of the) kinetic model (also after several rounds of parameter fitting) could not reflect the measured high concentrations of glycolytic metabolites already demonstrates that our model cannot be calibrated to arbitrary data and observations! The latter point has now been addressed in the revised manuscript by a Monte-Carlo sampling of parameters (see below).

However, having said this, we agree with the criticism of the reviewer that the model calibration as well as the general role of the model in our study and its limitations should be described and discussed in more detail to avoid such misunderstandings. For example, the reviewer assumes that we did not use the metabolome data for parameter fitting, but we did so! We address these issues under the next (more specific) point of the reviewer.

Regarding the issues with (a) the inclusion of AMP in the model and (b) the proteomics data and the used vmax values in the model, see the explanations given below under main concern 1 and main concern 2, respectively.

Reviewer #3 main concern 1: KINETIC MODEL/PARAMETER ESTIMATION: My main concern refers to the kinetic model, which is key for the finding on the PFK and the other insights generated. This model contains a huge number of unknown parameters. Unfortunately, the whole modeling building approach and the approach that was used to fit the model is incompletely described. For instance, what are the number of unknown parameters, how was the parameter estimation was done, were all

experimental conditions fitted together, were the v_max values allowed to vary across conditions, are the parameters actually identifiable from a structural/practical point of view, etc? What is described makes me somewhat worried (i.e. "first model calibration was conducted by hand"; p. 20 of supplementary text). The data to fit the many model parameters is very scares (i.e. only physiological data), compared to the number of parameters. I simply can't see how such limited data can result in a reliable fitting of the many unknown parameters (overfitting?) and whether I at all can trust this kinetic model, which is the basis on which the main conclusions were drawn (i.e. the one on PFK and the ones presented on page 14). Could other models also explain the data? Also, several choices had to be made to assemble this model. One choice refers to constraining the sum of ATP and ADP to a particular value (cf. Supplemental text p. 5). I would be fine with this assumption of AMP concentrations were identical over the different perturbations applied. However, this is not the case (cf. Fig S3A). Also, I do not understand why the authors, who had measured proteome and metabolome data for the strains here, did not use this data in the fitting. Wouldn't this not have delivered a much more trustworthy model? Overall, I have doubts on the solidness of the conclusions that can be drawn with this kinetic model.

Author response:

Parameter identifiability: Regarding the main issue of identifiability of the parameters and the value of a model if parameters are not identifiable, please see our response to the previous point above.

However, we agree that we should discuss in the manuscript that the model has a relatively large number of parameters and that many of them will not be uniquely identifiable – but that the model still has its value (as detailed under the previous point): so we now write in the Discussion section:

"The kinetic model played an important role in this study to identify and analyze potential mechanisms in the metabolism of E. coli that led to the observed phenotypes under high ATP demand. It has to be noted that the model is relatively large and comprises more than 100 hundred unknown parameters, many of which will not be uniquely identifiable, despite fitting the model against a considerable set of data. However, the model is based on well-known biological knowledge of E. coli's central metabolism, it is able to reproduce measurements of the different strains reasonably well and it gave predictions that could be successfully verified. Moreover, the results of the Monte-Carlo sampling of kinetic parameters showed that key properties of the kinetic model and its predictions are robust over a wide range of parameter variations. Hence, despite potential parameter identifiability issues, the model could demonstrate its predictive power and thus represents a solid and plausible basis that supports our hypotheses and explains major findings of this study. However, as is true for every model, we can neither prove its correctness nor that other models with alternative mechanisms may exist that reproduce the observed phenomena equally well."

Parameter estimation procedure: Again, we agree with the reviewer that we needed to extend the description on how we calibrated the model and fitted the unknown parameters. For example, the reviewer assumes that we did not use the metabolome data for parameter fitting, but we did. Accordingly, we included more information in the main document and extended the description of the parameter identification process in the Supplementary Text in the Appendix.

Monte-Carlo sampling of kinetic parameters and robustness of model predictions: Furthermore, to increase the trust of the reviewer in the model and its predictions and to analyze the robustness of the model with respect to parameter uncertainties, we employed the Monte-Carlo approach of Murabito et al. (2014). This approach samples kinetic parameters of a kinetic model, such that the sampled

parameter set (of one run) gives rise to the known reference (steady) state (characterized by the measured steady state metabolic fluxes and metabolite concentrations for that specific scenario). For each specific parameter set, one can then determine important system features, in particular, flux control coefficients. In this way, one obtains a probability distribution of certain model features (such as flux control coefficients) over all sampled parameter sets. We used this approach to analyze the robustness of our model-based FCC predictions for the HC strain and to analyze parameter uncertainties. The results of the Monte-Carlo sampling of kinetic parameters showed that key properties of the kinetic model are robust over a wide range of parameter variations (two order of magnitudes). Hence, despite potential parameter identifiability issues, the model could demonstrate predictive values also under parameter uncertainty and thus represents a solid and plausible basis that supports our hypotheses and explains major findings of this study.

A section on the Monte-Carlo study has been added to the main manuscript and a more detailed description of the entire procedure and its results is given in a new section (4) in the Appendix.

Inclusion of AMP: Finally, regarding AMP, in the original model we did not include AMP because it is not involved in any reaction considered in the network (also not as allosteric regulator, since PFK does not depend on AMP in E. coli). We used therefore the ATP/(ADP+ATP) as a proxy for the energy charge. The reviewer is right that the AMP concentration is largely increased in the HC ATPase strain, which is most likely a consequence of the operation of the adenylate kinase (2 ADP ↔ AMP + ATP) and an(other) indicator that the energy charge is low. However, one can also see that the total concentration of ATP+ADP in the HC ATPase strain is still close to the WT strain. In fact, it is the overall adenosine phosphate pool (AMP+ADP+ATP), which varies in the strains and increases with increasing ATPase activity. This could be a consequence of the reduced growth rate, which might lead to higher accumulation of these central metabolites. However, the largest increase of this pool is not more than 2-fold (HC ATPase vs. WT),

Now, to account for the reviewer's comment, we introduced the following model change to account also for AMP and to enable a better comparison between energy charges of the model with the measurements. We included AMP as metabolite and the adenylate kinase as a reaction, which will connect ADP and ATP levels with AMP. Still, we do not know the precise mechanism that adjusts the size of the total adenosine phosphate pool and, as an approximation, we therefore assumed a conservation relation AMP+ADP+ATP=const. The assumption of a constant overall pool (as done in almost all kinetic models of *E. coli*) should be acceptable, since the maximum change in the size of the pool was only ~2-fold and because it is the relative ratio of the three metabolites that matter for many processes.

Regarding the treatment of proteome measurements and vmax values see our response to the next point (main concern 2).

Reviewer #3 main concern 2: PROTEOME ALLOCATION: According to the concept of 'protein allocation', protein overexpression and also changes in growth rates should lead to changes in protein expression. On the basis of their proteome analyses, the authors argue that there are only very few small changes in protein abundances (p. 13), even though they overexpress proteins and also have strains with largely different growth rates. Does this mean that the protein allocation concept is not

correct? Or are the proteome measurements inaccurate? Yet, the authors observe small expression changes, but as far as I can see these expression changes were not used in the model. I would expect a 2-fold change of some enzymes (as the authors have found them in the proteomics analysis) to have some effect on the outcome of the model. I am puzzled why this has not been taken along (see previous point) and whether I then can trust the model.

Author response: First of all, over the entire proteome, there are indeed some significant changes in the protein abundances – and this is most likely a consequence of protein reallocation. However, as stated in the manuscript, it is also true that glycolytic enzymes are much less affected; the fold-change is less than 1,6 for all these enzymes (this is also consistent with the fact that these enzymes are known to be much less regulated at the gene expression level). In fact, ALL enzyme levels of reactions contained in the model exhibit a less than 1,6-fold change in the HC strain, except for PPC, PCK and MDH (malate dehydrogenase). The case of PPC and PCK was discussed in detail in the manuscript and the case of MDH actually fits nicely to this discussion (it seems that the cell seeks to increase the flux from PEP to Malate via PCK (instead of PPC) to gain one extra ATP). However, the overall flux from PPC/PCK via MDH is apparently still relatively low, as the succinate production is still low also in the HC ATPase strain. For this reason, we think that it is justified to approximate the behavior with constant vmax levels of the enzymes in the model to avoid consideration of different models for each strain (and each experiment). Furthermore, we tested how the fluxes and metabolite concentrations would change if we adapt the vmax values for the HC ATPase strain relatively to the changes in protein abundances compared to the wild type. We see only smaller changes in the fluxes, for example, the simulated glycolytic flux in the HC ATPase strain would in this case slightly reduce to 91 %.

We slightly extended the text in the manuscript to justify the use of constant vmax values.

Reviewer #3 main concern 3: DETERMINATION OF PHYSIOLOGICAL PARAMETERS: The physiological parameters are the key input data for their work. Yet, I have some concerns about the manner how these data have been determined. First, the authors only determine the uptake/excretion rates on the basis of two measurement time points. This approach is fine if the two time points really originate from the exponential growth phase AND if the measurement data does not have any measurement noise. However, looking at Fig. 1a, I am not sure whether over the full time period cells are in an exponential phase and I do not know at which two time points the authors took their samples to determine the uptake and excretion rates. I think for a work that builds so heavily on physiological data, it would have been much better if the authors had acquired time courses on the extracellular metabolites and had fitted this data to an ODE-based model to estimate the rates. This approach would also have dealt with the issue of measurement noise much better. Second, I am worried about the fact that the authors have not directly measured the biomass dry weight, but have only inferred this from OD measurements by assuming a constant conversion factor between OD and dry weight. At different conditions, it is possible that cell sizes changes or that otherwise an assumption of a constant conversion factor is incorrect. While these two points seem minor, I would like to highlight again that the model only builds on the physiological data, and as such it is even more crucial that the data is 100% solid.

Author response: Regarding the determination of the uptake and excretion rates of cells cultivated with nitrogen source, only the exponential growth phases were taken into account, as demanded by the reviewer. The time points considered for exponential growth and used for determining the growth rate are now provided in the Source data (relevant for Figures 1, 5 and EV1). Furthermore, we had

indeed determined time courses for all (external) metabolite concentrations from which we used the first and the last data point within the exponential phase to determine the exchange rates (separately for each biological replicate from which then the mean and standard deviations were determined for each rate). As indicated in Figures 1 and 2 and in Tables 1 and 2, we observed generally relatively low standard deviations in the calculated growth and exchange rates indicating a low uncertainty in the determined rates. We now included a paragraph in the Methods section describing in more detail how the growth rate was determined for exponentially growing cells.

Regarding the OD measurements: Due to an additional significant experimental effort, using a constant conversion factor for estimating the biomass dry weight from OD is a common practice in our as well as in many other labs, also for different conditions/mutant strains (e.g. doi:10.1038/msb.2013.66; doi:10.1016/j.ymben.2020.03.004; doi:10.1038/npjsba.2016.35). However, to verify that there are no significant differences in the conversion factor between the different strains, we have now repeated the experiments for determining the conversion factor for the WT MG1655 and the HC ATPase strain (as these two strains show the biggest difference in their growth behavior) for anaerobic growth. We detected no significant difference between these two strains (MG1655: 0.189 gDW/L/OD_{420}; 0.191 gDW/L/OD_{420}; 0.219 gDW/L/OD_{420}. HC ATPase: 0.196 gDW/L/OD_{420}; 0.212 gDW/L/OD_{420}; 0.217 gDW/L/OD_{420}. P value = 0.4929) nor a larger discrepancy to the conversion factor used in this study (0.22), which was previously determined in our lab.

Reviewer #3 minor comment 1: For quite some time during the reading, it wasn't clear to me whether the observations you describe are observations after dynamic perturbations (i.e. in the abstract, i.e. "increases", "biophasic response curve"; these terms all contain some elements of "time", which would rather be consistent with a dynamic perturbation). You might want to change the wording (here and throughout the manuscript) such that the reader directly is put on the right track, i.e. on the track on steady-state perturbations, i.e. no DYANMCIS!

Author response: Indeed, in our study, observations to perturbations (including response curves) correspond to the (new) steady state (flux or concentration) values after inducing the respective perturbations and, as the reviewer stated, our analyses focus clearly on steady state. As suggested by the reviewer, we made this clearer in the manuscript. In particular, we included “steady-state” as attribute at several places (e.g. “biphasic steady-state response curve” in Fig.3, but not only there)

Reviewer #3 minor comment 2: 2) If I remember correctly, the long-known short term glycolytic oscillations also involved PFK and its regulation by ADP. Could this be connected?

Author response: Glycolytic oscillations were mainly observed in S. cerevisiae (partially also in extracts of yeast) and were indeed attributed to the regulation of PFK (but there with AMP as allosteric regulator, not ADP). Much less evidence of oscillating glycolytic fluxes has been reported for E. coli, but it could be that the complex mechanism at PFK may also give rise to oscillations in E. coli.

Reviewer #3 minor comment 3: Line 149: Why lactate? Is there an explanation for this? Redox?

Author response: It is likely the same reason as for the observed increased lactate excretion in the HC ATPase strain under anaerobic growth (see detailed discussion on PFL and PYK in the Results section).

Reviewer #3 minor comment 4: Fig. 2a: It seems that in some of the strains there is still an increase in the biomass, even though cells do not have any nitrogen source. How can this be explained?

Author response: It is a known phenomenon that E. coli can continue cell growth within the first hours after nitrogen depletion (see: Switzer A, Burchell L, McQuail J, Wigneshweraraj S (2020) The Adaptive Response to Long-Term Nitrogen Starvation in Escherichia coli Requires the Breakdown of Allantoin. J Bacteriol 202: e0017220). We cited this reference in the caption of Figure S2 (now Figure EV2) but included this reference now also in the main manuscript.

Reviewer #3 minor comment 5: Figures are not mentioned in the order as they indeed appear in the figure panels. E.g. Fig. 4a is mentioned before Fig. 3a. This is confusing.

Author response: Figure 3a (line 181) is referenced before Figure 4a (line 197) (the lines may have changed in the revised manuscript).

Reviewer #3 minor comment 6: Fig. 4C: This lumped reaction with ICDH shows NADPH as cofactor. Is this correct? Isn't this enzyme not NADH dependent?

Author response: In E. coli, the ICDH is indeed (mainly) NADPH-dependent.

Reviewer #3 minor comment 7: Line 353/354: I don't get the statement.

Author response: We rephrased this sentence.

Reviewer #3 minor comment 8: I strongly advise the authors to display their results as shown in Table 1 as a figure.

Author response: We agree and added a graphical display (new Figure 5). As reviewer #1 prefers Tables with the exact values, we decided to include both, Figure and Table, to the main manuscript.

Reviewer #3 minor comment 9: While reading the result section I always wondered whether protein allocation changes could also play a role. The authors discuss this (and in fact even show new results about this) in the discussion section. I strongly suggest that the authors move this part into the result section.

Author response: Yes, we wanted to discuss the issue of protein (re)allocation potentially induced by the ATPase overexpression and how this may have contributed to the observed phenotypes and changes, especially in the HC ATPase strain. However, since protein reallocation is rather a potential artefact of the ATPase overexpression (in contrast to the intended effect of ATP consumption) we would like to keep that part in the Discussion section, to not distract the reader from the key message (otherwise we would also need to start a detailed discussion on those issues in the Results section, which actually belongs to the Discussion section). Moreover, the results of the additional experiment clearly excluded a significant effect of ATPase expression of protein (reallocation) and should therefore not get too much space in the main text.

Reviewer #3 minor comment 10: Why are the ATP/ADP levels only shown in the supplement? Isn't this a key piece of information for the story here?

Author response: We already have quite a number of large figures and Tables in the main text (now even one Figure and one Table more in the revised version). However, Figure 4 displays the measured (and now also the model-predicted) energy charge, which provides a meaningful condensed representation of the ATP/ADP/AMP data.

Reviewer #3 minor comment 11: The description of the MFA M&M section is short. Information such as on the size of the metabolic model is missing as well as info on what is actually optimized. Sum of fluxes or ATP hydrolysis? If it is the latter, I think the authors should then rather refer to MAXIMAL ATP turnover rates in their text. Also, it wasn't clear to me what was meant with lines 661-665.

Author response: Information on the stoichiometric model (and the model itself) used in this study is now also available from the Github repository (where also the kinetic models are deposited), together with a detailed description how the ATPase fluxes were calculated from the experimentally determined exchange rates. The text on the ATPM and ATPase flux calculations was also revised to clarify the used procedure.

Reviewer #3 minor comment 12: The CSICD involves production of CO2. While several reactions in the kinetic model, involve CO2 as reactant, the CSICD reaction does not have CO2 as product.

Author response: Thanks, we included CO2 in the reaction kinetics. This did not have any crucial effect since the CO2 concentration is fixed in the model.

15th Nov 2021

Manuscript Number: MSB-2021-10504RR
Title: Deciphering the Physiological Response of Escherichia coli Under High ATP Demand
Author: Simon Boecker
Giulia Slaviero
Thorben Schramm
Witold Szymanski
Ralf Steuer
Hannes Link
Steffen Klamt

Thank you for sending us your revised manuscript. We have now heard back from the two reviewers who were asked to evaluate your study. As you will see, the reviewers are overall satisfied with the modifications made. Before we can formally accept your manuscript, we would ask you to address the following issues:

1. Reviewer #3 provided constructive suggestions and comments on the manuscript's structure, which we would strongly encourage you to consider and address within reason.

Reviewer #1:

I am content with the responses to my comments and believe this paper is an important contribution to the field. I also support the position of the authors wrt the criticism of reviewer 3 and fully agree that a model can be useful and provide important insights without the structure and all parameters being known. It depends on the research question. They provide a parameter scan that provides confidence in the robustness of their predictions against local parameter uncertainty. In view of the question that they pose, and the experimental follow up of their predictions, I believe their modeling approach is well justified.

Reviewer #3:

I understand the authors' point. Having re-read the manuscript, I think I now understand better of what is/was my problem with the story. I think my problem is/was that after the finding of the biphasic behavior, the authors directly jumped into the large kinetic model. Instead, I feel that the authors could take the material that they have, reorder it a bit and develop their story in a more step-wise manner, where the big kinetic models would just come after the metabolomics and proteomics data and the small PFK model. For instance:

- Start with observation on the biphasic behavior (as you do know)
- Mention your hypothesis on what could explain the behavior (i.e. the dependency of PFK on ADP and ATP), i.e. lines 220ff
- State that for hypothesis to be true what your expectation is on ADP and ATP levels in the different perturbation strains; test this with metabolomics experiment, i.e. bring lines 262ff; this data shows that the metabolomics data would be consistent with the hypothesis
- Test the hypothesis further with the small model of PFK reaction only, i.e. lines 239; also this shows that the hypothesis would be consistent

However, for a further testing of the hypothesis a bigger kinetic model is needed, because ....; towards developing this model,

- we needed to know whether changes in protein concentrations needed to be modeled as well, and this is why we did proteomics. Here, we found that protein levels don't change much, and thus we considered them to be constant

- First kinetic model; i.e. lines 204 ... Conclusion: This model cannot represent the data; something is missing ....
- Second kinetic model, ... Find what was missing...
- Test predictions:

o First test robustness of predictions with MC analysis
o Then, once the robustness is confirmed, test predictions experimentally

This structure would have several advantages:

- It goes from simple to more complex (i.e. kinetic model), from experimental validation of observation (i.e. metabolomics, ATP, ADP levels) to model-based validation of hypothesis
- It brings the metabolomics data early (now you need the data for the kinetic model but in fact only later show the data)
- It avoids reactions of readers such as my earlier one on the huge kinetic model; as in the above mentioned suggested structure the model only comes at the end as "final" confirmation
- Also, as you would bring the proteomics data before the development of the kinetic model, a reader has seen the proteomics data and thus is "convinced" that protein expression changes would not need to be taken into account in the model

I sincerely hope that the authors consider such an alternative structure. I recently had a similar experience with a manuscript of my lab, where a reviewer had "forced" us to rethink the story line. After initial significant grinding of teeth, I have to confess that the re-write was absolutely worth it and the story that we resubmitted was much better than the one we had before. I hope I can help the authors of this manuscript to rethink their story line as well. Ultimately, if the story line is improved, the manuscript will be appreciated more by readers and thus will have increased impact.

Reviewer #1:

I am content with the responses to my comments and believe this paper is an important contribution to the field. I also support the position of the authors wrt the criticism of reviewer 3 and fully agree that a model can be useful and provide important insights without the structure and all parameters being known. It depends on the research question. They provide a parameter scan that provides confidence in the robustness of their predictions against local parameter uncertainty. In view of the question that they pose, and the experimental follow up of their predictions, I believe their modeling approach is well justified.

We thank the reviewer for this very positive final statement.

Reviewer #3:

I understand the authors' point. Having re-read the manuscript, I think I now understand better of what is/was my problem with the story. I think my problem is/was that after the finding of the biphasic behavior, the authors directly jumped into the large kinetic model. Instead, I feel that the authors could take the material that they have, reorder it a bit and develop their story in a more step-wise manner, where the big kinetic models would just come after the metabolomics and proteomics data and the small PFK model. For instance:

- Start with observation on the biphasic behavior (as you do know)

- Mention your hypothesis on what could explain the behavior (i.e. the dependency of PFK on ADP and ATP), i.e. lines 220ff

- State that for hypothesis to be true what your expectation is on ADP and ATP levels in the different perturbation strains; test this with metabolomics experiment, i.e. bring lines 262ff; this data shows that the metabolomics data would be consistent with the hypothesis

- Test the hypothesis further with the small model of PFK reaction only, i.e. lines 239; also this shows that the hypothesis would be consistent

- However, for a further testing of the hypothesis a bigger kinetic model is needed, because ....; towards developing this model, we needed to know whether changes in protein concentrations needed to be modeled as well, and this is why we did proteomics. Here, we found that protein levels don't change much, and thus we considered them to be constant

- First kinetic model; i.e. lines 204 ... Conclusion: This model cannot represent the data; something is missing ....

- Second kinetic model, ... Find what was missing...

- Test predictions:

o First test robustness of predictions with MC analysis

o Then, once the robustness is confirmed, test predictions experimentally

This structure would have several advantages:

- It goes from simple to more complex (i.e. kinetic model), from experimental validation of observation (i.e. metabolomics, ATP, ADP levels) to model-based validation of hypothesis

- It brings the metabolomics data early (now you need the data for the kinetic model but in fact only later show the data)

- It avoids reactions of readers such as my earlier one on the huge kinetic model; as in the above mentioned suggested structure the model only comes at the end as "final" confirmation

- Also, as you would bring the proteomics data before the development of the kinetic model, a reader has seen the proteomics data and thus is "convinced" that protein expression changes would not need to be taken into account in the model

I sincerely hope that the authors consider such an alternative structure. I recently had a similar experience with a manuscript of my lab, where a reviewer had "forced" us to rethink the story line. After initial significant grinding of teeth, I have to confess that the re-write was absolutely worth it and the story that we resubmitted was much better than the one we had before. I hope I can help the authors of this manuscript to rethink their story line as well. Ultimately, if the story line is improved, the manuscript will be appreciated more by readers and thus will have increased impact.

We thank the reviewer for this suggestion and restructured the manuscript exactly as recommended. Indeed, we also feel that the logical flow of arguments has been significantly improved by this modification. Note that we had to split Figure 3 into three separate Figures (now 7 figures in total). Except for restructuring (+ streamlining the text), no other major changes were introduced.

30th Nov 2021
Manuscript number: MSB-2021-10504RRR
Title: Deciphering the Physiological Response of Escherichia coli Under High ATP Demand

Thank you again for sending us your revised manuscript. We are now satisfied with the modifications made and I am pleased to inform you that your paper has been accepted for publication.

EMBO PRESS

YOU MUST COMPLETE ALL CELLS WITH A PINK BACKGROUND

PLEASE NOTE THAT THIS CHECKLIST WILL BE PUBLISHED ALONGSIDE YOUR PAPER

Corresponding Author Name: Steffen Klamt

Journal Submitted to: Molecular Systems Biology

Manuscript Number: MSB-2021-10504

USEFUL LINKS FOR COMPLETING THIS FORM

http://www.antibodypedia.com
http://1degreebio.org
http://www.equator-network.org/reporting-guidelines/improving-bioscience-research-repor

http://grants.nih.gov/grants/olaw/olaw.htm
http://www.mrc.ac.uk/Ourresearch/Ethicsresearchguidance/Useofanimals/index.htm
http://ClinicalTrials.gov
http://www.consort-statement.org
http://www.consort-statement.org/checklists/view/32-consort/66-title
http://www.equator-network.org/reporting-guidelines/reporting-recommendations-for-tumo
http://datadryad.org
http://figshare.com
http://www.ncbi.nlm.nih.gov/gap
http://www.ebi.ac.uk/ega
http://biomodels.net/
http://biomodels.net/miriam/
http://jjj.biochem.sun.ac.za
https://osp.od.nih.gov/biosafety-biosecurity-and-emerging-biotechnology/
http://www.selectagents.gov/

Reporting Checklist For Life Sciences Articles (Rev. June 2017)

This checklist is used to ensure good reporting standards and to improve the reproducibility of published results. These guidelines are consistent with the Principles and Guidelines for Reporting Preclinical Research issued by the NIH in 2014. Please follow the journal's authorship guidelines in preparing your manuscript.

A- Figures

1. Data

The data shown in figures should satisfy the following conditions:

- the data were obtained and processed according to the field's best practice and are presented to reflect the results of the experiments in an accurate and unbiased manner.
- figure panels include only data points, measurements or observations that can be compared to each other in a scientifically meaningful way.
- graphs include clearly labeled error bars for independent experiments and sample sizes. Unless justified, error bars should not be shown for technical replicates.
- if n< 5, the individual data points from each experiment should be plotted and any statistical test employed should be justified
- Source Data should be included to report the data underlying graphs. Please follow the guidelines set out in the author ship guidelines on Data Presentation.

2. Captions

Each figure caption should contain the following information, for each panel where they are relevant:

- a specification of the experimental system investigated (eg cell line, species name).
- the assay(s) and method(s) used to carry out the reported observations and measurements
- an explicit mention of the biological and chemical entity(ies) that are being measured.
- an explicit mention of the biological and chemical entity(ies) that are altered/varied/perturbed in a controlled manner.

- the exact sample size (n) for each experimental group/condition, given as a number, not a range;
- a description of the sample collection allowing the reader to understand whether the samples represent technical or biological replicates (including how many animals, litters, cultures, etc.).
- a statement of how many times the experiment shown was independently replicated in the laboratory.
- definitions of statistical methods and measures:
  - common tests, such as t-test (please specify whether paired vs. unpaired), simple $\chi 2$ tests, Wilcoxon and Mann-Whitney tests, can be unambiguously identified by name only, but more complex techniques should be described in the methods section;
  - are tests one-sided or two-sided?
  - are there adjustments for multiple comparisons?
  - exact statistical test results, e.g., P values = x but not P values < x;
  - definition of 'center values' as median or average;
  - definition of error bars as s.d. or s.e.m.

Any descriptions too long for the figure legend should be included in the methods section and/or with the source data.

In the pink boxes below, please ensure that the answers to the following questions are reported in the manuscript itself. Every question should be answered. If the question is not relevant to your research, please write NA (non applicable). We encourage you to include a specific subsection in the methods section for statistics, reagents, animal models and human subjects.

B- Statistics and general methods

Please fill out these boxes (Do not worry if you cannot see all your text once you press return)

1.a. How was the sample size chosen to ensure adequate power to detect a pre-specified effect size?	No statistical methods were used to pre-determine the sample size. Most experiments were done in biological triplicates, which is a normally employed sample size to ensure biological reproducibility, unless otherwise stated.
1.b. For animal studies, include a statement about sample size estimate even if no statistical methods were used.	n.a.
2. Describe inclusion/exclusion criteria if samples or animals were excluded from the analysis. Were the criteria pre-established?	No data was excluded from the study.
3. Were any steps taken to minimize the effects of subjective bias when allocating animals/samples to treatment (e.g. randomization procedure)? If yes, please describe.	To avoid potential batch effects, flasks were distributed randomly on different shakers and bacterial colonies were picked randomly from the plates after transformation. There were no other variables to randomize in this study.
For animal studies, include a statement about randomization even if no randomization was used.	n.a.
4.a. Were any steps taken to minimize the effects of subjective bias during group allocation or/and when assessing results (e.g. blinding of the investigator)? If yes please describe.	Blinding was applied only for metabolomics and proteomics measurements where sample preparation and analysis was performed by different people. For other experiments, blinding was either not possible or not relevant.
4.b. For animal studies, include a statement about blinding even if no blinding was done	n.a.
5. For every figure, are statistical tests justified as appropriate?	yes
Do the data meet the assumptions of the tests (e.g., normal distribution)? Describe any methods used to assess it.	Not tested as number of samples in each group is small.
Is there an estimate of variation within each group of data?	Yes, shown in the figures and can be estimated from the source data.

Is the variance similar between the groups that are being statistically compared?	yes

C- Reagents

6. To show that antibodies were profiled for use in the system under study (assay and species), provide a citation, catalog number and/or clone number, supplementary information or reference to an antibody validation profile. e.g., Antibodypedia (see link list at top right), 1DegreeBio (see link list at top right).	n.a.
7. Identify the source of cell lines and report if they were recently authenticated (e.g., by STR profiling) and tested for mycoplasma contamination.	n.a.

* for all hyperlinks, please see the table at the top right of the document

D- Animal Models

8. Report species, strain, gender, age of animals and genetic modification status where applicable. Please detail housing and husbandry conditions and the source of animals.	n.a.
9. For experiments involving live vertebrates, include a statement of compliance with ethical regulations and identify the committee(s) approving the experiments.	n.a.
10. We recommend consulting the ARRIVE guidelines (see link list at top right) (PLoS Biol. 8(6), e1000412, 2010) to ensure that other relevant aspects of animal studies are adequately reported. See author guidelines, under 'Reporting Guidelines'. See also: NIH (see link list at top right) and MRC (see link list at top right) recommendations. Please confirm compliance.	n.a.

E- Human Subjects

11. Identify the committee(s) approving the study protocol.	n.a.
12. Include a statement confirming that informed consent was obtained from all subjects and that the experiments conformed to the principles set out in the WMA Declaration of Helsinki and the Department of Health and Human Services Belmont Report.	n.a.
13. For publication of patient photos, include a statement confirming that consent to publish was obtained.	n.a.
14. Report any restrictions on the availability (and/or on the use) of human data or samples.	n.a.
15. Report the clinical trial registration number (at ClinicalTrials.gov or equivalent), where applicable.	n.a.
16. For phase II and III randomized controlled trials, please refer to the CONSORT flow diagram (see link list at top right) and submit the CONSORT checklist (see link list at top right) with your submission. See author guidelines, under 'Reporting Guidelines'. Please confirm you have submitted this list.	n.a.
17. For tumor marker prognostic studies, we recommend that you follow the REMARK reporting guidelines (see link list at top right). See author guidelines, under 'Reporting Guidelines'. Please confirm you have followed these guidelines.	n.a.

F- Data Accessibility

18: Provide a "Data Availability" section at the end of the Materials & Methods, listing the accession codes for data generated in this study and deposited in a public database (e.g. RNA-Seq data: Gene Expression Omnibus GSE39462, Proteomics data: PRIDE PXD000208 etc.) Please refer to our author guidelines for 'Data Deposition'.<br><br>Data deposition in a public repository is mandatory for:<br>a. Protein, DNA and RNA sequences<br>b. Macromolecular structures<br>c. Crystallographic data for small molecules<br>d. Functional genomics data<br>e. Proteomics and molecular interactions	Data availability section is included in the manuscript.
19. Deposition is strongly recommended for any datasets that are central and integral to the study; please consider the journal's data policy. If no structured public repository exists for a given data type, we encourage the provision of datasets in the manuscript as a Supplementary Document (see author guidelines under 'Expanded View' or in unstructured repositories such as Dryad (see link list at top right) or Figshare (see link list at top right).	All relevant data is available within the manuscript, the Appendix, or the submitted datasets.
20. Access to human clinical and genomic datasets should be provided with as few restrictions as possible while respecting ethical obligations to the patients and relevant medical and legal issues. If practically possible and compatible with the individual consent agreement used in the study, such data should be deposited in one of the major public access-controlled repositories such as dbGAP (see link list at top right) or EGA (see link list at top right).	n.a.
21. Computational models that are central and integral to a study should be shared without restrictions and provided in a machine-readable form. The relevant accession numbers or links should be provided. When possible, standardized format (SBML, CellML) should be used instead of scripts (e.g. MATLAB). Authors are strongly encouraged to follow the MIRIAM guidelines (see link list at top right) and deposit their model in a public database such as Biomodels (see link list at top right) or JWS Online (see link list at top right). If computer source code is provided with the paper, it should be deposited in a public repository or included in supplementary information.	All models are available from our Github repository in SBML format (see data availability section).

G- Dual use research of concern

22. Could your study fall under dual use research restrictions? Please check biosecurity documents (see link list at top right) and list of select agents and toxins (APHIS/CDC) (see link list at top right). According to our biosecurity guidelines, provide a statement only if it could.	n.a.
